# Polyploidy and introgression in invasive giant knotweed (*Fallopia sachalinensis*) during the colonization of remote volcanic islands

**DOI:** 10.1038/s41598-018-34025-2

**Published:** 2018-10-30

**Authors:** Chong-Wook Park, Gauri Shankar Bhandari, Hyosig Won, Jin Hee Park, Daniel Sangsoon Park

**Affiliations:** 1 School of Biological Sciences, Seoul National University, Seoul, 08826 Korea; 2 Department of Life Sciences, Daegu University, Gyeongsan, Gyeongbuk 38453 Korea; 3 Nakdong-gang National Institute of Biological Resources, Sangju, Gyeongbuk 37242 Korea; 4 Department of Organismic and Evolutionary Biology, Harvard University Herbaria, Cambridge, MA 20138 USA

## Abstract

Invasive giant knotweed (*Fallopia sachalinensis*) is native to northeastern Asia. In Korea, *F. sachalinensis* is confined to two volcanic islands, Ullung and Dok islands, where it occurs as dodecaploids (2n = 132). We investigated the molecular variation in 104 accessions from 94 populations of *F. sachalinensis* and its relatives throughout their native range to elucidate the origin of these island populations. All *F. sachalinensis* plants on Ullung and Dok islands were uniquely dodecaploid, whereas other populations were tetraploid (2*n* = 44). Among the 39 cpDNA haplotypes identified, the accessions from these islands shared two unique haplotypes, and were resolved as a well-supported monophyletic clade. However, this clade was sister to a clade comprising *F. japonica* accessions from southwestern Japan and separated from the clade comprising *F. sachalinensis* from other areas; this relationship is inconsistent with morphological evidence. The monophyly of the *F. sachalinensis* populations on Ullung and Dok islands suggests a single colonization event. The progenitor was likely from Japan, where it possibly captured *F. japonica* var. *japonica* cpDNA via introgression. The Ullung Island populations subsequently differentiated through polyploidization and mutations post-introduction. Our results also indicate that giant knotweed in Europe and North America likely originated from northern Japan and/or Sakhalin Island.

## Introduction

Giant knotweed, *Fallopia sachalinensis* (F. Schmidt) Ronse Decr. (Polygonaceae), belongs to sect*. Reynoutria* (Houtt.) Ronse Decr., which is distinct from the other sections in the genus by its herbaceous perennial habit, erect robust stems, well-developed thick rhizomes, large orbicular to broadly ovate leaves with acuminate to cuspidate apices, deeply three-parted styles with fimbriate stigmas, and a functionally dioecious breeding system^1,2^. The section comprises as many as 12 species that are distributed naturally in Asia including China, Korea, Japan, and the Russian Far East. However, *F. sachalinensis* and its close relative *F. japonica* (Houtt.) Ronse Decr. were introduced to Europe and North America in the 19th Century and have become widespread, noxious weeds in many countries including the United States, Canada, the United Kingdom, and most countries of northern, central and southern Europe^3–9^. In addition, they occasionally occur in Australia and New Zealand^6^.

**Figure 1:**
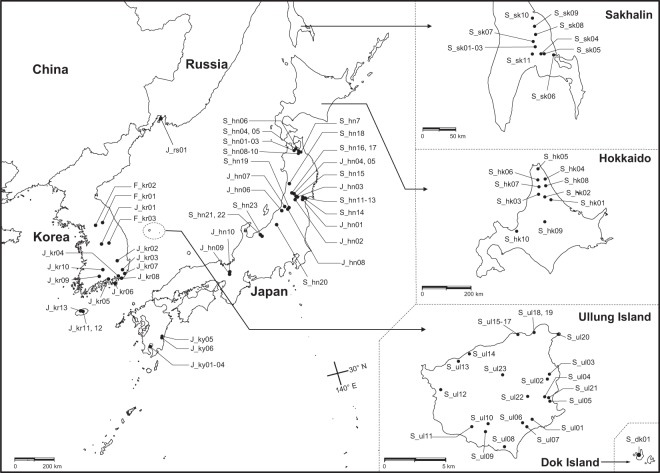
Localities of *F. sachalinensis, F. japonica* var. *japonica*, and *F. forbesii* collected from Korea, Japan, and Russia. Maps were modified from the GADM database of Global Administrative Areas v2.8^1^. The first letter in each accession name indicates the species collected, where F refers to *F. forbesii*, J to *F. japonica* var. *japonica*, and S to *F. sachalinensis*. The following lowercase two letters indicate the geographic locations of the populations; the Korean peninsula (kr), Honshu (hn), Hokkaido (hk), Kyushu (ky), Sakhalin (sk), Ullung Island (ul), and Dok Island (dk). ^1^Global Administrative Areas (2012). GADM database of Global Administrative Areas, version 2.8. https://gadm.org.

Three species belonging to sect. *Reynoutria* are found in Korea; *Fallopia sachalinensis, F. japonica*, and *F. forbesii* (Hance) Yonekura & H. Ohashi^2^. The native range of *F. sachalinensis* extends from Sakhalin Island of Russia to southern Japan (Fig. 1). It is readily distinguished from the other species in the section by its robust stems and conspicuously large ovate leaves (21.3–30.3 × 12.0–18.1 cm) with acute to acuminate apices and moderately to deeply cordate bases^2^. *F. japonica* occurs naturally in China, Korea, Japan, and the Russian Far East, and has much smaller leaves (4.9–16.2 × 3.5–10.5 cm) with cuspidate or rarely caudate apices and truncate bases. *F. forbesii* is found from southwestern China to the Korean peninsula, and is characterized by its somewhat orbicular leaves with rounded bases and short, abruptly acuminate to cuspidate apices^2^.

In Korea, *F. sachalinensis* is confined to Ullung Island and Dok Island, small, relatively young (ca. 1.8 Ma)^10^, oceanic islands of volcanic origin about 135 km and 217 km east of the main peninsula, respectively (Fig. 1). The species is relatively widespread on Ullung Island, whereas a single large population is found on Dok Island^2,11^. Several floristic studies have reported that *F. sachalinensis* was introduced to Dok Island from Ullung Island as a soil binder in 1978^11–14^. Although Ullung Island is small in size (ca. 73 km^2^), its vascular flora is relatively rich in species composition. It comprises approximately 700 species^15–17^, of which 37 taxa representing 34 genera in 25 families are endemic to the island^15^. Previous studies suggested that these Ullung Island endemics have evolved from their continental progenitors via anagenetic speciation, without subsequent cladogenetic divergence^15,18–21^. Dok Island is much smaller in size, and consists of two rocky islets, the total area of which is ca. 7.3 ha and 8.9 ha, respectively; its vascular flora comprises ca. 49 species^13^.

Our previous study on sect. *Reynoutria* in Korea demonstrated that populations of *Fallopia sachalinensis* on Ullung Island are distinct from those in other regions in having a dodecaploid chromosome number of 2*n* = 132^2^. In other regions of its range including Sakhalin Island, Japan, and Europe, only tetraploids (2n = 44), hexaploids (2n = 66), and octoploids (2n = 88) are known^2,6,22–28^. Octoploids have only been reported in limited locations in the invaded range, and possibly arose by generative reproduction via unreduced gametes or somatic mutations (i.e. autopolyploidization)^28^. While we cannot rule out the possibility that octoploid *F. sachalinensis* may exist in the native range, none have been reported to date. *F. japonica* var. *japonica* is known to comprise tetraploids, hexaploids and octoploids^6,23,25,29–33^, and *F. forbesii*, hexaploids and octoploids^2^. In the present study, we examined the sequences of the chloroplast DNA (cpDNA) regions *matK*, *ndhF*, *rbcL*, *rbcL-accD* IGS, *accD*, *accD–psaI* IGS, *trnL* intron, and *trnL–trnF* IGS from *F. sachalinensis* and closely related taxa in Korea, Japan, the Russian Far East, the United Kingdom, and the United States to (1) assess the molecular variation in *F. sachalienensis*, (2) evaluate the degree of evolutionary divergence of the Ullung Island and Dok Island populations of *F. sachalinensis* from others across the native range, and (3) elucidate their evolutionary origin and relationships to those in other regions. This study represents the most comprehensive examination of giant knotweed in its native range to date.

## Results

### Chromosome numbers

**Figure 2:**
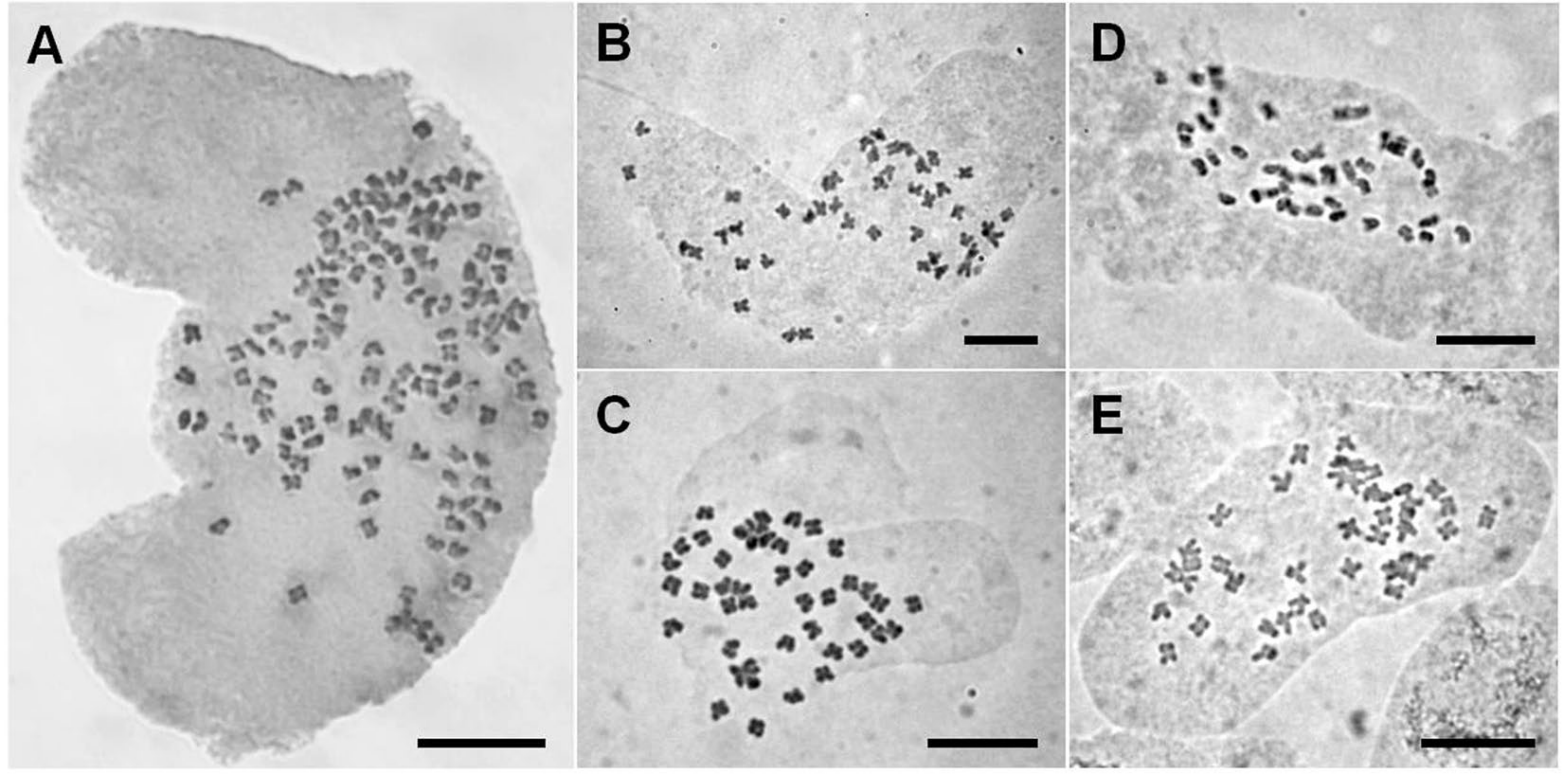
Mitotic chromosomes of representative individuals of *F. sachalinensis* and *F. japonica* var. *japonica*. Population and accession numbers correspond to those in Fig. 1 and Supplementary Table 1. (**A**) *F. sachalinensis* (2n = 132; population 4, S_ul05) (**B**). *F. sachalinensis* (2n = 44; population 23, S_sk05) (**C**). *F. sachalinensis* (2n = 44; population 34, S_hk05) (**D**). *F. sachalinensis* (2n = 44; population 54, S_hn16) (**E**). *F. japonica* var. *japonica* (2n = 44; population 86, J_ky06). Scale bar = 10 μm.

*Fallopia sachalinensis* individuals from Ullung Island and Dok Island examined for chromosome numbers in this study were dodecaploid with 2n = 132, confirming previous reports^2^ (Fig. 2, Supplementary Table 1). Our count of 2n = 132 represents the highest chromosome number in the genus. In contrast, all *F. sachalinensis* individuals examined for chromosome numbers from Japan (18 populations) and Sakhalin Island of Russia (four populations) were tetraploid with 2n = 44. The chromosome number of individuals of *F. japonica* var. *japonica* from Japan was tetraploid with 2n = 44 (Fig. 2, Supplementary Table 1).

### Nucleotide sequence variation and cpDNA haplotypes

The sequence characteristics of the examined cpDNA regions, *matK*, *ndhF*, *rbcL*, *rbcL–accD* IGS, *accD*, *accD–psaI* IGS, *trnL* intron, and *trnL–trnF* IGS, are summarized in Table 1. A total of 856 sequences of the eight cpDNA regions were obtained from 107 accessions of *Fallopia sachalinensis*, *F. japonica* var. *japonica, F. forbesii*, and three outgroup taxa (Supplementary Tables S1 and S2). The combined cpDNA data set was 8193 base pairs (bp) in length after alignment (Table 1). There were 327 (4.0%) variable characters, 194 (2.4%) of which were parsimony-informative.

**Table 1:** Statistics for the cpDNA data sets used in this study. Three outgroup taxa were included in the calculation of these statistics, with the exception of p-distance.

	*matK*	*ndhF*	*rbcL*	*rbcL–accD* IGS	*accD*	*accD–psaI* IGS	*trnL* intron	*trnL–trnF* IGS	Combined
Sequence length (bp)	1194–1200	1974	1404	478–514	1413–1437	686–703	493–540	360–363	8050–8085
Aligned length (bp)	1200	1974	1404	522	1443	732	551	367	8193
G + C ratio (%)	33.3–34.3	32.6–33.3	44.3–44.7	32.7–33.2	34.7–35.3	24.9–25.4	29.6–32.5	33.3–34.2	34.6–34.8
No. of variable characters (%)	64 (5.3)	81 (4.1)	19 (1.4)	23 (4.4)	45 (3.1)	44 (6.0)	29 (5.3)	22 (6.0)	327 (4.0)
No. of parsimony informative characters (%)	44 (3.7)	49 (2.5)	10 (0.7)	11 (2.1)	29 (2.0)	22 (3.0)	19 (3.4)	10 (2.7)	194 (2.4)
p-distance (mean)	0–0.0109 (0.0051)	0–0.0061 (0.0028)	0–0.0043 (0.0017)	0–0.0104 (0.0022)	0–0.0050 (0.0019)	0–0.0058 (0.0020)	0–0.0077 (0.0021)	0–0.0111 (0.0023)	0–0.0052 (0.0026)
MP tree length	70	92	23	27	48	45	30	22	371
No. of MP trees	18	7	13	6	2	1	1	1	8
Consistency index (CI)	0.929	0.935	0.870	0.852	0.938	0.978	1.000	1.000	0.903
Retention index (RI)	0.990	0.985	0.984	0.953	0.986	0.992	1.000	1.000	0.978
Optimal model of sequence evolution	GTR + Γ	GTR + I	HKY + I	HKY + I	GTR + I	GTR	GTR	GTR	—

Based on the combined data set, 39 haplotypes were identified from 104 accessions of *Fallopia sachalinensis*, *F. japonica* var. *japonica*, and *F. forbesii* (Supplementary Table 2). In *F. sachalinensis*, we detected 17 different haplotypes (H1–17) from 70 accessions. Among these, haplotypes 1 and 2 are found only in accessions from Ullung Island and Dok Island, Korea (Supplementary Table 2). In particular, these two haplotypes uniquely possess a five-base repeated insertion (ATTTA; bp 7516–7520) in the *trnL* intron region (Supplementary Table 2).

### Phylogenetic Analyses

**Figure 3:**
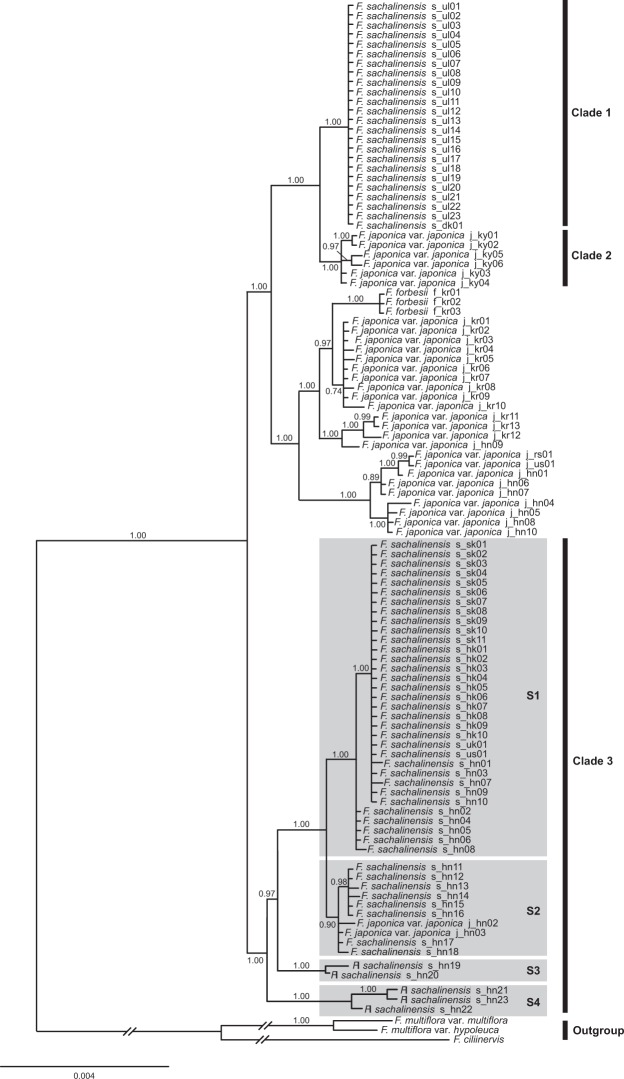
Bayesian majority-rule consensus tree for individuals of *F. sachalinensis* and closely related taxa based on the combined data set of eight cpDNA regions. Numbers above branches are Bayesian posterior probabilities (PP ≥ 0.7). Accession numbers correspond to those in Fig. 1 and Supplementary Table 1. Clade 1 comprises all accessions of *F. sachalinensis* from Ullung Island and Dok Island. Clade 2 is sister to Clade 1 and consists of *F. japonica* var. *japonica* accessions from Kyushu. Clade 3 comprises *F. sachalinensis* accessions from all other (non-Korean) populations.

The majority-rule consensus tree obtained from Bayesian inference (BI) analysis of the combined cpDNA data set is shown in Fig. 3. Maximum parsimony (MP) analysis of the combined cpDNA data set resulted in eight equally most parsimonious trees with a length of 371 steps (CI = 0.903, RI = 0.978; Table 1), and the MP strict consensus tree is shown in Fig. S1. The BI majority-rule consensus tree and the MP strict consensus tree based on the combined cpDNA data set were identical in topology and groupings (Figs. 3 and S1).

In both BI and MP trees (Figs. 3 and S1), the monophyly of *Fallopia* sect. *Reynoutria* as a whole was strongly supported (BS = 100, PP = 1.00). However, accessions of *F. sachalinensis* did not form a monophyletic group. *F. sachalinensis* accessions from Ullung Island and Dok Island were resolved as a well-supported clade (clade 1; BS = 98, PP = 1.00), but it was placed sister to a clade comprising six *F. japonica* var. *japonica* accessions from Kyushu, Japan (clade 2) with high support values (BS = 100, PP = 1.00). Accessions of *F. sachalinensis* from other regions including Japan, Sakhalin Island of Russia, the United Kingdom, and the United States formed a separate monophyletic group with two *F. japonica* var. *japonica* accessions (J_hn02, 03) from Yamagata Prefecture in central Honshu (clade 3), which was moderately supported (BS = 86, PP = 1.00). Within this group, three strongly supported *F. sachalinensis* subclades (BS ≥ 99, PP = 1.00) were identified; (1) a subclade containing 33 accessions from Hokkaido, northern Honshu (Aomori), Sakhalin, the United Kingdom, and the United States (S1), (2) a subclade containing two accessions (S_hn19, 20) from Niigata Prefecture (S3), and (3) a subclade containing three accessions (S_hn21–23) from Nagano Prefecture (S4) (Figs. 3 and S1). The remaining *F. sachalinensis* accessions, which were collected from central Honshu, formed a weakly supported subclade (BS = 56, PP = 0.90) together with two *F. japonica* var. *japonica* accessions (S2).

The other strongly supported monophyletic groups (BS = 100, PP = 1.00) in the trees included; (1) a clade consisting of *Fallopia japonica* var. *japonica* accessions from Honshu, Japan (J_hn01, 04–08, 10), Russia (J_rs01), and the United States (J_us01) and (2) a clade containing *F. forbesii* accessions (F_kr01–03).

## Discussion

*Fallopia sachalinensis* is morphologically very distinct in sect. *Reynoutria* in having tall robust stems and conspicuously large ovate leaves with moderately to deeply cordate bases. Morphologically, the plants in Ullung Island and Dok Island referable to *F. sachalinensis* are nearly indistinguishable from *F. sachalinensis* in other areas including Japan and Sakhalin Island. However, phylogenetic analyses of cpDNA data yielded an unexpected placement of the plants from Ullung Island and Dok Island (Figs. 3 and S1). In both BI and MP trees (Figs. 3 and S1), the clade comprising accessions from Ullung Island and Dok Island (clade 1) is widely separated from the clade containing *F. sachalinensis* accessions from the other areas including Japan, Russia, the United Kingdom, and the United States (clade 3). Furthermore, it is resolved as sister to the clade composed of six *F. japonica* var. *japonica* accessions from Kyushu, Japan (clade 2) with high support (BS = 100, PP = 1.00). The *F. sachalinensis* accessions from Ullung Island and Dok Island share two haloptypes (H1, 2) based on the eight cpDNA regions assessed, which were not detected in those from other examined areas (Supplementary Table 2). The haplotypes H1 and H2 differ from those found in *F. sachalinensis* accessions from the other areas including Hokkaido, Honshu, and Sakhalin Island by substitutions at 23 to 34 sites (p-distance 0.0031–0.0045) (Supplementary Table 2), suggesting that the populations of *F. sachalinensis* on Ullung Island and Dok Island are genetically very distinct from those in the other areas. In contrast, haplotypes H1 and H2 differ from those recovered from the Kyushu accessions of *F. japonica* var. *japonica* (haplotypes H35–38) by only 9–11 substituted base pairs (p-distance 0.0009–0.0011) (Supplementary Table 2). The relationships suggested by the cpDNA phylogenies, however, is inconsistent with morphological data, as the plants in Ullung Island and Dok Island are nearly identical to *F. sachalinensis* plants of lower ploidy levels in the other regions. Furthermore, the plants on Ullung Island and Dok Island are readily distinguished from those of *F. japonica* var. *japonica* by leaf size and shape^2^.

Many factors, including sampling error, incomplete lineage sorting, convergence, and hybridization/introgression may result in the incongruence between DNA and organismal phylogenies^34–36^. In the case of *Fallopia sachalinensis*, chloroplast introgression appears to be a plausible explanation for the incongruence between the morphological evidence and the topological placement of dodecaploid *F. sachalinensis* accessions from Ullung Island and Dok Island in the cpDNA molecular phylogeny (Figs. 3 and S1). In sect. *Reynoutria*, the chloroplast genome is maternally inherited^27^, and chloroplast capture through hybridization and introgression is possible as crossability barriers between the taxa of the section are not well-developed; both natural and artificial hybrids involving different varieties and cytotypes of *F. japonica* and *F. sachalinensis* have been reported^2,3,6,9,26^. Direct sequences of the second intron of nDNA *LEAFY* obtained from representative samples of *F. sachalinensis* and *F. japonica* var. *japonica* from Korea, Japan and Russia indicated the presence of many polymorphic nucleotide positions in both taxa, presumably due to gene flow between the two taxa (Appendix S1). Also, it has been reported that *F*. × *bohemica* (Chrtek & Chrtková) J. P. Bailey, a natural hybrid between *F. sachalinensis* and *F. japonica* var. *japonica* described from Europe^37^, is able to cross within and between ploidy levels, and also to backcross with any of its parents^6^.

If chloroplast capture is responsible for the incongruence observed, it is likely that the progenitor of the Ullung Island and Dok Island populations acquired *Fallopia japonica* var. *japonica* cpDNA prior to its introduction to the islands, as potential chloroplast donors such as *F. japonica* and *F. forbesii*, which often hybridize with *F. sachalinensis*^2,7,38^, are completely absent in these remote islands^2^. Rather, it is highly likely that chloroplast capture by the progenitor occurred in Japan, because (1) *F. sachalinensis* is partly sympatric with *F. japonica var. japonica* in Honshu and possibly in Kyushu, and (2) haplotypes H1 and H2 of *F. sachalinensis* accessions from Ullung Island and Dok Island appear to be sister to those recovered from Kyushu accessions of *F. japonica* var. *japonica* (haplotypes H35–38) (Figs. 3 and S1). In addition, they differ from one another by only 9–11 bp substitutions (Supplementary Table 2).

In Japan, *Fallopia sachalinensis* is naturally distributed from Hokkaido to southern Honshu including Toyama and Ishikawa Prefectures. It was also introduced to Kyushu and Shikoku as a soil binder for riverbanks^39^. *F. sachalinensis* in Japan exhibits some degree of genetic variation in chloroplast genome, and 15 haplotypes were recovered from 33 accessions representing 31 populations (Supplementary Table 2). These haplotypes fell into four major subclades in both BI and MP trees, which largely correspond to (1) northern Honshu (Aomori) and Hokkaido populations plus those from Sakhalin Island (S1; haplotypes H4–7), (2) Miyagi and Yamagata populations in central Honshu (S2; haplotypes H13–17), (3) Niigata populations in western Honshu (S3; haplotypes H11, 12), and (4) Nagano populations in southern Honshu (S4; haplotypes H8–10) (Figs. 3 and S1). All these subclades except for central Honshu subclade (S2) were strongly supported in both BI and MP trees (BS ≥ 99, PP = 1.00) (Figs. 3 and S1). The central Honshu subclade (S2) includes eight accessions of *F. sachalinensis* from Miyagi (S_hn11–14) and Yamagata (S_hn15–18) Prefectures. It also includes two *F. japonica* var. *japonica* accessions (J_hn02, 03) from Yamagata Prefecture (Figs. 3 and S1), which are morphologically very typical and distinct from *F. sachalinensis* accessions in the same subclade. Especially noteworthy is that one (J_hn03) of these two *F. japonica* var. *japonica* accessions shares the same haplotype (H17) with a *F. sachalinensis* accession (S_hn17) collected from neighboring area in the same prefecture (Figs. 1 and 3, Supplementary Table 2). In addition, the other *F. japonica* var. *japonica* accession (J_hn02) has a haplotype (H18) that differs from the haplotype H17 by only 2 bp (Supplementary Table 2). This result strongly suggests that the cytoplasmic gene flow has occurred between *F. sachalinensis* and *F. japonica* var. *japonica* plants in this region. Inamura *et al*.^40^ suggested the possibility of cytoplasmic gene flow between the two taxa in Honshu based on the analysis of *rbcL–accD* sequences. They reported two *rbcL–accD* haplotypes (*At*, *Ht*) from *F. sachalinensis* in Japan, one of which (‘*Ht’* haplotype) appeared to be nested within *F. japonica* haplotypes in their MP phylogeny. Their ‘*At*’ haplotype matches our haplotypes H3–7 represented by *F. sachalinensis* in northern Honshu, Hokkaido and on Sakhalin Island in terms of *rbcL–accD* sequences; the *‘Ht’* haplotype is close to our haplotype H9, but differs by an 1-bp substitution and an 1-bp indel.

In contrast to *Fallopia sachalinensis* plants in central and southern Honshu, those in northern Honshu (Aomori), Hokkaido and on Sakhalin Island share cpDNA haplotype H3 (Figs. 3 and S1, Supplementary Table 2), suggesting that *F. sachalinensis* plants in these areas are of common origin. The same haplotype (H3) is also shared by accessions from the United Kingdom and the United States examined in this study. Furthermore, the *rbcL–accD* sequence of *F. sachalinensis* accessions from northern Honshu (Aomori), Hokkaido and Sakhalin Island appears to be identical to those reported from *F. sachalinensis* plants in the United States^8^. Based on our data, it is highly likely that *F. sachalinensis* plants were introduced to Europe and North America from northeastern Asia, presumably northern Japan (northern Honshu/Hokkaido) and/or Sakhalin Island. Our data parallel the hypothesis regarding sources of *F. sachalinensis* in the British Isles suggested by Pashley *et al*.^22^ On the basis of RFLP data from *trnC–trnD*/*trnF–trnV* regions, they recognized two haplotypes (MPH 1, 6) in British *F. sachalinensis* populations and suggested that the MPH 6 haplotype, which is found in most populations, was probably introduced from northern Japan via St. Petersburg, Russia, since it was detected in accessions from Hokkaido. However, they did not examine the Russian plants, which share the same haplotype in the eight chloroplast DNA regions with Hokkaido ones, and the possibility that the MPH 6 haplotype in the British Isles could have been introduced from Sakhalin Island cannot be ruled out.

Especially noteworthy is that there is almost no variation in the cpDNA *matK*, *ndhF*, *rbcL*, *rbcL–accD* IGS, *accD*, *accD–psaI* IGS, *trnL* intron, and *trnL–trnF* IGS regions among 23 accessions representing 19 populations of *Fallopia sachalinensis* in Ullung Island. Only two individuals from population 14 differed by a single-bp (A) insertion at position 4916 in the *rbcL–accD* IGS region (Supplementary Table 2). The low genetic variation in the above loci in Ullung Island populations of *F. sachalinensis* may be indicative of a relatively recent colonization event, and lack of subsequent gene flow from populations in other areas. Indeed, Ullung Island is located about 135 km east of the mainland and about 275 km west from Japan, and hence is more or less isolated from the major source areas of gene flow. The low level of variation in these loci in Ullung Island populations also might be associated with clonal spread by extensive rhizomes as in *F. sachalinensis* individuals of the British Isles^22,38^.

Based on 62 chromosome counts of the native and endemic species of Ullung Island, Weiss *et al*.^20^ suggested that virtually no changes in ploidy level or dysploidy have occurred during differentiation of most endemic taxa of Ullung Island, and all progenitor and derivative taxa have exactly the same chromosome number. In contrast, all *Fallopia sachalinensis* plants examined on Ullung Island appear to be dodecaploid with 2n = 132, whereas those collected from the other areas in the present study are all tetraploid with 2*n* = 44 (Fig. 2, Supplementary Table 1). *F. sachalinensis* on Dok Island are also dodecaploid (Supplementary Table 1), providing support for previous reports^11–14^ that it was introduced from Ullung Island. The dodecaploid count is the highest chromosome number known in the genus, and only tetraploids, hexaploids, and octoploids have been reported so far in other regions of its range, including Japan, the Russian Far East, and Europe^2,6,22–28,41^. On this basis, we postulate that the polyploidization of *F. sachalinensis* plants on Ullung Island occurred post-colonization, and the dodecaploid plants likely arose via the union of reduced and unreduced gametes from the tetraploid cytotypes followed by doubling of chromosome number. It has been noted that the Ullung Island populations are capable of sexual reproduction, and pistillate individuals produce a large number of seeds every year^2^. Indeed, such polyploidization may have facilitated the colonization of the island, and similar events in other populations could potentially facilitate further spread of giant knotweed in its invaded range^42^.

Along these lines, it is possible that dodecaploid *Fallopia sachalinensis* arose as a result of allopolyploidy involving tetraploid and octoploid cytotypes of *F. sachalinensis* and *F. japonica* var. *japonica* in Japan, then was subsequently introduced to Ullung Island. Indeed, polyploidy has been shown to facilitate long-distance dispersal^43^, and it is possible that undiscovered populations of dodecaploid *F. sachalinensis* exist in Japan. Under this hypothesis, the topological placement of dodecaploid *F. sachalinensis* accessions in the cpDNA phylogeny (Figs. 3 and S1) could be explained by inheritance from a maternal *F. japonica* var. *japonica* progenitor. However, we were unable to find any evidence of dodecaploid population(s) of *F. sachalinensis* in Japan, or in any other regions within its range despite extensive field surveys (Fig. 1). Further, we also conducted additional phylogenetic analyses using the sequences of the second intron of nDNA *LEAFY* for a subset of the ingroup taxa to gain more insight into the origin of the progenitor of the Ullung Island/Dok Island populations (Appendix S1). In the *LEAFY* BI phylogeny (Fig. SA1), all *F. sachalinensis* accessions and three haplotypes recovered from *F. japonica* var. *japonica* in Japan were resolved as a single well-supported clade (PP = 0.93). In particular, the haplotypes recovered from the *F. sachalinensis* accessions from Ullung Island and Dok Island were nested within those from *F. sachalinensis* in other regions (Fig. SA1), suggesting that the *F. sachalinensis* plants on Ullung Island and Dok Island are not significantly different in their nuclear genome from those in other regions. This result, in conjunction with morphological evidence that individuals of *F. sachalinensis* on Ullung Island and Dok Island are indistinguishable from those in other areas, provides support for our hypothesis that the progenitor of the Ullung Island/Dok Island populations captured *F. japonica* var. *japonica* cpDNA via introgression.

In conclusion, our results provide further insights into the origin and degree of molecular divergence of the Ullung Island and Dok Island populations of *Fallopia sachalinensis*. The monophyly of the Ullung Island and Dok Island populations of *F. sachalinensis* strongly suggest that they originated from a single introduction. Our results are also in agreement with previous reports that the *F. sachalinensis* population on Dok Island was introduced from Ullung Island. The founder population was most likely introduced to Ullung Island from Japan, because (1) Ullung Island is of volcanic origin and relatively young (ca. 1.8 Ma)^10^, (2) has no known connection with the mainland, (3) *F. sachalinensis* does not occur naturally on the Korean Peninsula^2^, and (4) the haplotypes recovered from the *F. sachalinensis* accessions from Ullung Island and Dok Island appear to be sister to those from Kyushu accessions of *F. japonica* var. *japonica* (Figs. 3 and S1). Based on our data, it is likely that the progenitor of the Ullung Island/Dok Island populations had captured *F. japonica* var. *japonica* cpDNA prior to its introduction to the island in Japan, where *F. sachalinensis* is partly sympatric with *F. japonica* var. *japonica*. Indeed, our both our cpDNA and nDNA analyses suggest cytoplasmic gene flow occurs, if infrequently, between the two taxa in Japan. Genetic differentiation of the Ullung Island populations probably arose through mutations and polyploidization post-introduction, since the cpDNA haplotypes found in the *F. sachalinensis* populations on Ullung and Dok islands were not detected in possible source areas. However, our results are mainly based on cpDNA and limited nDNA analyses and further studies examining large numbers of single- or low-copy nuclear genes using next-generation sequencing (NGS) approaches^44^ among the populations of *F. sachalinensis* in Japan, particularly in western and southern Honshu, would help elucidate the exact origin of the Ullung Island populations.

## Methods

### Taxon sampling

We sampled 68 individuals of *Fallopia sachalinensis* from 20 populations on Ullung Island and Dok Island of Korea, nine on Sakhalin Island of Russia, and 31 in Japan representing the entire native range of the species (Fig. 1, Supplementary Table 1). Two additional samples of *F. sachalinensis* obtained from the United States and the United Kingdom were also examined. In particular, extensive fieldwork was carried out in Hokkaido, Honshu, Kyushu, and Sakhalin Island by the authors in 2007, 2011, 2012, and 2014. At least one or two individuals from each population were transplanted from the field to the greenhouse and/or the experimental garden at Seoul National University whenever possible.

In addition, we examined 34 accessions of *Fallopia japonica* var. *japonica* and *F. forbesii* from Korea, Japan, Russia, and the United States to determine the relationship of *F. sachalinensis* to the latter two taxa (Fig. 1, Supplementary Table 1). Three taxa of sect. *Fallopia*, *F. ciliinervis* (Nakai) K. Hammer, *F. multiflora* (Thunb.) Haraldson var. *multiflora* and var. *hypoleuca* (Nakai ex Ohwi) Yonekura & H. Ohashi, were selected as outgroups on the basis of relationships suggested by previous studies on the genus *Fallopia*^2,26^. All voucher specimens were deposited in the Seoul National University Herbarium (SNU).

### Chromosome counts

Mitotic chromosome numbers of 48 individuals from 45 populations of *Fallopia sachalinensi*s, *F. japonica* var. *japonica*, and *F. forbesii* were examined (Supplementary Table 1). Root tips were pretreated in 0.2% colchicine solution for 3 hr at room temperature, fixed in acetic alcohol (glacial acetic acid:ethanol, 1:3, v/v) for 15 min, and softened for 8–10 min in 1 N HCl solution at 60 °C using water bath. Root tips were then stained and squashed in 1% acetic orcein solution^2^, and chromosome preparations were observed and photographed with an Olympus BX-50 microscope at 1000–2000×.

### DNA extraction, amplification, and sequencing

Total genomic DNA was extracted from leaf samples, either fresh or dried with silica gel, using the DNeasy plant mini kit (Qiagen, Germany). Eight regions of cpDNA, *matK*, *ndhF*, *rbcL*, *rbcL–accD* IGS, *accD*, *accD–psaI* IGS, *trnL* intron, and *trnL–trnF* IGS, were amplified by polymerase chain reaction (PCR). Amplifications were carried out using a GeneAmp PCR system 2400 or a Veriti 96-well thermal cycler (Applied Biosystems, USA) in 50 µl total volume containing 20–50 ng of template DNA, 1.5 units of *Taq* polymerase (Roche, Germany), 5 µL of 10× PCR buffer with 1.5 mmol/L MgCl_2_, 0.1 µmol/L of each dNTP, 5% DMSO, and 0.1 µmol/L of each primer. PCR and sequencing primers and PCR cycling conditions used in this study are provided in Table 2. The PCR products were purified using the enzymatic purification method described by Werle *et al*. (1994)^45^. Purified PCR products were sequenced using the ABI Prism BigDye^®^ terminator v 3.1 cycle sequencing kit (Applied Biosystems, USA) following the manufacturer’s instructions. The sequenced products were purified by ethanol precipitation, and were run on an ABI Prism 3730 genetic analyzer (Applied Biosystems, USA) at Seoul National University.

**Table 2:** PCR/sequencing primers and PCR cycling conditions for eight cpDNA regions examined in this study. Primer names follow the original publications.

Region	PCR/sequencing primers		PCR cycling condition (35 cycles)				
	Forward primer	Reverse primer	Pre-denaturation (3 min)	Denaturation (1 min)	Annealing (40 s)	Extension (45 s)	Final extension (7 min)
*matK*	670F^a^ 193F^a^	1246R^a^ 479R^a^	95 °C	95 °C	52 °C	72 °C	72 °C
*ndhF*	1^b^ 7F^a^	1314R^a^ 2110R^a^	95 °C	95 °C	50 °C	72 °C	72 °C
*rbcL*	1F^c^ 1141F^c^	712R^c^ 1376R^c^	95 °C	95 °C	50 °C	72 °C	72 °C
*rbcL–accD* IGS	1141F^c^	2442R^c^	95 °C	95 °C	50 °C	72 °C	72 °C
*accD*	RA1F^d^	accDA1R^d^	95 °C	95 °C	53 °C	72 °C	72 °C
*accD–psaI* IGS	accD2644F^a^	psaI75R^e^	95 °C	95 °C	58 °C	72 °C	72 °C
*trnL* intron	c^f^	d^f^	95 °C	95 °C	54 °C	72 °C	72 °C
*trnL–trnF* IGS	e2^a^	f^f^	95 °C	95 °C	54 °C	72 °C	72 °C

^a^Present study; ^b^Olmstead and Sweere^52^; ^c^Yasui and Ohnishi^53^; ^d^Inamura *et al*.^40^; ^e^Shaw *et al*.^54^; ^f^Taberlet *et al*.^55^.

### Sequence alignment and analyses

Nucleotide sequences were assembled and edited using Sequencher 4.7 (Gene Codes Co., USA). Edited sequences were aligned with Clustal X v. 1.83^46^ with final manual adjustment using Se-Al v. 2.0a11^47^. All DNA sequences obtained in this study were deposited in GenBank (Supplementary Table 1).

Phylogenetic analyses were performed on the individual and combined cpDNA sequence data sets using maximum parsimony (MP) and Bayesian inference (BI). Initial phylogenetic analyses of the individual data sets (*matK*, *ndhF*, *rbcL*, *rbcL–accD* IGS, *accD, accD–psaI* IGS, *trnL* intron, and *trnL–trnF* IGS) did not provide sufficient resolution for *F. sachalinensis* populations. Pairwise comparisons of the above sequence data sets using the incongruence length difference test as implemented in PAUP* 4.0b10^48^ indicated no significant incongruences among these regions, and therefore they were combined for subsequent analyses.

MP analyses were performed in PAUP* using a heuristic search strategy with 100 random sequence additions, tree bisection-reconnection (TBR) branch swapping, ACTRAN, STEEPEST DESCENT, MULTREES on, MAXTREE set to no limit, and HOLD = 10 in effect. All characters were treated as unordered and equally weighted, and gaps were treated as missing data. One poly-A region in *accD–psaI* IGS (bp 6917–6930), which shows extensive length variations, was excluded from the analyses. Bootstrap (BS) analyses^49^ of 1000 replicates were conducted in PAUP* to evaluate support for clades using the same search parameters as in the MP analyses above. For BI analyses, the optimal model of sequence evolution for each data set was identified using the Akaike information criterion (AIC) in MrModeltest 2.3^50^. The following models of sequence evolution were identified as optimal for the eight cpDNA regions examined in this study; GTR + Γ for *matK*, GTR + I for *ndhF* and *accD*, HKY + I for *rbcL* and *rbcL–accD* IGS, and GTR for *accD–psaI* IGS, *trnL* intron and *trnL–trnF* IGS (Table 1). The BI analysis of the combined data set was performed in MrBayes 3.2^51^ using two independent runs of four chains (three heated and one cold) for one million generations. Trees were sampled every 1000 generations, and the first 25% were discarded as burn-in. The remaining trees were used to produce a 50% majority-rule consensus tree and determine posterior probabilities (PP). See Appendix S1 for methods regarding nDNA analyses.

## Electronic supplementary material

Supplementary Information

## Data Availability

All sequence data have been deposited in GenBank.
