# Supplementary material for: Polyploidy and introgression in invasive giant knotweed (Fallopia sachalinensis) during the colonization of remote volcanic islands
Source: Sci Rep. 2018 Oct 30;8:16021. doi: 10.1038/s41598-018-34025-2 (PMC6207670; doi:10.1038/s41598-018-34025-2)
Supplement: Supplementary file 1 — Supplementary Information [file 41598_2018_34025_MOESM1_ESM.pdf]

Supplementary Information for:

Polyploidy and introgression in invasive giant knotweed (*Fallopia sachalinensis*) during the colonization of remote volcanic islands

Chong-Wook Park[1*], Gauri Shankar Bhandari[1], Hyosig Won[2], Jin Hee Park[3], Daniel S. Park[4*]

[1] School of Biological Sciences, Seoul National University, Seoul 08826, Korea.

[2] Department of Life Sciences, Daegu University, Gyeongsan, Gyeongbuk 38453, Korea.

[3] Nakdong-gang National Institute of Biological Resources, Sangju, Gyeongbuk 37242, Korea.

[4] Department of Organismic and Evolutionary Biology, Harvard University Herbaria, Cambridge, MA 20138, USA.

*SI correspondence to:
Chong-Wook Park, parkc@snu.ac.kr
Daniel S. Park, danielpark@fas.harvard.edu

Contents: Figure S1, Tables S1 – S2, Appendix S1

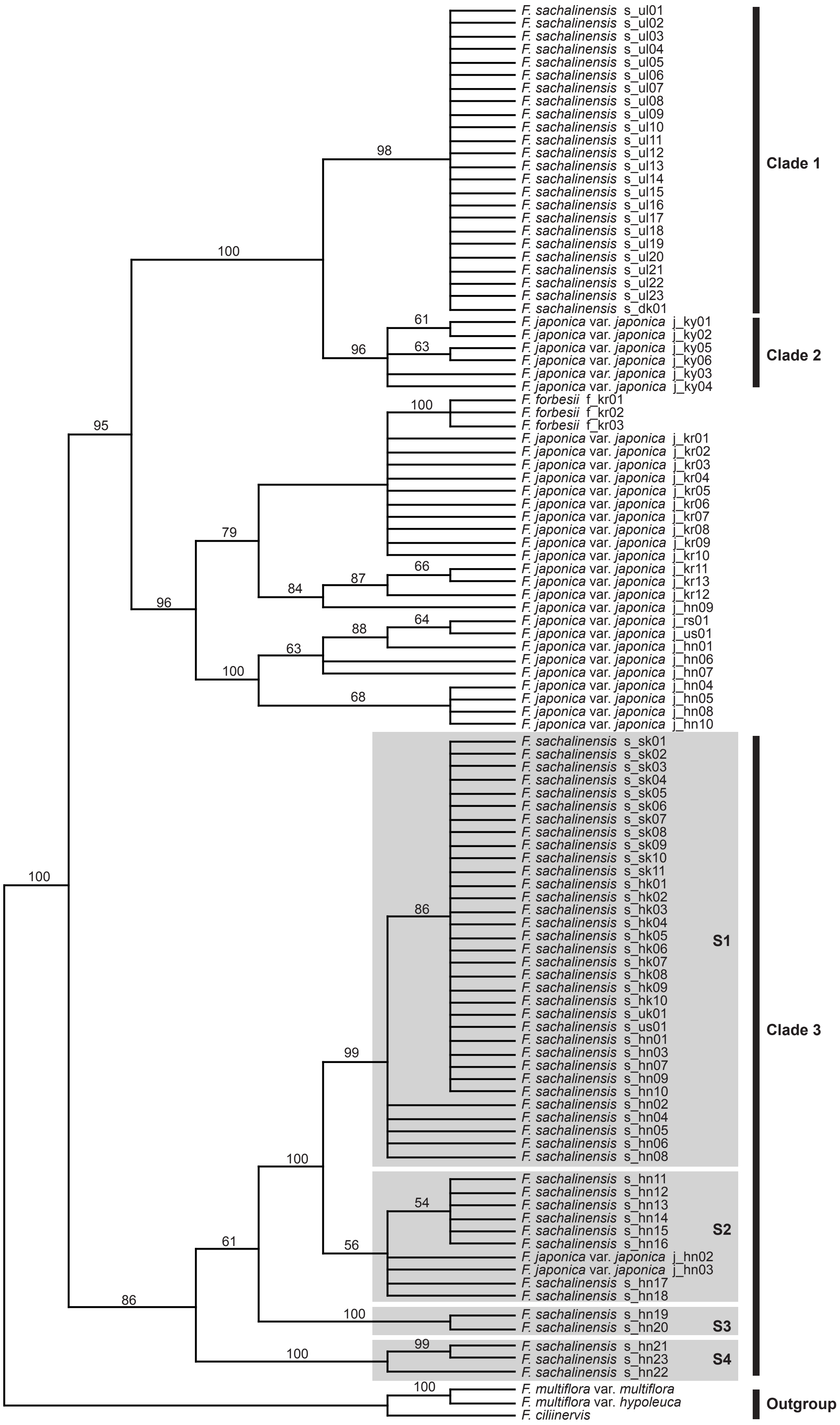

Figure S1. Strict consensus tree of eight equally most parsimonious trees for individuals of *F. sachalinensis* and closely related taxa based on the combined data set of eight cpDNA regions. Numbers above branches indicate parsimony bootstrap (BS ≥ 50) values. Accession numbers correspond to those in Supplementary Table 1.

Table S1. Voucher information, chromosome numbers, and GenBank accession numbers for 107 accessions of *F. sachalinensis* and closely related taxa examined in this study (outgroup taxa included). All vouchers are deposited in SNU unless otherwise cited. See Fig. 1 for specific localities of the accessions sampled in Korea, Japan, and Russia.

Population	Accession	Locality and voucher	Chromosome number (2n)	GenBank accession number (*matK*, *ndhF*, *rbcL*, *rbcL-accD* IGS, *accD*, *accD-psaI* IGS, *trnL* intron, *trnL-trnF* IGS)
F. sachalinensis				
P1	S_ul01	Korea. Gyeongbuk, Ullung Island, Sadong, *J. Y. Kim 406*	132[a, b]	KJ863074, KJ863181, KJ863288, KJ862967, KJ887157, KJ887264, KJ887371, KJ887050
P2	S_ul02	Korea. Gyeongbuk, Ullung Island, Jeodong, Naesujeon, near a mineral spring, *S. K. Lee 08037*	132[a]	KJ863075, KJ863182, KJ863289, KJ862968, KJ887158, KJ887265, KJ887372, KJ887051
P3	S_ul03	Korea. Gyeongbuk, Ullung Island, Jeodong, Naesujeon, roadside near observatory, *S. K. Lee 08038*	132[a]	KJ863076, KJ863183, KJ863290, KJ862969, KJ887159, KJ887266, KJ887373, KJ887052
P4	S_ul04	Korea. Gyeongbuk, Ullung Island, roadside near Jeodong Primary School, *S. K. Lee 04017*	-	KJ863077, KJ863184, KJ863291, KJ862970, KJ887160, KJ887267, KJ887374, KJ887053
	S_ul05	*S. K. Lee 08033*	132[a]	KJ863078, KJ863185, KJ863292, KJ862971, KJ887161, KJ887268, KJ887375, KJ887054
P5	S_ul06	Korea. Gyeongbuk, Ullung Island, Sadong, *S. K. Lee 08039*	132[a, b]	KJ863079, KJ863186, KJ863293, KJ862972, KJ887162, KJ887269, KJ887376, KJ887055
P6	S_ul07	Korea. Gyeongbuk, Ullung Island, Sadong, along Okcheon stream, *S. K. Lee 08040*	132[a, b]	KJ863080, KJ863187, KJ863294, KJ862973, KJ887163, KJ887270, KJ887377, KJ887056

Table S1. (continued).

Population	Accession	Locality and voucher	Chromosome number (2n)	GenBank accession number (*matK*, *ndhF*, *rbcL*, *rbcL-accD* IGS, *accD*, *accD-psaI* IGS, *trnL* intron, *trnL-trnF* IGS)
P7	S_ul08	Korea. Gyeongbuk, Ullung Island, Namyang, Gameulgye, *S. K. Lee 08041*	-	KJ863081, KJ863188, KJ863295, KJ862974, KJ887164, KJ887271, KJ887378, KJ887057
P8	S_ul09	Korea. Gyeongbuk, Ullung Island, Namyang, Seokmundong, *S. K. Lee 08042*	-	KJ863082, KJ863189, KJ863296, KJ862975, KJ887165, KJ887272, KJ887379, KJ887058
P9	S_ul10	Korea. Gyeongbuk, Ullung Island, Namyang, 1 km from Namyang Bridge, *S. K. Lee 08043*	-	KJ863083, KJ863190, KJ863297, KJ862976, KJ887166, KJ887273, KJ887380, KJ887059
P10	S_ul11	Korea. Gyeongbuk, Ullung Island, along Namseo stream, *S. K. Lee 08044*	-	KJ863084, KJ863191, KJ863298, KJ862977, KJ887167, KJ887274, KJ887381, KJ887060
P11	S_ul12	Korea. Gyeongbuk, Ullung Island, Taeha, *S. K. Lee 08045*	-	KJ863085, KJ863192, KJ863299, KJ862978, KJ887168, KJ887275, KJ887382, KJ887061
P12	S_ul13	Korea. Gyeongbuk, Ullung Island, Hyeonpo, *S. K. Lee 08046*	-	KJ863086, KJ863193, KJ863300, KJ862979, KJ887169, KJ887276, KJ887383, KJ887062
P13	S_ul14	Korea. Gyeongbuk, Ullung Island, Pyeongri, *S. K. Lee 08047*	-	KJ863087, KJ863194, KJ863301, KJ862980, KJ887170, KJ887277, KJ887384, KJ887063
P14	S_ul15	Korea. Gyeongbuk, Ullung Island, Chunbu, Jukam, near Mongdol Beach, *S. K. Lee 08048*	132[a]	KJ863088, KJ863195, KJ863302, KJ862981, KJ887171, KJ887278, KJ887385, KJ887064
	S_ul16	*J. H. Park & K. Kim 102*	-	KJ863092, KJ863199, KJ863306, KJ862985, KJ887175, KJ887282, KJ887389, KJ887068
	S_ul17	*J. H. Park & K. Kim 106*	132[a]	KJ863096, KJ863203, KJ863310, KJ862989, KJ887179, KJ887286, KJ887393, KJ887072

Table S1. (continued).

Population	Accession	Locality and voucher	Chromosome number (2n)	GenBank accession number (*matK*, *ndhF*, *rbcL*, *rbcL-accD* IGS, *accD*, *accD-psaI* IGS, *trnL* intron, *trnL-trnF* IGS)
P15	S_ul18	Korea. Gyeongbuk, Ullung Island, Chunbu, Jukam Village, *S. K. Lee 08049*	132[a]	KJ863089, KJ863196, KJ863303, KJ862982, KJ887172, KJ887279, KJ887386, KJ887065
	S_ul19	*J. H. Park & K. Kim 113*	-	KJ863093, KJ863200, KJ863307, KJ862986, KJ887176, KJ887283, KJ887390, KJ887069
P16	S_ul20	Korea. Gyeongbuk, Ullung Island, Seommok, *S. K. Lee 08050*	-	KJ863090, KJ863197, KJ863304, KJ862983, KJ887173, KJ887280, KJ887387, KJ887066
P17	S_ul21	Korea. Gyeongbuk, Ullung Island, Jeodong, *J. H. Park & K. Kim 114*	-	KJ863094, KJ863201, KJ863308, KJ862987, KJ887177, KJ887284, KJ887391, KJ887070
P18	S_ul22	Korea. Gyeongbuk, Ullung Island, Jeodong, Bongrae Waterfall, *J. H. Park & K. Kim 6*	132[b]	KJ863091, KJ863198, KJ863305, KJ862984, KJ887174, KJ887281, KJ887388, KJ887067
P19	S_ul23	Korea. Gyeongbuk, Ullung Island, Naribunji, *J. H. Park & K. Kim 126*	132[b]	KJ863095, KJ863202, KJ863309, KJ862988, KJ887178, KJ887285, KJ887392, KJ887071
P20	S_dk01	Korea. Gyeongbuk, Dok Island, *B. Y. Sun s.n.*	132[a]	KJ863028, KJ863135, KJ863242, KJ862921, KJ887111, KJ887218, KJ887325, KJ887004
P21	S_sk01	Russia. Sakhalin Island, Yuzhno-Sakhalinsk, *V. Teslenko 2008-1*	-	KJ863062, KJ863169, KJ863276, KJ862955, KJ887145, KJ887252, KJ887359, KJ887038
	S_sk02	*V. Teslenko 2008-2*	-	KJ863063, KJ863170, KJ863277, KJ862956, KJ887146, KJ887253, KJ887360, KJ887039
	S_sk03	*V. Teslenko 2008-3*	-	KJ863064, KJ863171, KJ863278, KJ862957, KJ887147, KJ887254, KJ887361, KJ887040
P22	S_sk04	Russia. Sakhalin Island, Bolnichnaya, *C.-W. Park & K. Kim 2011-1*	44[a]	KJ863066, KJ863173, KJ863280, KJ862959, KJ887149, KJ887256, KJ887363, KJ887042

Table S1. (continued).

Population	Accession	Locality and voucher	Chromosome number (2n)	GenBank accession number (*matK*, *ndhF*, *rbcL*, *rbcL-accD* IGS, *accD*, *accD-psaI* IGS, *trnL* intron, *trnL-trnF* IGS)
P23	S_sk05	Russia. Sakhalin Island, Bolnichnaya, *C.-W. Park & K. Kim 2011-2*	44[a]	KJ863070, KJ863177, KJ863284, KJ862963, KJ887153, KJ887260, KJ887367, KJ887046
P24	S_sk06	Russia. Sakhalin Island, Korsakovskiy, *C.-W. Park & K. Kim 2011-4*	44[a]	KJ863067, KJ863174, KJ863281, KJ862960, KJ887150, KJ887257, KJ887364, KJ887043
P25	S_sk07	Russia. Sakhalin Island, Lugovoye, *C.-W. Park & K. Kim 2011-8*	44[a]	KJ863065, KJ863172, KJ863279, KJ862958, KJ887148, KJ887255, KJ887362, KJ887041
P26	S_sk08	Russia. Sakhalin Island, Sovetskoye, *C.-W. Park & K. Kim 2011-12*	-	KJ863068, KJ863175, KJ863282, KJ862961, KJ887151, KJ887258, KJ887365, KJ887044
P27	S_sk09	Russia. Sakhalin Island, Sovetskoye, *C.-W. Park & K. Kim 2011-13*	-	KJ863071, KJ863178, KJ863285, KJ862964, KJ887154, KJ887261, KJ887368, KJ887047
P28	S_sk10	Russia. Sakhalin Island, Dolinskiy, *C.-W. Park & K. Kim 2011-18*	-	KJ863072, KJ863179, KJ863286, KJ862965, KJ887155, KJ887262, KJ887369, KJ887048
P29	S_sk11	Russia. Sakhalin Island, Troizkoe, *C.-W. Park & K. Kim 2011-27*	-	KJ863069, KJ863176, KJ863283, KJ862962, KJ887152, KJ887259, KJ887366, KJ887045
P30	S_hk01	Japan. Hokkaido, Nayoro, *C.-W. Park & J. H. Park 11-1*	-	KJ863029, KJ863136, KJ863243, KJ862922, KJ887112, KJ887219, KJ887326, KJ887005
P31	S_hk02	Japan. Hokkaido, Uryu, Horokanai-cho, *C.-W. Park et al. 11-3-1*	-	KJ863030, KJ863137, KJ863244, KJ862923, KJ887113, KJ887220, KJ887327, KJ887006
P32	S_hk03	Japan. Hokkaido, Uryu, Uryu Experimental Forest of Hokkaido University, *C.-W. Park et al. 11-5-1*	-	KJ863031, KJ863138, KJ863245, KJ862924, KJ887114, KJ887221, KJ887328, KJ887007
P33	S_hk04	Japan. Hokkaido, Teshio, Teshio Experimental Forest of Hokkaido University, *C.-W. Park et al. 11-9-1*	44[a]	KJ863032, KJ863139, KJ863246, KJ862925, KJ887115, KJ887222, KJ887329, KJ887008

Table S1. (continued).

Population	Accession	Locality and voucher	Chromosome number (2n)	GenBank accession number (*matK*, *ndhF*, *rbcL*, *rbcL-accD* IGS, *accD*, *accD-psaI* IGS, *trnL* intron, *trnL-trnF* IGS)
P34	S_hk05	Japan. Hokkaido, Teshio, Toyotomi-cho, *C.-W. Park et al. 11-11-1*	44[a]	KJ863033, KJ863140, KJ863247, KJ862926, KJ887116, KJ887223, KJ887330, KJ887009
P35	S_hk06	Japan. Hokkaido, Teshio, Horonobe-cho, *C.-W. Park et al. 11-12-2*	44[a]	KJ863034, KJ863141, KJ863248, KJ862927, KJ887117, KJ887224, KJ887331, KJ887010
P36	S_hk07	Japan. Hokkaido, Nakagawa, Nakagawa-cho, *C.-W. Park et al. 11-13-1*	-	KJ863035, KJ863142, KJ863249, KJ862928, KJ887118, KJ887225, KJ887332, KJ887011
P37	S_hk08	Japan. Hokkaido, Nakagawa, Nakagawa Experimental Forest of Hokkaido University, *C.-W. Park et al. 11-15-3*	-	KJ863036, KJ863143, KJ863250, KJ862929, KJ887119, KJ887226, KJ887333, KJ887012
P38	S_hk09	Japan. Hokkaido, Kamikawa, *C.-W. Park et al. 11-17-1*	-	KJ863037, KJ863144, KJ863251, KJ862930, KJ887120, KJ887227, KJ887334, KJ887013
P39	S_hk10	Japan. Hokkaido, Sapporo, *C.-W. Park et al. 11-18-3*	-	KJ863038, KJ863145, KJ863252, KJ862931, KJ887121, KJ887228, KJ887335, KJ887014
P40	S_hn01	Japan. Honshu, Aomori, along Arakawa River (40˚ 47' 26.24" N, 140˚ 45' 11.04" E), *C.-W. Park 370*	44[a]	KJ863040, KJ863147, KJ863254, KJ862933, KJ887123, KJ887230, KJ887337, KJ887016
P41	S_hn02	Japan. Honshu, Aomori, along Arakawa River (40˚ 47' 23.76" N, 140˚ 45' 09.34" E), *C.-W. Park 371*	44[a]	KJ863041, KJ863148, KJ863255, KJ862934, KJ887124, KJ887231, KJ887338, KJ887017
P42	S_hn03	Japan. Honshu, Aomori, along Arakawa River (40˚ 47' 20.01" N, 140˚ 45' 06.87" E), *C.-W. Park 372*	44[a]	KJ863042, KJ863149, KJ863256, KJ862935, KJ887125, KJ887232, KJ887339, KJ887018
P43	S_hn04	Japan. Honshu, Aomori, along Nonai River (40˚ 50' 31.77" N, 140˚ 49' 11.04" E), *C.-W. Park 373*	44[a]	KJ863043, KJ863150, KJ863257, KJ862936, KJ887126, KJ887233, KJ887340, KJ887019

Table S1. (continued).

Population	Accession	Locality and voucher	Chromosome number (2n)	GenBank accession number (*matK*, *ndhF*, *rbcL*, *rbcL-accD* IGS, *accD*, *accD-psaI* IGS, *trnL* intron, *trnL-trnF* IGS)
P44	S_hn05	Japan. Honshu, Aomori, along Nonai River (40˚ 50' 31.06" N, 140˚ 49' 07.41" E), *C.-W. Park 374*	44[a]	KJ863044, KJ863151, KJ863258, KJ862937, KJ887127, KJ887234, KJ887341, KJ887020
P45	S_hn06	Japan. Honshu, Aomori, Mt. Hakkoda, N slope along Rt. 40, *C.-W. Park 378*	44[a]	KJ863045, KJ863152, KJ863259, KJ862938, KJ887128, KJ887235, KJ887342, KJ887021
P46	S_hn07	Japan. Honshu, Aomori, Mt. Hakkoda, Tashirotai area, *C.-W. Park 379*	44[a]	KJ863046, KJ863153, KJ863260, KJ862939, KJ887129, KJ887236, KJ887343, KJ887022
P47	S_hn08	Japan. Honshu, Aomori, Mt. Hakkoda Botanic Garden of Tohoku University, streamside, *C.-W. Park 381*	44[a]	KJ863047, KJ863154, KJ863261, KJ862940, KJ887130, KJ887237, KJ887344, KJ887023
P48	S_hn09	Japan. Honshu, Aomori, Mt. Hakkoda Botanic Garden of Tohoku University, swampy area, *C.-W. Park 382*	44[a]	KJ863048, KJ863155, KJ863262, KJ862941, KJ887131, KJ887238, KJ887345, KJ887024
P49	S_hn10	Japan. Honshu, Aomori, Mt. Hakkoda Botanic Garden of Tohoku University, roadside near field station, *C.-W. Park 383*	44[a]	KJ863049, KJ863156, KJ863263, KJ862942, KJ887132, KJ887239, KJ887346, KJ887025
P50	S_hn11	Japan. Honshu, Miyagi, Sendai, roadside near Sendai City Museum, *C.-W. Park 384-1*	-	KJ863050, KJ863157, KJ863264, KJ862943, KJ887133, KJ887240, KJ887347, KJ887026
P51	S_hn12	Japan. Honshu, Miyagi, Sendai, along Hirose River near Ohashi Bridge, *C.-W. Park 384-2*	-	KJ863051, KJ863158, KJ863265, KJ862944, KJ887134, KJ887241, KJ887348, KJ887027
	S_hn13	*C.-W. Park 384-3*	-	KJ863052, KJ863159, KJ863266, KJ862945, KJ887135, KJ887242, KJ887349, KJ887028
P52	S_hn14	Japan. Honshu, Miyagi, Sendai, Tohoku University campus, *K. Yonekura s.n.*	-	KJ863039, KJ863146, KJ863253, KJ862932, KJ887122, KJ887229, KJ887336, KJ887015

Table S1. (continued).

Population	Accession	Locality and voucher	Chromosome number (2n)	GenBank accession number (*matK*, *ndhF*, *rbcL*, *rbcL-accD* IGS, *accD*, *accD-psaI* IGS, *trnL* intron, *trnL-trnF* IGS)
P53	S_hn15	Japan. Honshu, Yamagata, Yamadera, *C.-W. Park & G. S. Bhandari 387*	44[a]	KJ863053, KJ863160, KJ863267, KJ862946, KJ887136, KJ887243, KJ887350, KJ887029
P54	S_hn16	Japan. Honshu, Yamagata, Sagae (38˚ 24' 16.38" N, 140˚ 13' 56.55" E), *C.-W. Park & G. S. Bhandari 391*	44[a]	KJ863054, KJ863161, KJ863268, KJ862947, KJ887137, KJ887244, KJ887351, KJ887030
P55	S_hn17	Japan. Honshu, Yamagata, Sagae (38˚ 24' 15.99" N, 140˚ 13' 56.77" E), *C.-W. Park & G. S. Bhandari 392*	44[a]	KJ863055, KJ863162, KJ863269, KJ862948, KJ887138, KJ887245, KJ887352, KJ887031
P56	S_hn18	Japan. Honshu, Yamagata, Mt. Chokai, *Y. Kadota s.n.*	-	KJ863061, KJ863168, KJ863275, KJ862954, KJ887144, KJ887251, KJ887358, KJ887037
P57	S_hn19	Japan. Honshu, Niigata, Shekigawa-mura, *C.-W. Park & G. S. Bhandari 402*	-	KJ863059, KJ863166, KJ863273, KJ862952, KJ887142, KJ887249, KJ887356, KJ887035
P58	S_hn20	Japan. Honshu, Niigata, Minami-Uonuma-gun, *Y. Kadota s.n.*	-	KJ863060, KJ863167, KJ863274, KJ862953, KJ887143, KJ887250, KJ887357, KJ887036
P59	S_hn21	Japan. Honshu, Nagano, roadside near Himekawa Hot Spring, *C.-W. Park & G. S. Bhandari 395-1*	44[a]	KJ863056, KJ863163, KJ863270, KJ862949, KJ887139, KJ887246, KJ887353, KJ887032
	S_hn22	*C.-W. Park & G. S. Bhandari 395-2*	44[a]	KJ863057, KJ863164, KJ863271, KJ862950, KJ887140, KJ887247, KJ887354, KJ887033
P60	S_hn23	Japan. Honshu, Nagano, Kitaotari, *C.-W. Park & G. S. Bhandari 396*	44[a]	KJ863058, KJ863165, KJ863272, KJ862951, KJ887141, KJ887248, KJ887355, KJ887034
P61	S_uk01	U. K. Leicester (transplanted in SNU), *J. Y. Kim 678*	44[a]	KJ863073, KJ863180, KJ863287, KJ862966, KJ887156, KJ887263, KJ887370, KJ887049
P62	S_us01	U. S. A. WA, Klickitat Co., *R. R. Halse 4549*	-	KJ863097, KJ863204, KJ863311, KJ862990, KJ887180, KJ887287, KJ887394, KJ887073

Table S1. (continued).

Population	Accession	Locality and voucher	Chromosome number (2n)	GenBank accession number (*matK*, *ndhF*, *rbcL*, *rbcL-accD* IGS, *accD*, *accD-psaI* IGS, *trnL* intron, *trnL-trnF* IGS)
F. japonica* var. *japonica				
P63	J_kr01	Korea. Chungbuk, Mt. Doota, *W.-K. Paik 3202* (KB)	-	KJ863019, KJ863126, KJ863233, KJ862912, KJ887102, KJ887209, KJ887316, KJ886995
P64	J_kr02	Korea. Gyeongbuk, Mt. Palgong, *J. Y. Kim 556*	88[b]	KJ863008, KJ863115, KJ863222, KJ862901, KJ887091, KJ887198, KJ887305, KJ886984
P65	J_kr03	Korea. Gyeongnam, Mt. Yeongchwi, *M. H. Kim & J. Y. Kim 712*	-	KJ863017, KJ863124, KJ863231, KJ862910, KJ887100, KJ887207, KJ887314, KJ886993
P66	J_kr04	Korea. Gyeongnam, Mt. Dotdae, *D. K. Kim & S. H. Hwang 358162-080* (KB)	-	KJ863018, KJ863125, KJ863232, KJ862911, KJ887101, KJ887208, KJ887315, KJ886994
P67	J_kr05	Korea. Gyeongnam, Mt. Mooryang, *C.-W. Park et al. 131024-1*	-	KJ863016, KJ863123, KJ863230, KJ862909, KJ887099, KJ887206, KJ887313, KJ886992
P68	J_kr06	Korea. Gyeongnam, Geoje Isl., *K. Kim et al. 120526-2*	-	KJ863014, KJ863121, KJ863228, KJ862907, KJ887097, KJ887204, KJ887311, KJ886990
P69	J_kr07	Korea. Busan, Kijang-gun, *S. K. Lee 31*	66[a]	KJ863013, KJ863120, KJ863227, KJ862906, KJ887096, KJ887203, KJ887310, KJ886989
P70	J_kr08	Korea. Busan, Mt. Bongnae, *D. K. Kim & S. H. Hwang 359133-059* (KB)	-	KJ863015, KJ863122, KJ863229, KJ862908, KJ887098, KJ887205, KJ887312, KJ886991
P71	J_kr09	Korea. Jeonnam, Mt. Baekun, *S. K. Lee 05005*	88[a, b]	KJ863012, KJ863119, KJ863226, KJ862905, KJ887095, KJ887202, KJ887309, KJ886988
P72	J_kr10	Korea. Jeonnam, Mt. Jiri, *S. K. Lee 05007*	-	KJ863011, KJ863118, KJ863225, KJ862904, KJ887094, KJ887201, KJ887308, KJ886987

Table S1. (continued).

Population	Accession	Locality and voucher	Chromosome number (2n)	GenBank accession number (*matK*, *ndhF*, *rbcL*, *rbcL-accD* IGS, *accD*, *accD-psaI* IGS, *trnL* intron, *trnL-trnF* IGS)
P73	J_kr11	Korea. Jeju, Kyorae, near Kyorae Bridge, *J. Y. Kim 559*	88[b]	KJ863007, KJ863114, KJ863221, KJ862900, KJ887090, KJ887197, KJ887304, KJ886983
	J_kr12	*J. H. Park et al. 2*	88[a]	KJ863010, KJ863117, KJ863224, KJ862903, KJ887093, KJ887200, KJ887307, KJ886986
P74	J_kr13	Korea. Jeju, Ara-dong, *J. Y. Kim 511*	88[a, b]	KJ863009, KJ863116, KJ863223, KJ862902, KJ887092, KJ887199, KJ887306, KJ886985
P75	J_hn01	Japan. Honshu, Miyagi, Sendai, S of Sendai Municipal Museum, *K. Yonekura s.n.*	-	KJ862999, KJ863106, KJ863213, KJ862892, KJ887082, KJ887189, KJ887296, KJ886975
P76	J_hn02	Japan. Honshu, Yamagata, Yamagata-zao, *C.-W. Park & G. S. Bhandari 385*	-	KJ863000, KJ863107, KJ863214, KJ862893, KJ887083, KJ887190, KJ887297, KJ886976
P77	J_hn03	Japan. Honshu, Yamagata, Yamadera, *C.-W. Park & G. S. Bhandari 386*	-	KJ863001, KJ863108, KJ863215, KJ862894, KJ887084, KJ887191, KJ887298, KJ886977
P78	J_hn04	Japan. Honshu, Yamagata, Yamagatagawa, (38˚ 23' 00.01", N 140˚ 19' 44.16" E) *C.-W. Park & G. S. Bhandari 388*	-	KJ863002, KJ863109, KJ863216, KJ862895, KJ887085, KJ887192, KJ887299, KJ886978
P79	J_hn05	Japan. Honshu, Yamagata, Yamagatagawa, (38˚ 23' 10.21", N 140˚ 20' 07.51" E) *C.-W. Park & G. S. Bhandari 390*	-	KJ863003, KJ863110, KJ863217, KJ862896, KJ887086, KJ887193, KJ887300, KJ886979
P80	J_hn06	Japan. Honshu, Niigata, Shibata-nishi, *G. S. Bhandari 400*	-	KJ863004, KJ863111, KJ863218, KJ862897, KJ887087, KJ887194, KJ887301, KJ886980
P81	J_hn07	Japan. Honshu, Niigata, Sekikawa-mura, Oshima, *G. S. Bhandari 401*	-	KJ863005, KJ863112, KJ863219, KJ862898, KJ887088, KJ887195, KJ887302, KJ886981

Table S1. (continued).

Population	Accession	Locality and voucher	Chromosome number (2n)	GenBank accession number (*matK*, *ndhF*, *rbcL*, *rbcL-accD* IGS, *accD*, *accD-psaI* IGS, *trnL* intron, *trnL-trnF* IGS)
P82	J_hn08	Japan. Honshu, Niigata, Sekikawa-mura, near Takanoshu Dam, 25 Aug 2012, *G. S. Bhandari 402*	-	KJ863006, KJ863113, KJ863220, KJ862899, KJ887089, KJ887196, KJ887303, KJ886982
P83	J_hn09	Japan. Honshu, Kyoto, Kyoto-shi, Kyoto University campus, *H.-S. Won 376*	44[a]	KJ862997, KJ863104, KJ863211, KJ862890, KJ887080, KJ887187, KJ887294, KJ886973
P84	J_hn10	Japan. Honshu, Kyoto, Kyoto-shi, Mt. Kurama, *H.-S. Won 377*	44[a]	KJ862998, KJ863105, KJ863212, KJ862891, KJ887081, KJ887188, KJ887295, KJ886974
P85	J_ky01	Japan. Kyushu, Kagoshima, Sakurajima, Arimura observation point, *C.-W. Park 8-1*	44[a]	KJ863022, KJ863129, KJ863236, KJ862915, KJ887105, KJ887212, KJ887319, KJ886998
	J_ky02	*C.-W. Park 8-4*	44[a]	KJ863020, KJ863127, KJ863234, KJ862913, KJ887103, KJ887210, KJ887317, KJ886996
P86	J_ky03	Japan. Kyushu, Kagoshima, Sakurajima, Sakurajimayokohama-cho, roadside, *C.-W. Park 8-5*	44[a]	KJ863021, KJ863128, KJ863235, KJ862914, KJ887104, KJ887211, KJ887318, KJ886997
P87	J_ky04	Japan. Kyushu, Kagoshima, Sakurajima, Yunohira observation point, *C.-W. Park 8-6-1*	44[a]	KJ863024, KJ863131, KJ863238, KJ862917, KJ887107, KJ887214, KJ887321, KJ887000
P88	J_ky05	Japan. Kyushu, Miyazaki, Miyazaki-shi, *S. Masami B*	44[a]	KJ863023, KJ863130, KJ863237, KJ862916, KJ887106, KJ887213, KJ887320, KJ886999
P89	J_ky06	Japan. Kyushu, Miyazaki, Saito-shi, *S. Masami D2*	44[a]	KJ863025, KJ863132, KJ863239, KJ862918, KJ887108, KJ887215, KJ887322, KJ887001
P90	J_rs01	Russia. Vladivostok, *C.-W. Park s.n.*	44[a]	KJ863026, KJ863133, KJ863240, KJ862919, KJ887109, KJ887216, KJ887323, KJ887002
P91	J_us01	U. S. A. NY, Tully, *C.-W. Park s.n.*	-	KJ863027, KJ863134, KJ863241, KJ862920, KJ887110, KJ887217, KJ887324, KJ887003

Table S1. (continued).

Population	Accession	Locality and voucher	Chromosome number (2n)	GenBank accession number (*matK*, *ndhF*, *rbcL*, *rbcL-accD* IGS, *accD*, *accD-psaI* IGS, *trnL* intron, *trnL-trnF* IGS)
F. forbesii				
P92	F_kr01	Korea. Gangwon, Chuncheon, *J. Y. Kim 706*	88[b]	KJ862994, KJ863101, KJ863208, KJ862887, KJ887077, KJ887184, KJ887291, KJ886970
P93	F_kr02	Korea. Gyeonggi, Kwangreung, *J. Y. Kim 565*	88[b]	KJ862995, KJ863102, KJ863209, KJ862888, KJ887078, KJ887185, KJ887292, KJ886971
P94	F_kr03	Korea. Chungbuk, Mt. Worak, *J. Y. Kim 567*	88[a, b]	KJ862996, KJ863103, KJ863210, KJ862889, KJ887079, KJ887186, KJ887293, KJ886972
**Outgroup**				
F. multiflora var. *multiflora*				
-	MUL	Korea. Gyeongnam, Hadong, *J. H. Park s.n.*	-	KJ863100, KJ863205, KJ863313, KJ862993, KJ887183, KJ887290, KJ887397, KJ887076
F. multiflora var. *hypoleuca*				
-	HYP	Taiwan. Taichuang, Houli Hsiang, *M. J. Yoo et al. 26*	-	KJ863098, KJ863207, KJ863314, KJ862992, KJ887182, KJ887289, KJ887396, KJ887075
F. ciliinervis				
-	CLV	Korea. Gangwon, Mt. Taebaek, *K. Kim et al. 480*	-	KJ863099, KJ863206, KJ863312, KJ862991, KJ887181, KJ887288, KJ887395, KJ887074

[a] Present study

[b] Kim and Park (2000)

Table S2. Variable nucleotide sites for cpDNA haplotypes recovered from 104 accessions of *F. sachalinensis* and closely related taxa (outgroup taxa excluded). Accession numbers refer to those in Table S1.

		Variable nucleotide site in alignment																					
		matK																					
Haplo-type	Accession	0049	0060	0070	0152	0263	0266	0307	0318	0328	0342	0347	0430	0434	0522	0560	0581	0694	0712	0721	0775	0776	0777
H1	S_ul01-14, 16, 18-23, dk01	G	C	C	G	G	A	G	G	T	A	C	T	G	C	A	T	T	A	G	–	–	–
H2	S_ul15, 17	·	·	·	·	·	·	·	·	·	·	·	·	·	·	·	·	·	·	·	–	–	–
H3	S_sk01-11, us01, uk01, hk01-10, hn03, 09, 10	·	T	T	·	T	C	·	A	·	C	·	·	·	T	·	·	·	·	·	G	T	A
H4	S_hn01	·	T	T	·	T	C	·	A	·	C	·	·	·	T	·	·	·	·	·	G	T	A
H5	S_hn02, 04-06	·	T	T	·	T	C	·	A	·	C	·	·	·	T	·	·	·	·	·	G	T	A
H6	S_hn07	·	T	T	·	T	C	·	A	·	C	·	·	·	T	·	·	·	·	·	G	T	A
H7	S_hn08	·	T	T	·	T	C	·	A	·	C	·	·	·	T	·	·	·	·	·	G	T	A
H8	S_hn21	A	T	T	·	·	·	·	·	C	·	·	·	·	·	·	G	·	·	·	G	T	A
H9	S_hn22	A	T	T	·	·	·	·	·	C	·	·	·	·	·	·	G	·	·	·	G	T	A
H10	S_hn23	A	T	T	·	·	·	·	·	C	·	·	·	·	·	·	G	·	·	·	G	T	A
H11	S_hn19	·	T	T	·	·	·	·	·	·	·	A	G	·	·	·	·	·	·	A	G	T	A
H12	S_hn20	·	T	T	·	·	·	·	·	·	·	A	G	·	·	·	·	·	·	A	G	T	A
H13	S_hn14	·	T	T	A	·	·	·	·	·	C	·	·	·	T	·	·	·	·	·	G	T	A
H14	S_hn11, 12, 15, 16	·	T	T	A	·	·	·	·	·	C	·	·	·	T	·	·	·	·	·	G	T	A
H15	S_hn13	·	T	T	A	·	·	·	·	·	C	·	·	·	T	·	·	·	·	·	G	T	A
H16	S_hn18	·	T	T	A	·	·	·	·	·	C	·	·	·	T	G	·	·	·	·	G	T	A
H17	S_hn17, J_hn03	·	T	T	A	·	·	·	·	·	C	·	·	·	T	·	·	·	·	·	G	T	A
H18	J_hn02	·	T	T	A	·	·	·	·	·	C	·	·	·	T	·	·	·	·	·	G	T	A
H19	J_hn09	·	T	·	·	·	·	·	·	·	·	·	·	·	·	·	·	·	·	·	–	–	–
H20	J_hn10	·	T	·	·	T	·	·	·	·	·	·	·	·	·	·	·	·	·	·	–	–	–
H21	J_hn01	T	T	·	·	·	·	·	·	·	·	·	·	·	·	·	·	·	·	·	–	–	–
H22	J_hn04	·	T	·	·	T	·	·	·	·	·	·	·	·	·	·	·	·	·	·	–	–	–
H23	J_hn05	·	T	·	·	T	·	·	·	·	·	·	·	·	·	·	·	·	·	·	–	–	–
H24	J_hn06, 07	·	T	·	·	·	·	·	·	·	·	·	·	·	·	·	·	·	·	·	–	–	–
H25	J_hn08	·	T	·	·	T	·	·	·	·	·	·	·	·	·	·	·	·	·	·	–	–	–
H26	J_kr11	·	T	·	·	T	·	·	·	·	·	·	·	·	·	·	·	·	·	·	–	–	–
H27	J_kr01-03, 06, 07, 09	·	T	·	·	·	C	·	·	·	·	·	·	A	·	·	·	·	·	·	–	–	–
H28	J_kr13	·	T	·	·	T	·	·	·	·	·	·	·	·	·	·	·	·	·	·	–	–	–
H29	J_kr12	·	T	·	·	T	C	·	·	·	·	·	·	·	·	·	·	·	·	·	–	–	–
H30	J_kr10	·	T	·	·	·	C	·	·	·	·	·	·	A	·	·	·	·	·	·	–	–	–
H31	J_kr05	·	T	·	·	·	C	A	·	·	·	·	·	A	·	·	·	·	·	·	–	–	–
H32	J_kr04	·	T	·	·	·	C	·	·	·	·	·	·	A	·	·	·	·	·	·	–	–	–
H33	J_kr08	·	T	·	·	·	C	·	·	·	·	·	·	A	·	·	·	·	·	·	–	–	–
H34	J_rs01, J_us01	T	T	·	·	·	·	·	·	·	·	·	·	·	·	·	·	C	·	·	–	–	–
H35	J_ky01, 02	·	T	·	·	·	·	·	·	·	·	·	·	·	·	·	·	·	C	·	–	–	–
H36	J_ky03, 04	·	T	·	·	·	·	·	·	·	·	·	·	·	·	·	·	·	C	·	–	–	–
H37	J_ky05	·	T	·	·	·	·	·	·	·	·	·	·	·	·	·	·	·	C	·	–	–	–
H38	J_ky06	·	T	·	·	·	·	·	·	·	·	·	·	·	·	·	·	·	C	·	–	–	–
H39	F_kr01-03	·	T	·	·	T	C	·	·	·	·	·	·	·	·	·	·	·	·	·	–	–	–

Table S2. (continued).

Haplotype	Variable nucleotide site in alignment																																						
	matK												*ndhF*																										
	0778	0779	0780	0781	0839	0897	0928	0960	0973	0991	1031	1032	1224	1294	1299	1551	1914	1992	2037	2155	2286	2306	2325	2382	2388	2407	2487	2495	2544	2727	2733	2775	2796	2821	2823	2829	2848	2871	2882
H1	–	–	–	G	G	T	A	C	T	G	C	T	T	T	A	A	T	A	C	T	A	C	G	T	C	A	T	A	C	C	G	T	A	A	C	G	T	A	T
H2	–	–	–	·	·	·	·	·	·	·	·	·	·	·	·	·	·	·	·	·	·	·	·	·	·	·	·	·	·	·	·	·	·	·	·	·	·	·	·
H3	T	T	T	A	·	·	·	A	·	·	·	C	·	·	T	·	·	·	·	·	·	·	A	·	T	·	·	·	A	·	·	·	G	·	A	·	·	·	·
H4	T	T	T	A	·	·	·	A	·	·	·	C	·	·	T	·	·	·	·	·	·	·	A	·	T	·	·	·	A	·	·	·	G	·	A	·	·	·	·
H5	T	T	T	A	·	·	·	A	·	·	·	C	·	·	T	·	·	·	·	·	·	·	A	·	T	·	·	·	A	·	·	·	G	·	A	·	·	·	·
H6	T	T	T	A	·	·	·	A	·	·	·	C	·	·	T	·	·	·	·	·	·	·	A	·	T	·	·	·	A	·	·	·	G	·	A	·	·	·	·
H7	T	T	T	A	·	·	·	A	·	·	·	C	·	·	T	·	·	·	·	·	·	·	A	·	T	·	·	·	A	·	·	·	G	·	A	·	·	·	·
H8	T	T	T	A	·	·	·	·	·	·	·	·	·	·	T	·	·	·	·	·	·	·	A	G	T	·	·	·	A	·	·	·	·	·	A	·	G	C	·
H9	T	T	T	A	A	·	·	·	·	·	·	·	·	·	T	·	·	·	·	·	·	·	A	G	T	·	·	·	A	·	·	·	·	·	A	·	G	C	·
H10	T	T	T	A	·	·	·	·	·	·	·	·	·	·	T	·	·	·	·	·	·	·	A	G	T	·	·	·	A	·	·	G	·	·	A	·	G	C	·
H11	T	T	T	A	·	A	·	·	·	·	A	·	·	·	T	·	·	·	·	·	·	·	A	·	T	·	·	·	A	·	·	·	·	G	A	A	·	·	·
H12	T	T	T	A	·	A	·	·	·	·	·	·	·	·	T	·	·	·	·	·	·	·	A	·	T	·	·	·	A	·	·	·	·	·	A	·	·	·	·
H13	T	T	T	A	·	·	·	A	·	·	·	C	·	·	T	·	·	C	·	·	·	·	A	·	T	·	·	·	A	·	·	·	G	·	A	·	·	·	·
H14	T	T	T	A	·	·	·	A	·	·	·	C	·	·	T	·	·	C	·	·	·	·	A	·	T	·	·	·	A	·	·	·	G	·	A	·	·	·	·
H15	T	T	T	A	·	·	·	A	·	·	·	C	·	·	T	·	·	C	·	·	·	·	A	·	T	·	·	·	A	·	·	·	G	·	A	·	·	·	·
H16	T	T	T	A	·	·	·	A	·	·	·	C	·	·	T	·	·	·	·	·	·	·	A	·	T	·	·	·	A	·	·	·	G	·	A	·	·	·	·
H17	T	T	T	A	·	·	·	A	·	·	·	C	·	·	T	·	·	·	·	·	·	·	A	·	T	·	·	·	A	·	·	·	G	·	A	·	·	·	·
H18	T	T	T	A	·	·	·	A	·	·	·	C	·	·	T	·	·	·	·	·	·	·	A	·	T	·	·	G	A	·	·	·	G	·	A	·	·	·	·
H19	–	–	–	·	·	·	·	·	·	·	·	·	·	·	T	T	C	·	·	C	·	·	A	·	·	·	·	·	A	T	·	·	·	·	A	·	·	·	·
H20	–	–	–	·	·	·	·	·	C	A	·	·	·	·	T	·	C	·	T	C	·	T	A	·	·	·	·	·	A	·	A	·	·	·	A	·	·	·	·
H21	–	–	–	·	·	·	·	·	C	A	·	·	·	·	T	·	C	·	T	C	·	T	A	·	·	·	·	·	A	·	A	G	·	·	A	·	·	·	·
H22	–	–	–	·	·	·	·	·	C	A	·	·	·	·	T	·	C	·	T	C	·	T	A	·	·	·	·	·	A	·	A	·	·	·	A	·	·	·	·
H23	–	–	–	·	·	·	·	·	C	A	·	·	·	·	T	·	C	·	T	C	·	T	A	·	·	·	·	·	A	·	A	·	·	·	A	·	·	·	·
H24	–	–	–	·	·	·	·	·	C	A	·	·	·	·	T	·	C	·	T	C	·	T	A	·	·	·	·	·	A	·	A	G	·	·	A	·	·	·	·
H25	–	–	–	·	·	·	·	·	C	A	·	·	·	·	T	·	C	·	T	C	·	T	A	·	·	·	·	·	A	·	A	·	·	·	A	·	·	·	·
H26	–	–	–	·	·	·	C	·	·	·	·	·	·	·	T	·	C	·	·	C	·	·	A	·	·	G	·	·	A	·	·	·	·	·	A	·	·	·	G
H27	–	–	–	·	·	·	·	·	·	·	·	·	·	·	T	·	C	·	·	C	·	·	A	·	·	·	·	·	A	·	·	·	·	·	A	·	·	·	·
H28	–	–	–	·	·	·	C	·	·	·	·	·	·	·	T	·	C	·	·	C	·	·	A	·	·	G	·	·	A	·	·	·	·	·	A	·	·	·	G
H29	–	–	–	·	·	·	·	·	·	·	·	·	·	·	T	·	C	·	·	C	·	·	A	·	·	G	·	·	A	·	·	·	·	·	A	·	·	·	G
H30	–	–	–	·	·	·	·	·	·	·	·	·	·	·	T	·	C	·	·	C	·	·	A	·	·	·	·	·	A	·	A	G	·	·	A	·	·	·	·
H31	–	–	–	·	·	·	·	·	·	·	·	·	·	·	T	·	C	·	·	C	·	·	A	·	·	·	·	·	A	·	·	·	·	·	A	·	·	·	·
H32	–	–	–	·	·	·	·	·	·	·	·	·	·	·	T	·	C	·	·	C	·	·	A	·	·	·	·	·	A	·	·	·	·	·	A	·	·	·	·
H33	–	–	–	·	·	·	·	·	·	·	·	·	·	C	T	·	C	·	·	C	·	·	A	·	·	·	·	·	A	·	·	·	·	·	A	·	·	·	·
H34	–	–	–	·	·	·	·	·	C	A	·	·	·	·	T	·	C	·	T	C	·	T	A	·	·	·	·	·	A	·	A	G	·	·	A	·	·	·	·
H35	–	–	–	·	·	·	·	·	·	·	·	·	·	·	·	·	·	·	·	·	·	·	A	·	·	·	C	·	A	·	·	·	·	·	·	·	·	·	·
H36	–	–	–	·	·	·	·	·	·	·	·	·	·	·	·	·	·	·	·	·	·	·	A	·	·	·	C	·	A	·	·	·	·	·	·	·	·	·	·
H37	–	–	–	·	·	·	·	·	·	·	·	·	·	·	·	·	·	·	·	·	G	·	A	·	·	·	C	·	A	·	·	·	·	·	·	·	·	·	·
H38	–	–	–	·	·	·	·	·	·	·	·	·	A	·	·	·	·	·	·	·	G	·	A	·	·	·	C	·	A	·	·	·	·	·	·	·	·	·	·
H39	–	–	–	·	·	·	·	·	·	·	·	·	·	·	T	·	C	·	·	C	·	·	A	·	·	·	·	·	A	·	·	G	·	G	A	·	·	·	·

Table S2. (continued).

Haplo-type	*ndh*F			*rbcL*							*rbcL-accD* IGS									*accD*																	
	2295	2938	2979	3248	3256	3407	3537	3861	4251	4266	4594	4632	4633	4634	4820	4915	4916	5072	5073	5115	5139	5140	5425	5426	5427	5428	5429	5430	5437	5438	5443	5452	5467	5472	5474	5558	5605
H1	C	G	C	A	A	G	C	A	G	C	T	T	T	T	A	A	A	T	T	G	A	G	–	–	–	–	–	–	T	C	C	G	A	T	G	A	T
H2	·	·	·	·	·	·	·	·	·	·	·	·	·	·	·	·	–	·	·	·	·	·	–	–	–	–	–	–	·	·	·	·	·	·	·	·	·
H3	T	·	·	·	·	A	·	G	·	T	C	·	·	·	·	·	–	–	–	·	G	·	–	–	–	–	–	–	G	A	·	T	·	·	·	·	C
H4	T	·	·	·	·	A	·	G	·	T	C	·	·	·	·	·	–	–	–	·	G	·	–	–	–	–	–	–	G	A	·	T	·	·	·	·	C
H5	T	·	·	·	·	A	·	G	·	T	C	·	·	·	·	·	–	–	–	·	G	·	–	–	–	–	–	–	·	·	·	T	·	·	·	·	C
H6	T	·	·	·	·	A	·	G	·	T	C	·	·	·	·	·	–	–	–	·	G	·	–	–	–	–	–	–	G	A	·	T	·	·	·	·	C
H7	T	·	·	·	·	A	·	G	·	T	C	·	·	·	·	·	–	–	–	C	G	·	–	–	–	–	–	–	·	·	·	T	·	·	·	·	C
H8	T	·	·	·	·	A	·	G	·	T	·	A	A	A	·	–	–	–	–	·	G	A	–	–	–	–	–	–	·	·	·	T	G	·	·	C	C
H9	T	·	·	·	·	A	·	·	·	T	·	·	·	·	·	·	–	–	–	·	G	·	–	–	–	–	–	–	·	·	·	T	G	·	·	C	C
H10	T	·	·	·	·	A	·	·	·	T	·	A	A	A	·	–	–	–	–	·	G	A	–	–	–	–	–	–	·	·	·	T	G	·	·	C	C
H11	T	·	·	·	·	A	·	·	·	T	·	·	·	·	·	·	–	·	–	·	G	·	–	–	–	–	–	–	·	·	·	T	·	G	·	·	C
H12	T	·	·	·	·	A	·	·	·	T	·	·	·	·	·	·	–	–	–	·	G	·	–	–	–	–	–	–	·	·	·	T	·	G	·	·	C
H13	T	·	·	·	·	A	·	G	·	T	C	·	·	·	·	·	–	–	–	·	G	·	–	–	–	–	–	–	·	·	·	T	·	·	·	·	C
H14	T	·	·	·	·	A	·	G	·	T	C	·	·	·	·	·	–	–	–	·	G	·	–	–	–	–	–	–	·	·	·	T	·	·	·	·	C
H15	T	·	·	·	·	A	·	·	·	T	C	·	·	·	·	·	–	–	–	·	G	·	–	–	–	–	–	–	·	·	·	T	·	·	·	·	C
H16	T	·	·	·	·	A	·	G	·	T	C	·	·	·	·	·	–	–	–	·	G	·	–	–	–	–	–	–	·	·	·	T	·	·	·	·	C
H17	T	·	·	·	·	A	·	G	·	T	C	·	·	·	·	·	–	–	–	·	G	·	–	–	–	–	–	–	·	·	·	T	·	·	·	·	C
H18	T	·	·	·	·	A	·	G	·	T	C	·	·	·	·	·	–	–	–	·	G	·	–	–	–	–	–	–	·	·	·	T	·	·	·	·	C
H19	T	·	G	C	·	A	T	·	A	T	·	A	A	A	·	·	–	·	–	·	G	·	–	–	–	–	–	–	·	·	·	T	·	·	·	·	·
H20	T	·	·	C	·	·	·	·	A	T	·	·	·	·	C	·	–	·	·	·	G	·	–	–	–	–	–	–	·	·	A	T	·	·	C	·	·
H21	T	·	·	C	·	·	·	·	A	T	·	·	·	·	C	·	–	·	·	·	G	·	–	–	–	–	–	–	·	·	A	T	·	·	C	·	·
H22	T	·	·	C	·	·	·	·	A	T	·	A	A	A	C	·	–	·	·	·	G	·	–	–	–	–	–	–	·	·	A	T	·	·	C	·	·
H23	T	·	·	C	·	·	·	·	A	T	·	·	·	·	C	·	–	·	·	·	G	·	–	–	–	–	–	–	·	·	A	T	·	·	C	·	·
H24	T	·	·	C	·	·	·	·	A	T	·	·	·	·	C	·	–	·	·	·	G	·	–	–	–	–	–	–	·	·	A	T	·	·	C	·	·
H25	T	·	·	C	·	·	·	·	A	T	·	·	·	·	C	·	–	·	·	·	G	·	T	G	T	T	T	T	·	·	A	T	·	·	C	·	·
H26	T	·	G	C	·	A	T	·	A	T	·	A	A	A	·	·	·	–	–	·	G	·	–	–	–	–	–	–	·	·	·	T	·	·	·	·	·
H27	T	·	G	C	·	A	T	·	A	T	·	·	·	·	·	·	–	·	–	·	G	·	–	–	–	–	–	–	·	·	·	T	·	·	·	·	·
H28	T	·	G	C	·	A	T	·	A	T	·	A	A	A	·	·	–	–	–	·	G	·	–	–	–	–	–	–	·	·	·	T	·	·	·	·	·
H29	T	·	G	C	·	A	T	·	A	T	·	A	A	A	·	·	·	–	–	·	G	·	–	–	–	–	–	–	·	·	·	T	·	·	·	·	·
H30	T	·	·	C	·	A	T	·	A	T	·	·	·	·	·	·	–	·	–	·	G	·	–	–	–	–	–	–	·	·	·	T	·	·	·	·	·
H31	T	·	G	C	·	A	T	·	A	T	·	·	·	·	·	·	–	·	–	·	G	·	–	–	–	–	–	–	·	·	·	T	·	·	·	·	·
H32	T	·	G	C	C	A	T	·	A	T	·	·	·	·	·	·	–	·	–	·	G	·	–	–	–	–	–	–	·	·	·	T	·	·	·	·	·
H33	T	·	G	C	·	A	T	·	A	T	·	·	·	·	·	·	–	·	–	·	G	·	–	–	–	–	–	–	·	·	·	T	·	·	·	·	·
H34	T	·	·	C	·	·	·	·	A	T	·	·	·	·	C	·	–	·	·	·	G	·	–	–	–	–	–	–	·	·	A	T	·	·	C	·	·
H35	·	·	·	·	·	·	·	·	·	C	·	·	·	·	·	·	–	·	·	·	·	·	–	–	–	–	–	–	·	·	·	·	·	·	·	·	·
H36	·	·	·	·	·	·	·	·	·	C	·	·	·	·	·	·	–	·	·	·	·	·	–	–	–	–	–	–	·	·	·	·	·	·	·	·	·
H37	·	·	·	·	·	·	·	·	·	C	·	·	·	·	·	·	–	·	·	·	·	·	–	–	–	–	–	–	·	·	·	·	·	·	·	·	·
H38	·	·	·	·	·	·	·	·	·	C	·	·	·	·	·	·	–	·	·	·	·	·	–	–	–	–	–	–	·	·	·	·	·	·	·	·	·
H39	T	T	G	C	·	A	T	·	A	C	·	·	·	·	·	·	·	·	–	·	G	·	–	–	–	–	–	–	·	·	·	T	·	·	·	·	·

Table S2. (continued).

Haplotype	Variable nucleotide site in alignment																																					
	accD			*accD-psaI* IGS																										*trnL* intron								
	5665	6369	6389	6548	6550	6556	6680	6778	6779	6796	6797	6798	6799	6800	6801	6947	6954	7087	7125	7126	7127	7128	7129	7130	7131	7132	7133	7149	7239	7324	7377	7424	7429	7442	7475	7476	7477	7478
H1	A	A	C	A	T	T	T	–	–	–	–	–	–	–	–	T	A	G	A	A	C	A	T	A	T	A	T	T	A	C	G	A	G	G	–	–	–	–
H2	·	·	·	·	·	·	·	–	–	–	–	–	–	–	–	·	·	·	·	·	·	·	·	·	·	·	·	·	·	·	·	·	·	·	–	–	–	–
H3	·	·	·	·	·	–	G	–	–	–	–	–	–	–	–	·	·	·	·	·	·	·	·	·	·	·	·	·	C	·	·	C	·	·	–	–	–	–
H4	·	·	T	·	·	–	G	–	–	–	–	–	–	–	–	·	·	·	·	·	·	·	·	·	·	·	·	·	C	·	·	C	·	·	–	–	–	–
H5	·	·	·	·	·	–	G	–	–	–	–	–	–	–	–	·	·	·	·	·	·	·	·	·	·	·	·	·	C	·	·	C	·	·	–	–	–	–
H6	·	·	·	·	·	–	G	–	–	–	–	–	–	–	–	·	·	·	·	·	·	·	·	·	·	·	·	·	C	·	·	C	·	·	–	–	–	–
H7	·	·	·	·	·	–	G	–	–	–	–	–	–	–	–	·	·	·	·	·	·	·	·	·	·	·	·	·	C	·	·	C	·	·	–	–	–	–
H8	·	C	·	·	·	·	G	T	–	–	–	–	–	–	–	·	·	·	·	·	·	·	·	·	·	·	·	C	·	·	·	C	·	·	–	–	–	–
H9	·	·	·	·	·	·	G	–	–	–	–	–	–	–	–	·	·	·	·	·	·	·	·	·	·	·	·	C	·	·	·	C	·	·	–	–	–	–
H10	·	C	·	·	·	·	G	T	–	–	–	–	–	–	–	·	·	·	·	·	·	·	·	·	·	·	·	C	·	·	·	C	·	·	–	–	–	–
H11	C	·	·	·	·	·	G	–	–	–	–	–	–	–	–	C	·	·	·	·	·	·	·	·	·	·	·	·	·	·	·	C	·	·	–	–	–	–
H12	C	·	·	·	·	·	G	T	–	–	–	–	–	–	–	C	·	·	·	·	·	·	·	·	·	·	·	·	·	·	·	C	·	·	–	–	–	–
H13	·	·	·	·	·	·	G	T	–	A	T	A	T	T	C	C	·	·	·	·	·	·	·	·	·	·	·	·	·	·	·	C	·	·	–	–	–	–
H14	·	·	·	·	·	·	G	T	–	–	–	–	–	–	–	·	·	·	·	·	·	·	·	·	·	·	·	·	·	·	·	C	·	·	–	–	–	–
H15	·	·	·	·	·	·	G	T	–	–	–	–	–	–	–	·	·	·	·	·	·	·	·	·	·	·	·	·	·	·	·	C	·	·	–	–	–	–
H16	·	·	·	·	·	·	G	T	–	–	–	–	–	–	–	·	·	·	·	·	·	·	·	·	·	·	·	·	·	·	·	C	·	·	–	–	–	–
H17	·	·	·	·	·	·	G	–	–	–	–	–	–	–	–	·	·	·	·	·	·	·	·	·	·	·	·	·	·	·	·	C	·	·	–	–	–	–
H18	·	·	·	·	·	·	G	–	–	–	–	–	–	–	–	·	·	·	·	·	·	·	·	·	·	·	·	·	·	·	·	C	·	A	–	–	–	–
H19	·	·	·	·	·	·	·	T	–	–	–	–	–	–	–	·	·	·	·	·	·	·	·	·	·	·	·	·	·	·	·	C	·	·	–	–	–	–
H20	·	·	·	·	·	·	·	–	–	–	–	–	–	–	–	·	·	A	·	·	·	·	·	·	·	·	·	·	·	·	·	C	T	·	–	–	–	–
H21	·	·	·	·	·	·	·	–	–	–	–	–	–	–	–	·	G	A	·	·	·	·	·	·	·	·	·	·	·	·	·	C	·	·	–	–	–	–
H22	·	·	·	·	·	·	·	–	–	–	–	–	–	–	–	·	·	A	·	·	·	·	·	·	·	·	·	·	·	·	·	C	T	·	–	–	–	–
H23	·	·	·	·	·	·	·	T	T	–	–	–	–	–	–	·	·	·	·	·	·	·	·	·	·	·	·	·	·	·	·	C	T	·	–	–	–	–
H24	·	·	·	·	·	·	·	–	–	–	–	–	–	–	–	·	·	A	·	·	·	·	·	·	·	·	·	·	·	·	·	C	·	·	–	–	–	–
H25	·	·	·	·	·	·	·	–	–	–	–	–	–	–	–	·	·	A	·	·	·	·	·	·	·	·	·	·	·	·	·	C	T	·	–	–	–	–
H26	·	·	·	·	·	·	·	–	–	–	–	–	–	–	–	·	·	·	·	·	·	·	·	·	·	·	·	·	·	·	·	C	·	·	–	–	–	–
H27	·	·	·	·	·	·	·	–	–	–	–	–	–	–	–	·	·	·	–	–	–	–	–	–	–	–	–	·	·	·	·	C	·	·	A	A	A	T
H28	·	·	·	·	·	·	·	–	–	–	–	–	–	–	–	·	·	·	·	·	·	·	·	·	·	·	·	·	·	·	·	C	·	·	–	–	–	–
H29	·	·	·	·	·	·	·	–	–	–	–	–	–	–	–	·	·	·	·	·	·	·	·	·	·	·	·	·	·	A	·	C	·	·	–	–	–	–
H30	·	·	·	·	·	·	·	–	–	–	–	–	–	–	–	·	·	·	–	–	–	–	–	–	–	–	–	·	·	·	·	C	·	·	A	A	A	T
H31	·	·	·	·	·	·	·	–	–	–	–	–	–	–	–	·	·	·	–	–	–	–	–	–	–	–	–	·	·	·	·	C	·	·	A	A	A	T
H32	·	·	·	·	·	·	·	–	–	–	–	–	–	–	–	·	·	·	–	–	–	–	–	–	–	–	–	·	·	·	·	C	·	·	A	A	A	T
H33	·	·	·	·	·	·	·	–	–	–	–	–	–	–	–	·	·	·	–	–	–	–	–	–	–	–	–	·	·	·	·	C	·	·	A	A	A	T
H34	·	·	·	·	·	·	·	–	–	–	–	–	–	–	–	·	G	A	·	·	·	·	·	·	·	·	·	·	·	·	·	C	·	·	–	–	–	–
H35	·	·	·	C	·	·	·	–	–	–	–	–	–	–	–	·	·	·	·	·	·	·	·	·	·	·	·	·	·	·	A	C	·	·	–	–	–	–
H36	·	·	·	C	·	·	·	–	–	–	–	–	–	–	–	·	·	·	·	·	·	·	·	·	·	·	·	·	·	·	·	C	·	·	–	–	–	–
H37	·	·	·	C	G	·	·	–	–	–	–	–	–	–	–	·	·	·	·	·	·	·	·	·	·	·	·	·	·	·	·	C	·	·	–	–	–	–
H38	·	·	·	C	·	·	·	–	–	–	–	–	–	–	–	·	·	·	·	·	·	·	·	·	·	·	·	·	·	·	·	C	·	·	–	–	–	–
H39	·	·	·	·	·	·	·	–	–	–	–	–	–	–	–	·	·	·	·	·	·	·	·	·	·	·	·	·	·	·	·	C	·	·	–	–	–	–

Table S2. (continued).

Haplotype	Variable nucleotide site in alignment																															
	trnL intron																										*trnL-trnF* IGS					
	7479	7480	7483	7494	7495	7496	7497	7516	7517	7518	7519	7520	7526	7527	7528	7529	7530	7531	7532	7533	7534	7536	7537	7558	7687	7725	8039	8048	8098	8106	8170	8175
H1	–	–	A	–	–	A	T	A	T	T	T	A	T	A	T	A	T	A	T	A	T	–	–	A	A	A	A	T	G	C	A	A
H2	–	–	·	–	–	·	·	·	·	·	·	·	·	·	·	·	·	·	·	·	·	–	–	·	·	·	·	·	·	·	·	·
H3	–	–	·	–	–	·	·	–	–	–	–	–	·	·	·	·	·	·	·	·	·	–	–	·	·	G	·	·	·	·	G	·
H4	–	–	·	–	–	·	·	–	–	–	–	–	·	·	·	·	·	·	·	·	·	–	–	·	·	G	·	·	·	·	G	·
H5	–	–	·	–	–	·	·	–	–	–	–	–	·	·	·	·	·	·	·	·	·	–	–	·	·	G	·	·	·	·	G	·
H6	–	–	·	–	–	·	·	–	–	–	–	–	·	·	·	·	·	·	·	·	·	–	–	·	·	G	·	·	·	G	G	·
H7	–	–	·	–	–	·	·	–	–	–	–	–	·	·	·	·	·	·	·	·	·	–	–	·	·	G	·	·	·	·	G	·
H8	–	–	·	–	–	·	·	–	–	–	–	–	–	·	·	·	·	·	·	·	·	–	–	T	C	G	C	·	·	·	·	G
H9	–	–	·	A	T	·	·	–	–	–	–	–	–	–	–	–	–	–	–	–	–	–	A	T	C	G	C	·	·	·	·	G
H10	–	–	·	–	–	·	·	–	–	–	–	–	–	·	·	·	·	·	·	·	·	–	–	T	C	G	C	·	·	·	·	G
H11	–	–	·	–	–	·	·	–	–	–	–	–	·	·	·	·	·	·	·	·	·	–	–	·	·	G	·	·	·	·	G	·
H12	–	–	·	–	–	·	·	–	–	–	–	–	·	·	·	·	·	·	·	·	·	–	A	·	·	G	·	·	·	·	G	·
H13	–	–	·	–	–	·	·	–	–	–	–	–	·	·	·	·	·	·	·	·	·	–	–	·	·	G	·	·	·	·	G	·
H14	–	–	·	–	–	·	·	–	–	–	–	–	·	·	·	·	·	·	·	·	·	–	–	·	·	G	·	·	·	·	G	·
H15	–	–	·	–	–	·	·	–	–	–	–	–	·	·	·	·	·	·	·	·	·	–	–	·	·	G	·	·	·	·	G	·
H16	–	–	·	–	–	·	·	–	–	–	–	–	·	·	·	·	·	·	·	·	·	–	–	·	·	G	·	·	·	·	G	·
H17	–	–	·	–	–	·	·	–	–	–	–	–	·	·	·	·	·	·	·	·	·	–	–	·	·	G	·	·	·	·	G	·
H18	–	–	·	–	–	·	·	–	–	–	–	–	·	·	·	·	·	·	·	·	·	–	–	·	·	G	·	·	·	·	G	·
H19	–	–	·	A	T	·	·	–	–	–	–	–	·	·	·	·	·	·	·	·	·	T	A	·	·	G	·	·	·	·	·	·
H20	–	–	·	–	–	–	–	–	–	–	–	–	·	·	·	·	·	·	·	·	·	–	–	·	·	G	·	·	A	·	·	·
H21	–	–	·	–	–	–	–	–	–	–	–	–	·	·	·	·	·	·	·	·	·	–	–	·	·	G	·	·	A	·	·	·
H22	–	–	·	–	–	–	–	–	–	–	–	–	·	·	·	·	·	·	·	·	·	–	–	·	·	G	·	·	A	·	·	·
H23	–	–	·	–	–	–	–	–	–	–	–	–	·	·	·	·	·	·	·	·	·	–	–	·	·	G	·	·	A	·	·	·
H24	–	–	·	–	–	–	–	–	–	–	–	–	·	·	·	·	·	·	·	·	·	–	–	·	·	G	·	·	A	·	·	·
H25	–	–	·	–	–	–	–	–	–	–	–	–	·	·	·	·	·	·	·	·	·	–	–	·	·	G	·	·	A	·	·	·
H26	–	–	·	A	T	·	·	–	–	–	–	–	·	·	·	·	·	·	·	·	·	–	–	·	·	G	·	·	·	·	·	·
H27	A	T	·	A	T	·	·	–	–	–	–	–	·	·	·	·	·	·	·	·	·	–	–	·	·	G	·	·	·	·	·	·
H28	–	–	·	A	T	·	·	–	–	–	–	–	·	·	·	·	·	·	·	·	·	–	–	·	·	G	·	·	·	·	·	·
H29	–	–	·	A	T	·	·	–	–	–	–	–	·	·	·	·	·	·	·	·	·	–	–	·	·	G	·	·	·	·	·	·
H30	A	T	·	A	T	·	·	–	–	–	–	–	·	·	·	·	·	·	·	·	·	–	–	·	·	G	·	·	·	·	·	·
H31	A	T	·	A	T	·	·	–	–	–	–	–	·	·	·	·	·	·	·	·	·	–	–	·	·	G	·	·	·	·	·	·
H32	A	T	·	A	T	·	·	–	–	–	–	–	·	·	·	·	·	·	·	·	·	–	–	·	·	G	·	·	·	·	·	·
H33	A	T	·	A	T	·	·	–	–	–	–	–	·	·	·	·	·	·	·	·	·	–	–	·	·	G	·	·	·	·	·	·
H34	–	–	·	–	–	–	–	–	–	–	–	–	·	·	·	·	·	·	·	·	·	–	–	·	·	G	·	·	A	·	·	·
H35	–	–	·	–	–	·	·	–	–	–	–	–	·	·	·	·	·	·	·	·	·	–	–	·	·	·	·	·	·	·	·	·
H36	–	–	·	–	–	·	·	–	–	–	–	–	·	·	·	·	·	·	·	·	·	–	–	·	·	·	·	·	·	·	·	·
H37	–	–	·	–	–	·	·	–	–	–	–	–	·	·	·	·	·	·	·	·	·	T	A	·	·	·	·	·	·	·	·	·
H38	–	–	·	–	–	·	·	–	–	–	–	–	·	·	·	·	·	·	·	·	·	–	–	·	·	·	·	·	·	·	·	·
H39	–	–	C	A	T	·	·	–	–	–	–	–	·	·	·	·	·	·	·	·	·	–	–	·	·	G	·	G	·	·	·	·

Appendix S1

Methods for nDNA analyses: We examined the nDNA *LEAFY* region for a subset of taxa for which we had sufficient quality and quantity of DNA. The second intron of the *LEAFY* region was amplified by PCR using primers PLFY-F3 [1] and LFY2358R [2]. PCR cycling conditions consisted of 3 min pre-denaturation at 95 °C, 1 min denaturation at 95 °C, 40 sec annealing at 52°C, 1 min extension at 72°C, and 7 min final extension at 72°C. Due to the presence of polymorphic nucleotide positions in direct sequences, PCR products of the nDNA *LEAFY* region for 20 accessions were cloned using the pGEM-T easy vector system I (Promega, Madison, U. S. A.) following the manufacturer's instructions. Five to 10 colonies per plate were randomly selected and sequenced. Sequence alignment, phylogenetic analyses (Bayesian Inference), and all other tasks were conducted identically to those for cpDNA analyses as outlined in the Methods section. Summary statistics for the nDNA dataset are presented in Table SA1.

1. Kim, S.-T., Sultan, S. E. & Donoghue, M. J. Allopolyploid speciation in Persicaria (Polygonaceae): insights from a low-copy nuclear region. *Proc. Natl. Acad. Sci.* (2008).
2. Schuster, T. M., Wilson, K. L. & Kron, K. A. Phylogenetic relationships of Muehlenbeckia, Fallopia, and Reynoutria (Polygonaceae) investigated with chloroplast and nuclear sequence data. *Int. J. Plant Sci.* **172,** 1053–1066 (2011).

Table SA1. Statistics for the nDNA data sets used in this study. Three outgroup taxa were included in the calculation of these statistics, with the exception of p-distance.

	LEAFY
Sequence length (bp)	272-755
Aligned length (bp)	907
G+C ratio (%)	26.9 - 29.7
No. of variable characters (%)	147 (16.2)
No. of parsimony informative characters (%)	91 (10.0)
p-distance (mean)	0 - 0.0109 (0.0051)
MP tree length	183
No. of MP trees	5559
Consistency index (CI)	0.847
Retention index (RI)	0.953
Optimal model of sequence evolution	GTR+Γ

Table SA2. Variable nucleotide sites for nDNA *LEAFY* haplotypes recovered from 99 a ccessions of *F. schalienesis* and closely related taxa (outgroup taxa excluded). Accession numbers refer to those in Table S1.

		Variable nucleotide site in alignment														
		0	0	0	0	0	0	0	0	0	0	0	0	0	0	0
Haplo		1	1	2	3	4	5	5	5	5	6	6	6	6	6	6
-type	Accession	0	6	1	4	6	0	1	2	3	1	2	3	4	5	6
N1	S_ul02-06, 08, 11-17, 19, 20, 22, 23, dk01, sk01, 03, 05, 06, 08, 09, 11, us01, hk01-05, 07, 08, 10, hn01-04, 08, 10, 13, 17, 19-21, 23	T	A	T	G	G	-	-	-	-	T	G	A	T	T	G
N2	S_sk04, 08, 11	.	.	.	.	.	-	-	-	-	.	.	.	.	.	.
N3	S_hk06, hn12, J_hn03, 05	.	.	.	.	.	-	-	-	-	.	.	.	.	.	.
N4	J_hn04	.	.	.	.	A	A	T	A	T	.	.	.	.	.	.
N5	S_ul21, sk02, 03, 07, 10, hk01, 09	.	.	.	.	.	-	-	-	-	.	.	.	.	.	.
N6	S_hk03, 05	.	.	.	.	.	-	-	-	-	.	.	.	.	.	.
N7	S_hk04	.	.	.	.	.	-	-	-	-	.	.	.	.	.	.
N8	S_hn01, 04, 06, 20, 22	.	.	.	.	.	-	-	-	-	.	.	.	.	.	.
N9	S_hn05	.	.	.	.	.	-	-	-	-	.	.	.	.	.	.
N10	S_hn06, 07	.	.	.	.	.	-	-	-	-	.	.	.	.	.	.
N11	S_hn07	.	.	.	.	.	-	-	-	-	.	.	.	.	.	.
N12	S_hn09	.	.	.	.	.	-	-	-	-	.	.	.	.	.	.
N13	S_hn11, 14-16, uk01	.	.	.	.	.	-	-	-	-	.	.	.	.	.	.
N14	S_hn12	.	.	.	.	.	-	-	-	-	.	.	.	.	.	.
N15	S_uk01	.	.	.	.	.	-	-	-	-	.	.	.	.	.	.
N16	S_ul01, 07, 18	.	.	.	.	.	-	-	-	-	.	.	.	.	.	.
N17	S_ul21	.	.	.	.	.	-	-	-	-	.	.	.	.	.	.
N18	S_ul22	.	.	.	.	.	-	-	-	-	.	.	.	.	.	.
N19	J_hn01	.	.	.	A	A	A	T	A	T	-	-	-	-	-	-
N20	J_hn02	.	.	.	.	A	A	T	A	T	.	.	.	.	.	.
N21	J_hn03, 05	.	.	.	.	A	A	T	A	T	.	C	.	.	.	T
N22	J_hn04	.	.	.	.	.	-	-	-	-	.	.	.	.	.	.
N23	J_hn04	.	.	.	.	A	A	T	A	T	-	-	-	-	-	-
N24	J_hn05	.	.	.	.	A	A	T	A	T	.	C	.	.	.	T
N25	J_hn06	.	.	.	.	A	A	T	A	T	.	.	.	.	.	.
N26	J_hn09	.	.	.	.	A	A	T	A	T	.	C	.	.	.	T
N27	J_hn10	.	.	A	.	A	A	T	A	T	.	C	.	.	.	T
N28	J_kr01, 03, 04, 07, 08	.	G	.	.	A	A	T	A	T	.	C	.	.	.	T
N29	J_kr02, 10-13	.	.	.	.	A	A	T	A	T	.	.	.	.	.	.
N30	J_kr05, 09, ky05	.	.	.	.	A	A	T	A	T	.	C	.	.	.	T
N31	J_kr06	.	.	.	.	A	A	T	A	T	.	C	.	.	.	T
N32	J_kr06	.	.	.	.	A	A	T	A	T	.	C	.	.	.	T
N33	J_kr13	.	G	.	.	A	A	T	A	T	.	C	.	.	.	T
N34	J_ky01, 03	.	.	.	.	A	A	T	A	T	.	C	.	.	.	T
N35	J_ky02	.	.	.	.	A	A	T	A	T	.	C	.	.	.	T
N36	J_ky04	.	.	.	.	A	A	T	A	T	.	C	.	.	.	T
N37	J_ky06	.	.	.	.	A	A	T	A	T	.	C	.	.	.	T
N38	J_rs01	.	.	.	.	A	A	T	A	T	.	C	.	.	.	T
N39	J_rs01	.	.	.	.	A	A	T	A	T	.	C	.	.	.	T
N40	F_kr01-03	C	.	.	.	A	-	-	-	-	.	.	.	.	.	.

Table SA2. (continued).

Haplo-type	Variable nucleotide site in alignment																															
	067	075	076	077	078	079	080	081	082	083	084	085	086	087	089	090	091	092	093	094	095	096	097	098	099	100	101	102	103	110	111	112	113
N1	T	-	-	-	-	A	T	G	T	A	G	T	T	T	G	A	T	C	A	T	A	T	A	G	T	A	A	T	T	A	T	A	G
N2	.	-	-	-	-	.	.	.	.	.	.	.	.	.	.	.	.	.	.	.	.	.	.	.	.	.	.	.	.	.	.	.	.
N3	.	-	-	-	-	.	.	.	.	.	.	.	.	.	.	.	.	.	.	.	.	.	.	.	.	.	.	.	.	.	.	.	.
N4	.	A	T	G	T	.	C	.	.	.	.	.	.	.	.	.	.	.	.	.	.	.	.	.	.	.	.	.	.	.	.	.	.
N5	.	-	-	-	-	.	.	.	.	.	.	.	.	.	.	.	.	.	.	.	.	.	.	.	.	.	.	.	.	.	.	.	.
N6	.	-	-	-	-	.	.	.	.	.	.	.	.	.	.	.	.	.	.	.	.	.	.	.	.	.	.	.	.	.	.	.	.
N7	.	-	-	-	-	.	.	.	.	.	.	.	.	.	.	.	.	.	.	.	.	.	.	.	.	.	.	.	.	.	.	.	.
N8	.	-	-	-	-	.	.	.	.	.	.	.	.	.	.	.	.	.	.	.	.	.	.	.	.	.	.	.	.	.	.	.	.
N9	.	-	-	-	-	.	.	.	.	.	.	.	.	.	.	.	.	.	.	.	.	.	.	.	.	.	.	.	.	.	.	.	.
N10	.	-	-	-	-	.	.	.	.	.	.	.	.	.	.	.	.	.	.	.	.	.	.	.	.	.	.	.	.	.	.	.	.
N11	.	-	-	-	-	.	.	.	.	.	.	.	.	.	.	.	.	.	.	.	.	.	.	.	.	.	.	.	.	.	.	.	.
N12	.	-	-	-	-	.	.	.	.	.	.	.	.	.	.	.	.	.	.	.	.	.	.	.	.	.	.	.	.	.	.	.	.
N13	.	-	-	-	-	.	.	.	.	.	.	.	.	.	.	.	.	.	.	.	.	.	.	.	.	.	.	.	.	.	.	.	.
N14	.	-	-	-	-	.	.	.	.	.	.	.	.	.	.	.	.	.	.	.	.	.	.	.	.	.	.	.	.	.	.	.	.
N15	.	-	-	-	-	.	.	.	.	.	.	.	.	.	.	.	.	.	.	.	.	.	.	.	.	.	.	.	.	.	.	.	.
N16	.	-	-	-	-	.	.	.	.	.	.	.	.	.	.	.	.	.	.	.	.	.	.	.	.	.	.	.	.	.	.	.	.
N17	.	-	-	-	-	.	.	.	.	.	.	.	.	.	.	.	.	.	.	.	.	.	.	.	.	.	.	.	.	.	.	.	.
N18	.	-	-	-	-	.	.	.	.	.	.	.	.	.	.	.	.	.	.	.	.	.	.	.	.	.	.	.	.	.	.	.	.
N19	-	-	-	-	-	-	-	-	-	-	-	-	-	-	-	-	-	-	-	-	-	-	-	-	-	-	-	-	-	-	-	-	-
N20	.	A	T	G	T	.	C	.	.	.	.	.	.	.	.	.	.	.	.	.	.	.	.	.	.	.	.	.	.	.	.	.	.
N21	.	-	-	-	-	.	.	.	.	.	.	.	.	.	.	.	.	.	.	.	.	.	.	.	.	.	.	.	.	.	.	.	.
N22	.	-	-	-	-	.	.	.	.	.	.	.	.	.	.	.	.	.	.	.	.	.	.	.	.	.	.	.	.	.	.	.	.
N23	-	-	-	-	-	-	-	-	-	-	-	-	-	-	-	-	-	-	-	-	-	-	-	-	-	-	-	-	-	-	-	-	-
N24	.	-	-	-	-	.	.	.	.	.	.	.	.	.	.	.	.	.	.	.	.	.	.	.	.	.	.	.	.	.	.	.	.
N25	.	-	-	-	-	.	.	.	.	.	.	.	.	.	.	.	.	.	.	.	.	.	.	.	.	.	.	.	.	.	.	.	.
N26	.	-	-	-	-	.	.	.	.	.	.	.	.	.	.	.	.	.	.	.	.	.	.	.	.	.	.	.	.	.	.	.	.
N27	.	-	-	-	-	.	.	.	.	.	.	.	.	.	.	.	.	.	.	.	.	.	.	.	.	.	.	.	.	.	.	.	.
N28	.	-	-	-	-	.	.	.	.	.	.	.	.	.	.	.	.	.	.	.	.	.	.	.	.	.	.	.	.	.	.	.	.
N29	.	-	-	-	-	.	.	.	.	.	.	.	.	.	.	.	.	.	.	.	.	.	.	.	.	.	.	.	.	.	.	.	.
N30	.	-	-	-	-	.	.	.	.	.	.	.	.	.	.	.	.	.	.	.	.	.	.	.	.	.	.	.	.	.	.	.	.
N31	.	-	-	-	-	.	.	.	.	.	.	.	.	.	.	.	.	.	.	.	.	.	.	.	.	.	.	.	.	.	.	.	.
N32	.	-	-	-	-	.	.	.	.	.	.	.	.	.	.	.	.	.	.	.	.	.	.	.	.	.	.	.	.	.	.	.	.
N33	.	-	-	-	-	.	.	.	.	.	.	.	.	.	.	.	.	.	.	.	.	.	.	.	.	.	.	.	.	.	.	.	.
N34	.	-	-	-	-	.	.	.	.	.	.	.	.	.	.	.	.	.	.	.	.	.	.	.	.	.	.	.	.	.	.	.	.
N35	.	-	-	-	-	.	.	.	.	.	.	.	.	.	.	.	.	.	.	.	.	.	.	.	.	.	.	.	.	.	.	.	.
N36	.	-	-	-	-	.	.	.	.	.	.	.	.	.	.	.	.	.	.	.	.	.	.	.	.	.	.	.	.	.	.	.	.
N37	.	-	-	-	-	.	.	.	.	.	.	.	.	.	.	.	.	.	.	.	.	.	.	.	.	.	.	.	.	.	.	.	.
N38	.	-	-	-	-	.	.	.	.	.	.	.	.	.	.	.	.	.	.	.	.	.	.	.	.	.	.	.	.	.	.	.	.
N39	.	-	-	-	-	.	.	.	.	.	.	.	.	.	.	.	.	.	.	.	.	.	.	.	.	.	.	.	.	.	.	.	.
N40	.	-	-	-	-	.	.	.	.	.	.	.	.	.	.	.	.	.	.	.	.	.	.	.	.	.	.	.	.	.	.	.	.

Table SA2. (continued).

Haplo-type	Variable nucleotide site in alignment																														
	114	115	116	117	118	119	120	121	122	123	124	125	126	127	128	129	130	131	132	133	134	135	136	137	138	139	140	141	142	143	144	145
N1	C	T	A	G	T	T	A	G	C	T	A	G	G	-	-	-	-	-	-	-	-	-	-	-	-	-	-	-	-	-	-	-
N2	.	.	.	.	.	.	.	.	.	.	.	.	.	-	-	-	-	-	-	-	-	-	-	-	-	-	-	-	-	-	-	-
N3	.	.	.	.	.	.	.	.	.	.	.	.	.	-	-	-	-	-	-	-	-	-	-	-	-	-	-	-	-	-	-	-
N4	.	C	.	.	.	.	.	.	.	.	.	.	.	-	-	-	-	T	A	A	A	G	T	G	A	G	A	G	A	C	G	A
N5	.	.	.	.	.	.	.	.	.	.	.	.	.	-	-	-	-	-	-	-	-	-	-	-	-	-	-	-	-	-	-	-
N6	.	.	.	.	.	.	.	.	.	.	.	.	.	-	-	-	-	-	-	-	-	-	-	-	-	-	-	-	-	-	-	-
N7	.	.	.	.	.	.	.	.	.	.	.	.	.	-	-	-	-	-	-	-	-	-	-	-	-	-	-	-	-	-	-	-
N8	.	.	.	.	.	.	.	.	.	.	.	.	.	-	-	-	-	-	-	-	-	-	-	-	-	-	-	-	-	-	-	-
N9	.	.	.	.	.	.	.	.	.	.	.	.	.	-	-	-	-	-	-	-	-	-	-	-	-	-	-	-	-	-	-	-
N10	.	.	.	.	.	.	.	.	.	.	.	.	.	-	-	-	-	-	-	-	-	-	-	-	-	-	-	-	-	-	-	-
N11	.	.	.	.	.	.	.	.	.	.	.	.	.	-	-	-	-	-	-	-	-	-	-	-	-	-	-	-	-	-	-	-
N12	.	.	.	.	.	.	.	.	.	.	.	.	.	-	-	-	-	-	-	-	-	-	-	-	-	-	-	-	-	-	-	-
N13	.	.	.	.	.	.	.	.	.	.	.	-	-	-	-	-	-	-	-	-	-	-	-	-	-	-	-	-	-	-	-	-
N14	.	.	.	.	.	.	.	.	.	.	.	.	.	-	-	-	-	-	-	-	-	-	-	-	-	-	-	-	-	-	-	-
N15	.	.	.	.	.	.	.	.	.	.	.	-	-	-	-	-	-	-	-	-	-	-	-	-	-	-	-	-	-	-	-	-
N16	.	.	.	.	.	.	.	.	.	.	.	.	.	-	-	-	-	-	-	-	-	-	-	-	-	-	-	-	-	-	-	-
N17	.	.	.	.	.	.	.	.	.	.	.	.	.	-	-	-	-	-	-	-	-	-	-	-	-	-	-	-	-	-	-	-
N18	.	.	.	.	.	.	.	.	.	.	.	.	.	-	-	-	-	-	-	-	-	-	-	-	-	-	-	-	-	-	-	-
N19	-	-	-	-	-	-	-	-	-	-	-	-	-	-	-	-	-	-	-	-	-	-	-	-	-	-	-	-	-	-	-	-
N20	.	C	.	.	.	.	.	.	.	.	.	.	.	-	-	-	-	T	A	A	A	G	T	G	A	G	A	G	A	C	G	A
N21	T	.	.	.	.	.	.	.	.	.	.	.	.	-	-	-	-	T	A	A	A	G	T	G	A	G	A	G	A	C	G	A
N22	.	.	.	.	.	.	.	.	.	.	.	.	.	-	-	-	-	-	-	-	-	-	-	-	-	-	-	-	-	-	-	-
N23	-	-	-	-	-	-	-	-	-	-	-	-	-	-	-	-	-	-	-	-	-	-	-	-	-	-	-	-	-	-	-	-
N24	.	.	.	.	.	.	.	.	.	.	.	.	.	T	A	T	G	T	A	A	A	G	T	G	A	G	A	G	A	C	G	A
N25	.	C	.	.	.	.	.	.	.	.	.	.	.	-	-	-	-	T	A	A	A	G	T	G	A	G	A	G	A	C	G	A
N26	.	.	.	.	.	.	.	.	.	.	.	.	.	-	-	-	-	T	A	A	A	G	T	G	A	G	A	G	A	C	G	-
N27	.	.	.	.	.	.	.	.	.	.	.	.	.	T	A	C	G	T	A	A	A	G	T	G	A	G	A	G	A	C	G	A
N28	.	.	.	.	.	.	.	.	.	.	.	.	.	-	-	-	-	T	A	A	A	G	T	G	A	G	A	G	A	C	G	-
N29	.	C	.	.	.	.	.	.	.	.	.	.	.	-	-	-	-	T	A	A	A	G	T	G	A	G	A	G	A	C	G	A
N30	.	.	.	.	.	.	.	.	.	.	.	.	.	T	A	C	G	T	A	A	A	G	T	G	A	G	A	G	A	C	G	A
N31	.	.	.	.	.	.	.	.	.	.	.	.	.	T	A	C	G	T	A	A	A	G	T	G	A	G	A	G	A	C	G	A
N32	.	.	.	.	.	.	.	.	.	.	.	.	.	T	A	C	G	T	A	A	A	G	T	G	A	G	A	G	A	C	G	A
N33	.	.	.	.	.	.	.	.	.	.	.	.	.	-	-	-	-	T	A	A	A	G	T	G	A	G	A	G	A	C	G	-
N34	.	.	.	.	.	.	.	.	.	.	.	.	.	T	A	C	G	T	A	A	A	G	T	G	A	G	A	G	A	C	G	A
N35	.	.	.	.	.	.	.	.	.	.	.	.	.	T	A	C	G	T	A	A	A	G	T	G	A	G	A	G	A	C	G	A
N36	.	.	.	.	.	.	.	.	.	.	.	.	.	T	A	C	G	T	A	A	A	G	T	G	A	G	A	G	A	C	G	A
N37	.	C	.	.	.	.	.	.	.	.	.	.	.	-	-	-	-	T	A	A	A	G	T	G	A	G	A	G	A	C	G	A
N38	.	.	.	.	.	.	.	.	.	.	.	.	.	-	-	-	-	T	A	A	A	G	T	G	A	G	A	G	A	C	G	-
N39	.	.	.	.	.	.	.	.	.	.	.	.	.	-	-	-	-	T	A	A	A	G	T	G	A	G	A	G	A	C	G	-
N40	.	C	.	.	.	.	.	.	.	.	G	.	.	-	-	-	-	T	A	A	A	G	T	G	A	G	A	G	A	C	G	A

Table SA2. (continued).

	Variable nucleotide site in alignment																														
	1	1	1	1	1	1	1	1	1	1	1	1	1	1	1	1	1	1	1	1	1	1	1	1	1	1	1	1	1	1	1	1
Haplo	4	4	4	4	5	5	5	5	5	5	5	5	5	5	6	6	6	6	6	6	6	6	6	6	7	7	7	7	7	7	7	7
-type	6	7	8	9	0	1	2	3	4	5	6	7	8	9	0	1	2	3	4	5	6	7	8	9	0	1	2	3	4	5	6	7
N1	-	-	-	-	-	-	-	-	-	-	-	-	-	-	-	-	-	-	-	-	-	-	-	-	-	-	-	-	-	-	-	-
N2	-	-	-	-	-	-	-	-	-	-	-	-	-	-	-	-	-	-	-	-	-	-	-	-	-	-	-	-	-	-	-	-
N3	-	-	-	-	-	-	-	-	-	-	-	-	-	-	-	-	-	-	-	-	-	-	-	-	-	-	-	-	-	-	-	-
N4	C	G	T	A	T	A	A	A	T	G	A	C	A	A	C	T	T	T	G	G	A	T	T	C	A	T	A	A	T	T	T	T
N5	-	-	-	-	-	-	-	-	-	-	-	-	-	-	-	-	-	-	-	-	-	-	-	-	-	-	-	-	-	-	-	-
N6	-	-	-	-	-	-	-	-	-	-	-	-	-	-	-	-	-	-	-	-	-	-	-	-	-	-	-	-	-	-	-	-
N7	-	-	-	-	-	-	-	-	-	-	-	-	-	-	-	-	-	-	-	-	-	-	-	-	-	-	-	-	-	-	-	-
N8	-	-	-	-	-	-	-	-	-	-	-	-	-	-	-	-	-	-	-	-	-	-	-	-	-	-	-	-	-	-	-	-
N9	-	-	-	-	-	-	-	-	-	-	-	-	-	-	-	-	-	-	-	-	-	-	-	-	-	-	-	-	-	-	-	-
N10	-	-	-	-	-	-	-	-	-	-	-	-	-	-	-	-	-	-	-	-	-	-	-	-	-	-	-	-	-	-	-	-
N11	-	-	-	-	-	-	-	-	-	-	-	-	-	-	-	-	-	-	-	-	-	-	-	-	-	-	-	-	-	-	-	-
N12	-	-	-	-	-	-	-	-	-	-	-	-	-	-	-	-	-	-	-	-	-	-	-	-	-	-	-	-	-	-	-	-
N13	-	-	-	-	-	-	-	-	-	-	-	-	-	-	-	-	-	-	-	-	-	-	-	-	-	-	-	-	-	-	-	-
N14	-	-	-	-	-	-	-	-	-	-	-	-	-	-	-	-	-	-	-	-	-	-	-	-	-	-	-	-	-	-	-	-
N15	-	-	-	-	-	-	-	-	-	-	-	-	-	-	-	-	-	-	-	-	-	-	-	-	-	-	-	-	-	-	-	-
N16	-	-	-	-	-	-	-	-	-	-	-	-	-	-	-	-	-	-	-	-	-	-	-	-	-	-	-	-	-	-	-	-
N17	-	-	-	-	-	-	-	-	-	-	-	-	-	-	-	-	-	-	-	-	-	-	-	-	-	-	-	-	-	-	-	-
N18	-	-	-	-	-	-	-	-	-	-	-	-	-	-	-	-	-	-	-	-	-	-	-	-	-	-	-	-	-	-	-	-
N19	-	-	-	-	-	-	-	-	-	-	-	-	-	-	-	-	-	-	-	-	-	-	-	-	-	-	-	-	-	-	-	-
N20	C	G	T	A	T	A	A	A	T	G	A	C	A	A	C	T	T	T	G	G	A	T	T	C	A	T	A	A	T	T	T	T
N21	C	G	T	A	T	A	A	A	T	G	A	C	A	A	C	T	T	T	G	G	A	T	T	C	A	T	A	A	T	T	T	T
N22	-	-	-	-	-	-	-	-	-	-	-	-	-	-	-	-	-	-	-	-	-	-	-	-	-	-	-	-	-	-	-	-
N23	-	-	-	-	-	-	-	-	-	-	-	-	-	-	-	-	-	-	-	-	-	-	-	-	-	-	-	-	-	-	-	-
N24	C	G	T	A	T	A	A	A	T	G	A	C	A	A	C	T	T	T	G	G	A	T	T	C	A	T	A	A	T	T	T	T
N25	C	G	T	A	T	A	A	A	T	G	A	C	A	A	C	T	T	T	G	G	A	T	T	C	A	T	A	A	T	T	T	T
N26	-	-	T	A	T	A	A	A	T	G	A	C	A	A	C	T	T	T	C	G	A	T	T	C	A	T	A	A	T	T	T	T
N27	C	G	T	A	T	A	A	A	T	G	A	C	A	A	C	T	T	T	C	G	A	T	T	C	A	T	A	A	T	T	T	T
N28	-	-	T	A	T	A	A	A	T	G	A	C	A	A	C	T	T	T	C	G	A	T	T	C	A	T	A	A	T	T	T	T
N29	C	G	T	A	T	A	A	A	T	G	A	C	A	A	C	T	T	T	G	G	A	T	T	C	A	T	A	A	T	T	T	-
N30	C	G	T	A	T	A	A	A	T	G	A	C	A	A	C	T	T	T	C	G	A	T	T	C	A	T	A	A	T	T	T	T
N31	C	G	T	A	T	A	A	A	T	G	A	C	A	A	C	T	T	T	G	G	A	T	T	C	A	T	A	A	T	T	T	T
N32	C	G	T	A	T	A	A	A	T	G	A	C	A	A	C	T	T	T	G	G	A	T	T	C	A	T	A	A	T	T	T	T
N33	-	-	T	A	T	A	A	A	T	G	A	C	A	A	C	T	T	T	C	G	A	T	T	C	A	T	A	A	T	T	T	T
N34	C	G	T	A	T	A	A	A	T	G	A	C	A	A	C	T	T	T	C	G	A	T	T	C	A	T	A	A	T	T	T	T
N35	C	G	T	A	T	A	A	A	T	G	A	C	A	A	C	T	T	T	C	G	A	T	T	C	A	T	A	A	T	T	T	T
N36	C	G	T	A	T	A	A	A	T	G	A	C	A	A	C	T	T	T	C	G	A	T	T	C	A	T	A	A	T	T	T	T
N37	C	G	T	A	T	A	A	A	T	G	A	C	A	A	C	T	T	T	G	G	A	T	T	C	A	T	A	A	T	T	T	T
N38	-	-	T	A	T	A	A	A	T	G	A	C	A	A	C	T	T	T	C	G	A	T	T	C	A	T	A	A	T	T	T	T
N39	-	-	T	A	T	A	A	A	T	G	A	C	A	A	C	T	T	T	C	G	A	T	T	C	A	T	A	A	T	T	T	T
N40	C	G	T	A	T	A	A	A	T	G	A	C	A	C	C	T	T	T	G	G	A	T	T	C	A	T	A	A	T	T	T	T

Table SA2. (continued).

Haplo-type	Variable nucleotide site in alignment																														
	178	179	180	181	182	183	184	185	186	187	188	189	190	191	192	193	194	195	196	197	198	199	200	201	202	203	204	205	206	207	208	209
N1	-	-	-	-	-	-	-	-	-	-	-	-	-	-	-	-	-	-	-	-	-	-	-	-	-	-	-	-	-	-	-	-
N2	-	-	-	-	-	-	-	-	-	-	-	-	-	-	-	-	-	-	-	-	-	-	-	-	-	-	-	-	-	-	-	-
N3	-	-	-	-	-	-	-	-	-	-	-	-	-	-	-	-	-	-	-	-	-	-	-	-	-	-	-	-	-	-	-	-
N4	G	T	-	G	G	G	G	G	C	A	A	-	-	-	-	-	T	C	T	T	T	A	A	A	T	C	T	A	G	C	T	A
N5	-	-	-	-	-	-	-	-	-	-	-	-	-	-	-	-	-	-	-	-	-	-	-	-	-	-	-	-	-	-	-	-
N6	-	-	-	-	-	-	-	-	-	-	-	-	-	-	-	-	-	-	-	-	-	-	-	-	-	-	-	-	-	-	-	-
N7	-	-	-	-	-	-	-	-	-	-	-	-	-	-	-	-	-	-	-	-	-	-	-	-	-	-	-	-	-	-	-	-
N8	-	-	-	-	-	-	-	-	-	-	-	-	-	-	-	-	-	-	-	-	-	-	-	-	-	-	-	-	-	-	-	-
N9	-	-	-	-	-	-	-	-	-	-	-	-	-	-	-	-	-	-	-	-	-	-	-	-	-	-	-	-	-	-	-	-
N10	-	-	-	-	-	-	-	-	-	-	-	-	-	-	-	-	-	-	-	-	-	-	-	-	-	-	-	-	-	-	-	-
N11	-	-	-	-	-	-	-	-	-	-	-	-	-	-	-	-	-	-	-	-	-	-	-	-	-	-	-	-	-	-	-	-
N12	-	-	-	-	-	-	-	-	-	-	-	-	-	-	-	-	-	-	-	-	-	-	-	-	-	-	-	-	-	-	-	-
N13	-	-	-	-	-	-	-	-	-	-	-	-	-	-	-	-	-	-	-	-	-	-	-	-	-	-	-	-	-	-	-	-
N14	-	-	-	-	-	-	-	-	-	-	-	-	-	-	-	-	-	-	-	-	-	-	-	-	-	-	-	-	-	-	-	-
N15	-	-	-	-	-	-	-	-	-	-	-	-	-	-	-	-	-	-	-	-	-	-	-	-	-	-	-	-	-	-	-	-
N16	-	-	-	-	-	-	-	-	-	-	-	-	-	-	-	-	-	-	-	-	-	-	-	-	-	-	-	-	-	-	-	-
N17	-	-	-	-	-	-	-	-	-	-	-	-	-	-	-	-	-	-	-	-	-	-	-	-	-	-	-	-	-	-	-	-
N18	-	-	-	-	-	-	-	-	-	-	-	-	-	-	-	-	-	-	-	-	-	-	-	-	-	-	-	-	-	-	-	-
N19	-	-	-	-	-	-	-	-	-	-	-	-	-	-	-	-	-	-	-	-	-	-	-	-	-	-	-	-	-	-	-	-
N20	G	T	-	G	G	G	G	G	C	A	A	-	-	-	-	-	T	C	T	T	T	A	A	A	T	C	T	A	G	C	T	A
N21	G	T	-	G	G	G	G	G	C	A	A	-	-	-	-	-	T	C	T	T	T	A	A	A	T	C	T	A	G	C	T	A
N22	-	-	-	-	-	-	-	-	-	-	-	-	-	-	-	-	-	-	-	-	-	-	-	-	-	-	-	-	-	-	-	-
N23	-	-	-	-	-	-	-	-	-	-	-	-	-	-	-	-	-	-	-	-	-	-	-	-	-	-	-	-	-	-	-	-
N24	G	T	-	G	G	G	G	G	C	A	A	-	-	-	-	-	T	C	T	T	T	A	A	A	T	C	T	A	G	C	T	A
N25	G	T	-	G	G	G	G	G	C	A	A	-	-	-	-	-	T	C	T	T	T	A	A	A	T	C	T	A	G	C	T	A
N26	G	T	T	-	G	G	G	G	C	A	A	-	-	-	-	-	T	C	T	T	T	A	A	A	T	T	T	A	A	C	T	A
N27	G	T	T	-	G	G	G	G	C	A	A	-	-	-	-	-	T	C	T	T	T	A	A	A	T	T	T	A	A	C	T	A
N28	G	T	T	-	G	G	G	G	C	A	A	-	-	-	-	-	T	C	T	T	T	A	A	A	T	T	T	A	A	C	T	A
N29	-	-	-	-	-	-	-	-	-	-	-	-	-	-	-	-	-	-	-	-	-	-	-	-	-	-	-	-	-	-	-	-
N30	G	T	T	-	G	G	G	G	C	A	A	-	-	-	-	-	T	C	T	T	T	A	A	A	T	T	T	A	A	C	T	A
N31	G	T	-	G	G	G	G	G	C	A	A	T	A	T	A	A	T	C	T	T	T	A	A	A	T	C	T	A	G	C	T	A
N32	G	T	-	G	G	G	G	G	C	A	A	-	-	-	-	-	T	C	T	T	T	A	A	A	T	C	T	A	G	C	T	A
N33	G	T	T	-	G	G	G	G	C	A	A	-	-	-	-	-	T	C	T	T	T	A	A	A	T	T	T	A	A	C	T	A
N34	G	T	T	-	G	G	G	G	C	A	A	-	-	-	-	-	T	C	T	T	T	A	A	A	T	T	T	A	A	C	T	A
N35	G	T	T	-	G	G	G	G	C	A	A	-	-	-	-	-	T	C	T	T	T	A	A	A	T	T	T	A	A	C	T	A
N36	G	T	T	-	G	G	G	G	C	A	A	-	-	-	-	-	T	C	T	T	T	A	A	A	T	T	T	A	A	C	T	A
N37	G	T	-	G	G	G	G	G	C	A	A	-	-	-	-	-	T	C	T	T	T	A	A	A	T	C	T	A	G	C	T	A
N38	G	T	T	-	G	G	G	G	C	A	A	-	-	-	-	-	T	C	T	T	T	A	T	A	T	T	T	A	A	C	T	A
N39	G	T	T	-	G	G	G	G	C	A	A	-	-	-	-	-	T	C	T	T	T	A	A	A	T	T	T	A	A	C	T	A
N40	G	T	G	G	G	G	G	G	C	A	A	-	-	-	-	-	T	C	T	T	T	A	A	A	T	C	T	A	C	C	T	A

Table SA2. (continued).

Haplo-type	Variable nucleotide site in alignment																														
	210	211	212	213	214	215	216	217	218	219	220	221	222	223	224	225	226	227	228	229	230	231	232	233	234	235	236	237	238	239	240	241
H1	-	-	-	-	-	-	-	-	-	-	-	-	-	-	-	-	-	-	-	-	-	-	-	-	-	-	-	-	-	-	-	-
H2	-	-	-	-	-	-	-	-	-	-	-	-	-	-	-	-	-	-	-	-	-	-	-	-	-	-	-	-	-	-	-	-
H3	-	-	-	-	-	-	-	-	-	-	-	-	-	-	-	-	-	-	-	-	-	-	-	-	-	-	-	-	-	-	-	-
H4	G	C	T	A	G	C	A	A	A	A	A	G	A	A	C	A	T	T	C	A	T	T	T	G	C	T	T	T	-	-	-	A
H5	-	-	-	-	-	-	-	-	-	-	-	-	-	-	-	-	-	-	-	-	-	-	-	-	-	-	-	-	-	-	-	-
H6	-	-	-	-	-	-	-	-	-	-	-	-	-	-	-	-	-	-	-	-	-	-	-	-	-	-	-	-	-	-	-	-
H7	-	-	-	-	-	-	-	-	-	-	-	-	-	-	-	-	-	-	-	-	-	-	-	-	-	-	-	-	-	-	-	-
H8	-	-	-	-	-	-	-	-	-	-	-	-	-	-	-	-	-	-	-	-	-	-	-	-	-	-	-	-	-	-	-	-
H9	-	-	-	-	-	-	-	-	-	-	-	-	-	-	-	-	-	-	-	-	-	-	-	-	-	-	-	-	-	-	-	-
H10	-	-	-	-	-	-	-	-	-	-	-	-	-	-	-	-	-	-	-	-	-	-	-	-	-	-	-	-	-	-	-	-
H11	-	-	-	-	-	-	-	-	-	-	-	-	-	-	-	-	-	-	-	-	-	-	-	-	-	-	-	-	-	-	-	-
H12	-	-	-	-	-	-	-	-	-	-	-	-	-	-	-	-	-	-	-	-	-	-	-	-	-	-	-	-	-	-	-	-
H13	-	-	-	-	-	-	-	-	-	-	-	-	-	-	-	-	-	-	-	-	-	-	-	-	-	-	-	-	-	-	-	-
H14	-	-	-	-	-	-	-	-	-	-	-	-	-	-	-	-	-	-	-	-	-	-	-	-	-	-	-	-	-	-	-	-
H15	-	-	-	-	-	-	-	-	-	-	-	-	-	-	-	-	-	-	-	-	-	-	-	-	-	-	-	-	-	-	-	-
H16	-	-	-	-	-	-	-	-	-	-	-	-	-	-	-	-	-	-	-	-	-	-	-	-	-	-	-	-	-	-	-	-
H17	-	-	-	-	-	-	-	-	-	-	-	-	-	-	-	-	-	-	-	-	-	-	-	-	-	-	-	-	-	-	-	-
H18	-	-	-	-	-	-	-	-	-	-	-	-	-	-	-	-	-	-	-	-	-	-	-	-	-	-	-	-	-	-	-	-
H19	-	-	-	-	-	-	-	-	-	-	-	-	-	-	-	-	-	-	-	-	-	-	-	-	-	-	-	-	-	-	-	-
H20	G	C	T	A	G	C	A	A	A	A	A	G	A	A	C	A	T	T	C	A	T	T	T	G	C	T	T	T	A	-	T	A
H21	G	C	T	A	G	C	A	A	A	A	A	T	A	A	C	A	T	T	C	A	T	T	T	G	C	T	T	T	A	A	T	A
H22	-	-	-	-	-	-	-	-	-	-	-	-	-	-	-	-	-	-	-	-	-	-	-	-	-	-	-	-	-	-	-	-
H23	-	-	-	-	-	-	-	-	-	-	-	-	-	-	-	-	-	-	-	-	-	-	-	-	-	-	-	-	-	-	-	-
H24	G	C	T	A	G	C	A	A	A	A	A	G	A	A	C	A	T	T	C	A	T	T	T	G	C	T	T	T	A	A	T	A
H25	G	C	T	A	G	C	A	A	A	A	A	G	A	A	C	A	T	T	C	A	T	T	T	G	C	T	T	T	A	-	T	A
H26	G	C	-	-	-	-	A	A	A	A	A	G	A	A	C	A	T	T	C	A	T	T	T	G	C	T	T	T	-	-	T	A
H27	G	C	-	-	-	-	A	A	A	A	A	G	A	A	C	A	T	T	C	A	T	T	T	G	C	T	T	T	A	A	T	A
H28	G	C	-	-	-	-	A	A	A	A	A	G	A	A	C	A	T	T	C	A	T	T	T	G	C	T	T	T	A	A	T	A
H29	-	-	-	-	-	-	-	-	-	-	-	-	-	-	-	-	-	-	-	-	-	-	-	-	-	-	-	-	-	-	-	-
H30	G	C	-	-	-	-	A	A	A	A	A	G	A	A	C	A	T	T	C	A	T	T	T	G	C	T	T	T	A	A	T	A
H31	G	C	T	A	G	C	A	A	A	A	A	G	A	A	C	A	T	T	C	A	T	T	T	G	C	T	T	T	A	A	T	A
H32	G	C	T	A	G	C	A	A	A	A	A	G	A	A	C	A	T	T	C	A	T	T	T	G	C	T	T	T	A	-	T	A
H33	G	C	-	-	-	-	A	A	A	A	A	G	A	A	C	A	T	T	C	A	T	T	T	G	C	T	T	T	A	A	T	A
H34	G	C	-	-	-	-	A	A	A	A	A	G	A	A	C	A	T	T	C	A	T	T	T	G	C	T	T	T	A	A	T	A
H35	G	C	-	-	-	-	A	A	A	A	A	G	A	A	C	A	T	T	C	A	T	T	T	G	C	T	T	T	A	A	T	A
H36	G	C	-	-	-	-	A	A	A	A	A	G	A	A	C	A	T	T	C	A	T	T	T	G	C	T	T	T	A	A	A	A
H37	G	C	T	A	G	C	A	A	A	A	A	G	A	A	C	A	T	T	C	A	T	T	T	G	C	T	T	T	A	G	T	A
H38	G	C	-	-	-	-	A	A	A	A	A	G	A	G	C	A	T	T	C	A	T	T	T	G	C	T	T	T	T	A	T	A
H39	G	C	-	-	-	-	A	A	A	A	A	G	A	A	-	-	-	-	-	-	-	-	-	-	-	-	-	-	-	A	T	A
H40	G	C	T	A	G	C	A	A	A	A	A	G	A	A	C	A	T	T	C	A	T	T	T	G	C	T	T	T	A	A	T	A

Table SA2. (continued).

Haplo-type	Variable nucleotide site in alignment																														
	242	243	244	245	246	247	248	249	250	251	252	253	254	255	256	257	258	259	260	261	262	263	264	265	266	267	268	269	270	271	272	273
H1	-	-	-	-	-	-	-	-	-	-	-	-	-	-	-	-	-	-	-	-	-	-	-	-	-	-	-	-	-	-	-	-
H2	-	-	-	-	-	-	-	-	-	-	-	-	-	-	-	-	-	-	-	-	-	-	-	-	-	-	-	-	-	-	-	-
H3	-	-	-	-	-	-	-	-	-	-	-	-	-	-	-	-	-	-	-	-	-	-	-	-	-	-	-	-	-	-	-	-
H4	T	A	T	A	T	A	T	A	T	A	T	-	-	-	-	-	-	-	-	-	-	-	-	-	-	-	-	-	-	-	-	-
H5	-	-	-	-	-	-	-	-	-	-	-	-	-	-	-	-	-	-	-	-	-	-	-	-	-	-	-	-	-	-	-	-
H6	-	-	-	-	-	-	-	-	-	-	-	-	-	-	-	-	-	-	-	-	-	-	-	-	-	-	-	-	-	-	-	-
H7	-	-	-	-	-	-	-	-	-	-	-	-	-	-	-	-	-	-	-	-	-	-	-	-	-	-	-	-	-	-	-	-
H8	-	-	-	-	-	-	-	-	-	-	-	-	-	-	-	-	-	-	-	-	-	-	-	-	-	-	-	-	-	-	-	-
H9	-	-	-	-	-	-	-	-	-	-	-	-	-	-	-	-	-	-	-	-	-	-	-	-	-	-	-	-	-	-	-	-
H10	-	-	-	-	-	-	-	-	-	-	-	-	-	-	-	-	-	-	-	-	-	-	-	-	-	-	-	-	-	-	-	-
H11	-	-	-	-	-	-	-	-	-	-	-	-	-	-	-	-	-	-	-	-	-	-	-	-	-	-	-	-	-	-	-	-
H12	-	-	-	-	-	-	-	-	-	-	-	-	-	-	-	-	-	-	-	-	-	-	-	-	-	-	-	-	-	-	-	-
H13	-	-	-	-	-	-	-	-	-	-	-	-	-	-	-	-	-	-	-	-	-	-	-	-	-	-	-	-	-	-	-	-
H14	-	-	-	-	-	-	-	-	-	-	-	-	-	-	-	-	-	-	-	-	-	-	-	-	-	-	-	-	-	-	-	-
H15	-	-	-	-	-	-	-	-	-	-	-	-	-	-	-	-	-	-	-	-	-	-	-	-	-	-	-	-	-	-	-	-
H16	-	-	-	-	-	-	-	-	-	-	-	-	-	-	-	-	-	-	-	-	-	-	-	-	-	-	-	-	-	-	-	-
H17	-	-	-	-	-	-	-	-	-	-	-	-	-	-	-	-	-	-	-	-	-	-	-	-	-	-	-	-	-	-	-	-
H18	-	-	-	-	-	-	-	-	-	-	-	-	-	-	-	-	-	-	-	-	-	-	-	-	-	-	-	-	-	-	-	-
H19	-	-	-	-	-	-	-	-	-	-	-	-	-	-	-	-	-	-	-	-	-	-	-	-	-	-	-	-	-	-	-	-
H20	T	A	T	A	T	A	T	A	T	-	-	-	-	-	-	-	-	-	-	-	-	-	-	-	-	-	-	-	-	-	-	-
H21	T	A	T	A	T	A	T	-	-	-	-	-	-	-	-	-	-	-	-	C	C	C	T	C	T	C	T	A	T	A	T	G
H22	-	-	-	-	-	-	-	-	-	-	-	-	-	-	-	-	-	-	-	-	-	-	-	-	-	-	-	-	-	-	-	-
H23	-	-	-	-	-	-	-	-	-	-	-	-	-	-	-	-	-	-	-	-	-	-	-	-	-	-	-	-	-	-	-	-
H24	T	A	T	A	T	A	T	-	-	-	-	-	-	-	-	-	-	-	-	-	-	-	-	-	-	-	-	-	-	-	-	-
H25	T	A	T	A	T	A	T	A	T	-	-	-	-	-	-	-	-	-	-	-	-	-	-	-	-	-	-	-	-	-	-	-
H26	T	A	T	A	T	A	T	A	T	A	T	A	T	A	T	A	T	-	-	C	C	C	T	C	T	C	T	A	C	A	T	G
H27	T	A	T	A	T	A	T	A	T	A	T	A	T	A	T	A	T	-	-	C	C	C	T	C	T	C	T	A	T	A	T	G
H28	T	A	T	A	T	A	T	A	T	-	-	-	-	-	-	-	-	-	-	C	C	C	T	C	T	C	T	A	T	A	T	G
H29	-	-	-	-	-	-	-	-	-	-	-	-	-	-	-	-	-	-	-	-	-	-	-	-	-	-	-	-	-	-	-	-
H30	T	A	T	A	T	A	T	A	T	-	-	-	-	-	-	-	-	-	-	C	C	C	T	C	T	C	T	A	T	A	T	G
H31	T	A	T	A	T	A	T	A	-	A	T	A	T	-	-	-	-	-	-	C	C	C	T	C	T	C	T	A	C	A	T	G
H32	T	A	T	A	T	A	T	A	T	-	-	-	-	-	-	-	-	-	-	-	-	-	-	-	-	-	-	-	-	-	-	-
H33	T	A	T	A	T	-	-	-	-	-	-	-	-	-	-	-	-	-	-	C	C	C	T	C	T	C	T	A	C	A	T	G
H34	T	A	T	A	T	A	T	A	T	-	-	-	-	-	-	-	-	-	-	C	C	C	T	C	T	C	T	A	T	A	T	G
H35	T	A	T	A	T	A	T	A	T	-	-	-	-	-	-	-	-	-	-	C	C	C	T	C	T	C	T	A	T	A	T	G
H36	T	A	T	A	T	A	T	A	T	A	T	A	T	-	-	-	-	-	-	C	C	C	T	C	T	C	T	A	T	A	T	G
H37	T	A	T	A	T	A	T	A	T	A	T	A	T	A	T	A	T	-	-	C	C	C	T	C	T	C	T	A	C	A	T	G
H38	T	A	T	A	T	A	T	A	T	A	T	A	A	A	T	-	G	A	C	A	A	C	T	C	T	C	T	A	C	A	T	G
H39	T	A	T	A	T	A	T	A	T	A	T	A	T	-	-	-	-	-	-	-	-	-	-	-	-	-	-	-	-	A	T	G
H40	T	A	T	A	T	-	-	-	-	-	-	-	-	-	-	-	-	C	C	C	T	C	T	C	T	A	C	A	T	G	T	A

Table SA2. (continued).

	Variable nucleotide site in alignment																														
Haplo-type	274	275	276	277	278	279	280	281	282	283	284	285	286	287	288	289	290	291	292	293	300	301	302	303	304	305	306	307	308	309	310	311
N1	-	-	-	-	-	-	-	T	A	G	C	T	A	A	A	A	A	T	A	T	-	-	G	A	A	G	A	G	T	A	A	A
N2	-	-	-	-	-	-	-	.	.	.	.	.	.	.	.	.	.	.	.	.	-	-	.	.	.	.	.	.	.	.	.	.
N3	-	-	-	-	-	-	-	.	.	.	.	.	.	.	.	.	.	.	.	.	-	-	.	.	.	.	.	.	.	.	.	.
N4	-	-	-	-	-	-	-	-	-	-	-	-	-	-	-	-	-	-	-	-	-	-	.	.	.	.	.	.	.	.	.	.
N5	-	-	-	-	-	-	-	.	.	.	.	.	.	.	.	.	.	.	.	.	-	-	.	.	.	.	.	.	.	.	.	.
N6	-	-	-	-	-	-	-	.	.	.	.	.	.	.	.	.	.	.	.	.	-	-	.	.	.	.	.	.	.	.	.	.
N7	-	-	-	-	-	-	-	.	.	.	.	.	.	.	.	.	.	.	.	.	-	-	.	.	.	.	.	.	.	.	.	.
N8	-	-	-	-	-	-	-	.	.	.	.	.	.	.	.	.	.	.	.	.	-	-	.	.	.	.	.	.	.	.	.	.
N9	-	-	-	-	-	-	-	.	.	.	.	.	.	.	.	.	.	.	.	.	-	-	.	.	.	.	.	.	.	.	.	.
N10	-	-	-	-	-	-	-	.	.	.	.	.	.	.	.	.	.	.	.	.	-	-	.	.	.	.	.	.	.	.	.	.
N11	-	-	-	-	-	-	-	.	.	.	.	.	.	.	.	.	.	.	.	.	-	-	.	.	.	.	.	.	.	.	.	.
N12	-	-	-	-	-	-	-	.	.	.	.	.	.	.	.	.	.	.	.	.	-	-	.	.	.	.	.	.	.	.	.	.
N13	-	-	-	-	-	-	-	-	-	-	-	-	-	-	.	.	.	.	.	.	-	-	.	.	.	.	.	.	.	.	.	.
N14	-	-	-	-	-	-	-	.	.	.	.	.	.	.	.	.	.	.	.	.	-	-	.	.	.	.	.	.	.	.	.	.
N15	-	-	-	-	-	-	-	-	-	-	-	-	-	-	.	.	.	.	.	.	-	-	.	.	.	.	.	.	.	.	.	.
N16	-	-	-	-	-	-	-	.	.	.	.	.	.	.	.	.	.	.	.	.	-	-	.	.	.	.	.	.	.	.	.	.
N17	-	-	-	-	-	-	-	.	.	.	.	.	.	.	.	.	.	.	.	.	-	-	.	.	.	.	.	.	.	.	.	.
N18	-	-	-	-	-	-	-	.	.	.	.	.	.	.	.	.	.	.	.	.	-	-	.	.	.	.	.	.	.	.	.	.
N19	-	-	-	-	-	-	-	-	-	-	-	-	-	-	-	-	-	-	-	-	-	-	-	-	-	-	-	-	-	-	-	-
N20	-	-	-	-	-	-	-	-	-	-	-	-	-	-	-	-	-	-	-	-	-	-	.	.	.	.	.	.	.	.	.	.
N21	T	A	G	C	T	A	C	.	.	.	.	.	.	.	.	.	.	.	.	.	-	-	.	.	.	.	.	.	.	.	.	.
N22	-	-	-	-	-	-	-	.	.	.	.	.	.	.	.	.	.	.	.	.	-	-	.	.	.	.	.	.	.	.	.	.
N23	-	-	-	-	-	-	-	-	-	-	-	-	-	-	-	-	-	-	-	-	-	-	-	-	-	-	-	-	-	-	-	-
N24	-	A	G	C	T	A	C	.	.	.	.	.	.	.	.	.	.	.	.	.	-	-	.	.	.	.	.	.	.	.	.	.
N25	-	-	-	-	-	-	-	-	-	-	-	-	-	-	-	-	-	-	-	-	-	-	.	.	.	.	.	.	.	.	.	.
N26	T	A	G	C	T	A	C	.	.	.	.	.	.	.	.	.	.	.	.	.	-	-	.	.	.	.	.	.	.	.	.	.
N27	T	A	G	C	T	A	C	.	.	.	.	.	.	.	.	.	.	.	.	.	-	-	.	.	.	.	.	.	.	.	.	.
N28	T	A	G	C	T	A	C	.	.	.	.	.	.	.	.	.	.	.	.	.	-	-	.	.	.	.	.	.	.	.	.	.
N29	-	-	-	-	-	-	-	-	-	-	-	-	-	-	-	-	-	-	-	-	-	-	-	-	-	-	-	-	-	-	-	-
N30	T	A	G	C	T	A	C	.	.	.	.	.	.	.	.	.	.	.	.	.	-	-	.	.	.	.	.	.	.	.	.	.
N31	T	A	G	C	T	A	C	.	.	.	.	.	.	.	.	.	.	.	.	.	-	-	.	.	.	.	.	.	.	.	.	.
N32	-	-	-	-	-	-	-	-	-	-	-	-	-	-	-	-	-	-	-	-	G	T	.	.	.	.	.	.	.	.	.	.
N33	T	A	G	C	T	A	C	.	.	.	.	.	.	.	.	.	.	.	.	.	-	-	.	.	.	.	.	.	.	.	.	.
N34	T	A	G	C	T	A	C	.	.	.	.	.	.	.	.	.	.	.	.	.	-	-	.	.	.	.	.	.	.	.	.	.
N35	T	A	G	C	T	A	C	.	.	.	.	.	.	.	.	.	.	.	.	.	-	-	.	.	.	.	.	.	.	.	.	.
N36	T	A	G	C	T	A	C	.	.	.	.	.	.	.	.	.	.	.	.	.	-	-	.	.	.	.	.	.	.	.	.	.
N37	T	A	G	C	T	A	C	.	.	.	.	.	.	.	.	.	.	.	.	.	-	-	.	.	.	.	.	.	.	.	.	.
N38	T	A	G	C	T	A	C	.	.	.	.	.	.	.	.	.	.	.	.	.	-	-	.	.	.	.	.	.	.	.	.	.
N39	C	T	A	C	.	.	.	.	.	.	.	.	.	.	.	.	.	-	-	.	.	.	.	.	.	.	.	.	.	.	.	.
N40	C	T	A	C	.	.	.	.	.	.	.	.	.	.	.	.	.	-	-	.	.	.	.	.	.	.	.	.	.	.	.	.

Table SA2. (continued).

Haplo-type	Variable nucleotide site in alignment																														
	312	313	314	315	316	317	318	319	320	321	322	323	324	325	326	327	328	329	330	331	332	333	334	335	336	337	338	339	340	341	342	343
N1	G	A	C	A	A	A	T	A	-	C	A	G	T	G	G	G	-	-	-	-	-	A	A	A	A	T	G	A	G	G	T	A
N2	.	.	.	.	.	.	.	.	-	.	.	.	.	.	.	.	-	-	-	-	-	.	.	.	.	.	.	.	.	.	.	.
N3	.	.	.	.	.	.	.	.	-	.	.	.	.	.	.	.	-	-	-	-	-	.	.	.	.	.	.	.	.	.	.	.
N4	.	.	.	.	.	.	.	.	-	.	.	.	.	.	.	.	-	-	-	-	-	.	.	.	.	C	.	.	.	.	C	.
N5	.	.	.	.	.	.	.	.	-	.	.	.	.	.	.	.	-	-	-	-	-	.	.	.	.	.	.	.	.	.	.	.
N6	.	.	.	.	.	.	.	.	-	.	.	.	.	.	.	.	-	-	-	-	-	.	.	.	.	.	.	.	.	.	.	.
N7	.	.	.	.	.	.	.	.	-	.	.	.	.	.	.	.	-	-	-	-	-	.	.	.	.	.	.	.	.	.	.	.
N8	.	.	.	.	.	.	.	.	-	.	.	.	.	.	.	.	-	-	-	-	-	.	.	.	.	.	.	.	.	.	.	.
N9	.	.	.	.	.	.	.	.	-	.	.	.	.	.	.	.	-	-	-	-	-	.	.	.	.	.	.	.	.	.	.	.
N10	.	.	.	.	.	.	.	.	-	.	.	.	.	.	.	.	-	-	-	-	-	.	.	.	.	.	.	.	.	.	.	.
N11	.	.	.	.	.	.	.	.	-	.	.	.	.	.	.	.	-	-	-	-	-	.	.	.	.	.	.	.	.	.	.	.
N12	.	.	.	.	.	.	.	.	-	.	.	.	.	.	.	.	-	-	-	-	-	.	.	.	.	.	.	.	.	.	.	.
N13	.	.	.	.	.	.	.	.	-	.	.	.	.	.	.	.	-	-	-	-	-	.	.	.	.	.	.	.	.	.	.	.
N14	.	.	.	.	.	.	.	.	-	.	.	.	.	.	.	.	-	-	-	-	-	.	.	.	.	.	.	.	.	.	.	.
N15	.	.	.	.	.	.	.	.	-	.	.	.	.	.	.	.	-	-	-	-	-	.	.	.	.	.	.	.	.	.	.	.
N16	.	.	.	.	.	.	.	.	-	.	.	.	.	.	.	.	-	-	-	-	-	.	.	.	.	.	.	.	.	.	.	.
N17	.	.	.	.	.	.	.	.	-	.	.	.	.	.	.	-	-	-	-	-	-	.	.	.	.	.	.	.	.	.	.	.
N18	.	.	.	.	.	.	.	.	-	.	.	.	.	.	.	.	-	-	-	-	-	.	.	.	.	.	.	.	.	.	.	.
N19	-	-	-	-	-	-	-	-	-	-	-	-	-	-	-	-	-	-	-	-	-	-	-	-	-	-	-	-	-	-	-	-
N20	.	.	.	.	.	.	.	.	-	.	.	.	.	.	.	.	-	-	-	-	-	.	.	.	.	.	.	.	.	.	C	.
N21	.	.	.	.	.	.	.	.	-	.	.	.	.	.	.	.	-	-	-	-	-	.	.	.	.	.	.	.	.	.	.	.
N22	.	.	.	.	.	.	.	.	-	.	.	.	.	.	.	.	-	-	-	-	-	.	.	.	.	.	.	G	.	.	.	.
N23	-	-	-	-	-	-	-	-	-	-	-	-	-	-	-	-	-	-	-	-	-	-	-	-	-	-	-	-	-	-	-	-
N24	.	.	.	.	.	.	.	.	-	.	.	.	.	.	.	.	-	-	-	-	-	.	.	.	.	.	.	.	.	.	.	.
N25	.	.	.	.	.	.	.	.	-	.	.	.	.	.	.	.	-	-	-	-	-	.	.	.	.	.	.	.	.	.	C	.
N26	.	.	.	.	.	.	.	.	-	.	.	.	.	.	.	.	-	-	-	-	-	.	.	.	.	.	.	.	.	.	.	.
N27	.	.	.	.	.	.	.	.	-	.	.	.	.	.	.	.	-	-	-	-	-	.	.	.	.	.	.	.	.	.	.	.
N28	.	.	.	.	.	.	.	.	C	.	.	.	.	.	.	.	-	-	-	-	-	.	.	.	.	.	.	.	.	.	.	.
N29	-	-	-	-	-	-	-	-	-	-	-	-	-	-	-	-	-	-	-	-	-	-	-	-	-	-	-	-	-	-	-	-
N30	.	.	.	.	.	.	.	.	C	.	.	.	.	.	.	.	-	-	-	-	-	.	.	.	.	.	.	.	.	.	.	.
N31	.	.	.	.	.	.	.	.	-	.	.	.	.	.	.	.	-	-	-	-	-	.	.	.	.	.	.	.	.	.	.	.
N32	.	.	.	.	.	.	.	.	-	.	.	.	.	.	.	.	A	A	A	T	T	.	.	.	.	.	.	.	.	.	.	.
N33	.	.	.	.	.	.	.	.	-	.	.	.	.	.	.	.	-	-	-	-	-	.	.	.	.	.	.	.	.	.	.	.
N34	.	.	.	.	.	.	.	.	-	.	.	.	.	.	.	.	-	-	-	-	-	.	.	.	.	.	.	.	.	.	.	.
N35	.	.	.	.	.	.	.	.	-	.	.	.	.	.	.	.	-	-	-	-	-	.	.	.	.	.	.	.	.	.	.	.
N36	.	.	.	.	.	.	.	.	-	.	.	.	.	.	.	.	-	-	-	-	-	.	.	.	.	.	.	.	.	.	.	.
N37	.	.	.	.	.	.	.	.	-	.	.	.	.	.	.	.	-	-	-	-	-	.	.	.	.	.	A	.	.	.	.	.
N38	.	.	.	.	.	.	.	.	-	.	.	.	.	.	.	.	-	-	-	-	-	.	.	.	.	.	.	.	.	.	.	.
N39	.	.	.	.	.	.	.	.	-	.	.	.	.	.	.	.	-	-	-	-	-	.	.	.	.	.	.	.	.	.	.	.
N40	.	.	.	.	.	.	.	.	-	.	.	.	.	.	.	.	-	-	-	-	-	.	.	.	.	.	.	.	.	.	.	.

Table SA2. (continued).

Haplo-type	Variable nucleotide site in alignment																														
	344	345	346	347	348	349	350	351	352	353	354	355	356	357	358	359	361	362	363	364	365	366	367	368	369	370	371	372	373	374	375	376
N1	A	G	A	C	A	T	T	G	A	C	G	A	G	T	A	T	A	C	A	T	A	T	A	A	T	G	A	T	G	G	A	C
N2	.	.	.	.	.	.	.	.	.	.	.	.	A	.	.	.	.	.	.	.	.	.	.	.	.	.	.	.	.	.	.	.
N3	.	.	.	.	.	.	.	.	.	.	.	.	.	.	.	.	.	.	.	.	.	.	.	.	.	.	.	.	.	.	.	.
N4	.	.	.	G	.	.	.	.	.	.	T	.	.	.	.	.	.	.	.	.	.	.	.	.	.	.	.	.	.	.	.	.
N5	.	.	.	.	.	.	.	.	.	.	.	.	A	.	.	.	.	.	.	.	.	.	.	.	.	.	.	.	.	.	.	.
N6	.	.	.	.	.	.	.	.	.	.	.	.	.	.	.	.	.	.	.	.	.	.	.	.	.	.	.	.	.	.	.	.
N7	.	.	.	.	.	.	.	.	.	.	.	.	.	.	.	.	.	.	.	.	.	.	.	.	.	.	.	.	.	.	.	.
N8	.	.	.	.	.	.	.	.	.	.	.	.	.	.	.	.	.	.	.	.	.	.	.	.	.	.	.	.	.	.	.	.
N9	.	.	.	.	.	.	.	.	.	.	.	.	.	.	.	.	.	.	.	.	.	.	.	.	.	.	.	.	.	.	.	.
N10	.	.	.	.	.	.	.	.	.	.	.	.	.	.	.	.	.	.	.	.	.	.	.	.	.	.	.	.	.	.	.	.
N11	.	.	.	.	.	.	.	.	.	.	.	.	.	.	.	.	.	.	.	.	.	.	.	.	.	.	.	.	.	.	.	.
N12	.	.	.	.	.	.	.	.	.	.	.	.	.	.	.	.	.	.	.	.	.	.	.	.	.	.	.	.	.	.	.	.
N13	.	.	.	.	.	.	.	.	.	.	.	.	.	.	.	.	.	.	.	.	.	.	.	.	.	.	.	.	.	.	.	.
N14	.	.	.	.	.	.	.	.	.	.	.	.	.	.	.	.	.	.	.	.	.	.	.	.	.	.	.	.	.	.	.	.
N15	.	.	.	.	.	.	.	.	.	.	.	.	.	.	.	.	.	.	.	.	.	.	.	.	.	.	.	.	.	.	.	.
N16	.	.	.	.	.	.	.	.	.	.	.	.	.	.	.	.	.	.	.	.	.	.	.	.	.	.	.	.	.	.	.	.
N17	.	.	.	.	.	.	.	.	.	.	.	.	.	.	.	.	.	.	.	.	.	.	.	.	.	.	.	.	.	.	.	.
N18	.	.	.	.	.	.	.	.	.	.	.	.	.	.	.	.	.	.	.	.	.	.	.	.	.	.	.	.	.	.	.	.
N19	-	-	-	-	-	-	-	-	-	-	-	-	-	-	-	-	-	-	-	-	-	-	-	-	-	.	.	.	.	.	.	.
N20	.	.	.	G	.	.	.	.	.	.	T	.	.	.	.	.	.	.	.	.	.	.	.	.	.	.	.	.	.	.	.	.
N21	.	.	.	G	.	.	.	.	.	A	.	T	.	.	.	.	.	.	.	.	.	.	.	.	.	.	.	.	.	.	.	.
N22	.	.	.	.	.	.	.	.	.	.	.	.	.	.	.	.	.	.	.	.	.	.	.	.	.	.	.	.	.	.	.	.
N23	-	-	-	-	-	-	-	-	-	-	-	-	-	-	-	-	-	-	-	-	-	-	-	-	-	.	.	.	.	.	.	.
N24	.	.	.	G	.	.	.	.	.	A	.	T	.	.	.	.	.	.	.	.	.	.	.	.	.	.	.	.	.	.	.	.
N25	.	.	.	G	.	.	.	.	.	.	T	.	.	.	.	.	.	.	.	.	.	.	.	.	.	.	.	.	.	.	.	.
N26	.	.	.	G	.	.	.	.	.	.	.	.	.	.	.	.	.	.	.	.	.	.	.	.	.	.	.	.	.	.	.	.
N27	.	.	.	G	.	.	.	.	.	A	.	T	.	.	.	.	.	.	.	.	.	.	.	.	.	.	.	.	.	.	.	.
N28	.	.	.	G	.	.	.	.	.	A	.	T	.	.	.	.	.	.	.	.	.	.	.	.	.	.	.	.	.	.	.	.
N29	-	-	-	-	-	-	-	-	-	-	-	-	-	-	-	-	-	-	-	-	-	-	-	-	-	-	-	-	-	-	-	-
N30	.	.	.	G	.	.	.	.	.	A	.	T	.	.	.	.	.	.	.	.	.	.	.	.	.	.	.	.	.	.	.	.
N31	.	.	.	G	.	.	.	.	.	A	.	T	.	.	.	.	.	.	.	.	-	-	.	.	.	.	.	.	.	.	.	.
N32	.	.	.	G	.	.	.	.	.	.	.	.	.	.	.	.	.	.	.	.	.	.	.	.	.	.	.	.	.	.	.	.
N33	.	.	.	G	.	.	.	.	.	.	.	.	.	.	.	.	.	.	.	.	.	.	.	.	.	.	.	.	.	.	.	.
N34	.	.	.	G	.	.	.	.	.	A	.	T	.	.	.	.	.	.	.	.	.	.	.	.	.	.	.	.	.	.	.	.
N35	.	.	.	G	.	.	.	.	.	A	.	T	.	.	.	.	.	.	.	.	.	.	.	.	.	.	.	.	.	.	.	.
N36	.	.	.	G	.	.	.	.	.	A	.	T	.	.	.	.	.	.	.	.	.	.	.	.	.	.	.	.	.	.	.	.
N37	.	.	.	G	.	.	.	.	.	A	.	T	.	.	.	.	.	.	.	.	.	.	.	.	.	.	.	.	.	.	.	.
N38	.	.	.	G	.	.	.	.	.	.	.	.	.	.	.	.	.	.	.	.	.	.	.	.	.	.	.	.	.	.	.	.
N39	.	.	.	G	.	.	.	.	.	A	.	T	.	.	.	.	.	.	.	.	.	.	.	.	.	.	.	.	.	.	.	.
N40	.	.	.	G	.	.	.	.	.	.	.	.	.	.	.	.	.	.	.	.	.	.	.	.	.	.	.	.	.	.	.	.

Table SA2. (continued).

Haplo-type	377	378	379	380	381	382	383	384	385	386	387	388	389	390	391	392	393	394	395	396	397	398	399	400	401	402	403	404	405	406	407	408
	Variable nucleotide site in alignment																															
N1	T	A	A	T	G	T	T	T	G	C	C	G	A	A	T	A	A	C	A	A	T	T	C	A	A	A	A	C	T	T	A	T
N2	.	.	.	.	.	.	.	.	.	.	.	.	.	.	.	.	.	.	.	.	.	.	.	.	.	.	.	.	.	.	.	.
N3	.	.	.	.	.	.	.	.	.	.	.	.	.	.	.	.	.	.	.	.	.	.	.	.	.	.	.	.	.	.	.	.
N4	.	.	.	.	.	.	.	.	.	.	A	.	.	.	.	.	.	.	.	.	.	.	.	.	.	.	.	.	.	.	.	.
N5	.	.	.	.	.	.	.	.	.	.	.	.	.	.	.	.	.	.	.	.	.	.	.	.	.	.	.	.	.	.	.	.
N6	.	.	.	.	.	.	.	.	.	.	.	.	.	.	.	.	.	.	.	.	.	.	.	.	.	.	.	.	.	.	.	.
N7	.	.	.	.	.	.	.	.	.	.	.	.	.	.	.	.	.	.	.	.	.	.	.	.	.	.	.	.	.	.	.	.
N8	.	.	.	.	.	.	.	.	.	.	A	.	.	.	.	.	.	.	.	.	.	.	.	.	.	.	.	.	.	.	.	.
N9	.	.	.	.	.	.	.	.	.	.	A	.	.	.	.	.	.	.	.	.	.	.	.	.	.	.	.	.	.	.	.	.
N10	.	.	.	.	.	.	.	.	.	.	.	.	.	.	.	.	.	.	.	.	.	.	.	.	.	.	.	.	.	.	.	.
N11	.	.	.	.	.	.	.	.	.	.	A	.	.	.	.	.	.	.	.	.	.	.	.	.	.	.	.	.	.	.	.	.
N12	.	.	.	.	.	.	.	.	.	.	.	.	.	.	.	.	.	.	.	.	.	.	.	.	.	.	.	.	.	.	.	.
N13	.	.	.	.	.	.	.	.	.	.	.	.	.	.	.	.	.	.	.	.	.	.	.	.	.	.	.	.	.	.	.	.
N14	.	.	.	.	.	.	.	.	.	.	.	.	.	.	.	.	.	.	.	.	.	.	.	.	.	.	.	.	.	.	.	.
N15	.	.	.	.	.	.	.	.	.	.	.	.	.	.	.	.	.	.	.	.	.	.	.	.	.	.	.	.	.	.	.	.
N16	.	.	.	.	.	.	.	.	.	.	.	.	.	.	.	.	.	.	.	.	.	.	.	.	.	.	.	.	.	.	.	.
N17	.	.	.	.	.	.	.	.	.	.	.	.	.	.	.	.	.	.	.	.	.	.	.	.	.	.	.	.	.	.	.	.
N18	.	.	.	.	.	.	.	.	.	.	.	.	.	.	.	.	.	.	.	.	.	.	.	.	.	.	.	.	.	.	.	.
N19	.	.	.	.	.	.	.	.	.	.	A	.	.	.	.	.	.	.	.	.	.	.	.	.	.	.	.	.	.	.	.	.
N20	.	.	.	.	.	.	.	.	.	.	A	.	.	.	.	.	.	.	.	.	.	.	.	.	.	.	.	.	.	.	.	.
N21	.	.	.	.	.	.	.	.	.	.	A	.	.	.	.	.	.	.	.	.	.	.	.	.	.	.	.	.	.	.	.	.
N22	.	.	.	.	.	.	.	.	.	.	.	.	.	.	.	.	.	.	.	.	.	.	.	.	.	.	.	.	.	.	.	.
N23	.	.	.	.	.	.	.	.	.	.	A	.	.	.	.	.	.	.	.	.	.	.	.	.	.	.	.	.	.	.	.	.
N24	.	.	.	.	.	.	.	.	.	.	A	.	.	.	.	.	.	.	.	.	.	.	.	.	.	.	.	.	.	.	.	.
N25	.	.	.	.	.	.	.	.	.	.	A	.	.	.	.	.	.	.	.	.	.	.	.	.	.	.	.	.	.	.	.	.
N26	.	.	.	.	.	.	A	.	.	.	A	.	.	.	.	.	.	.	.	.	.	.	.	.	.	.	.	.	.	.	.	.
N27	.	.	.	.	.	.	.	.	.	.	A	.	.	.	.	.	.	.	.	.	.	.	.	.	.	.	.	.	.	.	.	.
N28	.	.	.	.	.	.	.	.	.	.	A	.	.	.	.	.	.	.	.	.	.	.	.	.	.	.	.	.	.	.	.	.
N29	-	-	-	-	-	-	-	-	-	-	-	-	-	-	-	-	-	-	-	-	-	-	-	-	-	-	-	-	-	-	-	-
N30	.	.	.	.	.	.	.	.	.	.	A	.	.	.	.	.	.	.	.	.	.	.	.	.	.	.	.	.	.	.	.	.
N31	.	.	.	.	.	.	.	.	.	.	A	.	.	.	.	.	.	.	.	.	.	.	.	.	.	.	.	.	.	.	.	.
N32	.	.	.	.	.	.	.	.	.	.	A	.	.	.	.	.	.	.	.	.	.	.	.	.	.	.	.	.	.	.	.	.
N33	.	.	.	.	.	.	.	.	.	.	A	.	.	.	.	.	.	.	.	.	.	.	.	.	.	.	.	.	.	.	.	.
N34	.	.	.	.	.	.	.	.	.	.	A	.	.	.	.	.	.	.	.	.	.	.	.	.	.	.	.	.	.	.	.	.
N35	.	.	.	.	.	.	.	.	.	.	A	.	.	.	.	.	.	.	.	.	.	.	.	.	.	.	.	.	.	.	.	.
N36	.	.	.	.	.	.	.	.	.	.	A	.	.	.	.	.	.	.	.	.	.	.	.	.	.	.	.	.	.	.	.	.
N37	.	.	.	.	.	.	.	.	.	.	A	.	.	.	.	.	.	.	.	.	.	.	.	.	.	.	.	.	.	.	.	.
N38	.	.	.	.	.	.	A	.	.	.	A	.	.	.	.	.	.	.	.	.	.	.	.	.	.	.	.	.	.	.	.	.
N39	.	.	.	.	.	.	.	.	.	.	A	.	.	.	.	.	.	.	.	.	.	.	.	.	.	.	G	.	.	.	.	.
N40	.	.	.	.	.	.	.	.	.	.	A	.	.	.	.	.	.	.	.	.	.	.	.	.	.	.	.	.	.	.	.	.

Table SA2. (continued).

	Variable nucleotide site in alignment																														
	4	4	4	4	4	4	4	4	4	4	4	4	4	4	4	4	4	4	4	4	4	4	4	4	4	4	4	4	4	4	4	4
Haplo	0	1	1	1	1	1	1	1	1	1	1	2	2	2	2	2	2	3	3	3	3	3	3	3	3	3	3	4	4	4	4	4
-type	9	0	1	2	3	4	5	6	7	8	9	0	1	6	7	8	9	0	1	2	3	4	5	6	7	8	9	0	1	2	3	4
N1	T	T	T	T	T	A	G	G	A	A	T	T	G	T	T	A	A	T	T	A	G	T	T	T	G	T	T	G	G	A	A	T
N2	.	.	.	.	.	.	.	.	.	.	.	.	.	.	.	.	.	.	.	.	.	.	.	.	.	.	.	.	.	.	.	.
N3	.	.	.	.	.	.	.	.	.	.	.	.	.	.	.	.	.	.	.	.	.	.	.	.	.	.	.	.	.	.	.	.
N4	.	.	.	.	.	.	.	.	.	.	.	.	.	.	.	.	.	.	.	.	.	.	.	.	.	.	.	.	.	.	.	.
N5	.	.	.	.	.	.	.	.	.	.	.	.	.	.	.	.	.	.	.	.	.	.	.	.	.	.	.	.	.	.	.	.
N6	.	.	.	.	.	.	.	.	.	.	.	.	.	.	.	.	.	.	.	.	.	.	.	.	.	.	.	.	.	.	.	.
N7	.	.	.	.	.	.	.	.	.	.	.	.	.	.	.	.	.	.	.	.	.	.	.	.	.	.	.	.	.	.	.	.
N8	.	.	.	.	.	.	.	.	.	.	.	.	.	.	.	.	.	.	.	.	.	.	.	.	.	.	.	.	.	.	.	.
N9	.	.	.	.	.	.	.	.	.	.	.	.	.	.	.	.	.	.	.	.	.	.	.	.	.	.	.	.	.	.	.	.
N10	.	.	.	.	.	.	.	.	.	.	.	.	.	.	.	.	.	.	.	.	.	.	.	.	.	.	.	.	.	.	.	.
N11	.	.	.	.	.	.	.	.	.	.	.	.	.	.	.	.	.	.	.	.	.	.	.	.	.	.	.	.	.	.	.	.
N12	.	.	.	.	.	.	.	.	.	.	.	.	.	.	.	.	.	.	.	.	.	.	.	.	.	.	.	.	.	.	.	.
N13	.	.	.	.	.	.	.	.	.	.	.	.	.	.	.	.	.	.	.	.	.	.	.	.	.	.	.	.	.	.	.	.
N14	.	.	.	.	.	.	.	.	.	.	.	.	.	.	.	.	.	.	.	.	.	.	.	.	.	.	.	.	.	.	.	.
N15	.	.	.	.	.	.	.	.	.	.	.	.	.	.	.	.	.	.	.	.	.	.	.	.	.	.	.	.	.	.	.	.
N16	.	.	.	.	.	.	.	.	.	.	.	.	.	.	.	.	.	.	.	.	.	.	.	.	.	.	.	.	.	.	.	.
N17	.	.	.	.	.	.	.	.	.	.	.	.	.	.	.	.	.	.	.	.	.	.	.	.	.	.	.	.	.	.	.	.
N18	.	.	.	.	.	.	.	.	.	.	.	.	.	.	.	.	.	.	.	.	.	.	.	.	.	.	.	.	.	.	.	.
N19	.	.	.	.	.	.	.	.	.	.	.	.	.	.	.	.	.	.	.	.	.	.	.	.	.	.	.	.	.	.	.	.
N20	.	.	.	.	.	.	.	.	.	.	.	.	.	.	.	.	.	.	.	.	.	.	.	.	.	.	.	.	.	.	.	.
N21	.	.	.	.	.	.	.	.	.	.	.	.	.	.	.	.	.	.	.	.	.	.	.	.	.	.	.	.	.	.	.	.
N22	.	.	.	.	.	.	.	.	.	.	.	.	.	.	.	.	.	.	.	.	.	.	.	.	.	.	.	.	.	.	.	.
N23	.	.	.	.	.	.	.	.	.	.	.	.	.	.	.	.	.	.	.	.	.	.	.	.	.	.	.	.	.	.	.	.
N24	.	.	.	.	.	.	.	.	.	.	.	.	.	.	.	.	.	.	.	.	.	.	.	.	.	.	.	.	.	.	.	.
N25	.	.	.	.	.	.	.	.	.	.	.	.	.	.	.	.	.	.	.	.	.	.	.	.	.	.	.	.	.	.	.	.
N26	.	.	.	.	.	.	.	.	.	.	.	.	A	.	.	.	.	.	.	.	.	.	.	.	.	.	.	.	.	.	.	.
N27	.	.	.	.	.	.	.	.	.	.	.	.	.	.	.	.	.	.	.	.	.	.	.	.	.	.	.	.	.	.	.	.
N28	.	.	.	.	.	.	.	.	.	.	.	.	.	.	.	.	.	.	.	.	.	.	.	.	.	.	.	.	.	.	.	.
N29	-	-	-	-	-	-	-	-	-	-	-	-	-	-	-	-	-	-	-	-	-	-	-	-	-	-	-	-	-	-	-	-
N30	.	.	.	.	.	.	.	.	.	.	.	.	.	.	.	.	.	.	.	.	.	.	.	.	.	.	.	.	.	.	.	.
N31	.	.	.	.	.	.	.	.	.	.	.	.	.	.	.	.	.	.	.	.	.	.	.	.	.	.	.	.	.	.	.	.
N32	.	.	.	.	.	.	.	.	.	.	.	.	.	.	.	.	.	.	.	.	.	.	.	.	.	.	.	.	.	.	.	.
N33	.	.	.	.	.	.	.	.	.	.	.	.	.	.	.	.	.	.	.	.	.	.	.	.	.	.	.	.	.	.	.	.
N34	.	.	.	.	.	.	.	.	.	.	.	.	.	.	.	.	.	.	.	.	.	.	.	.	.	.	.	.	.	.	.	.
N35	.	.	.	.	.	.	.	.	.	.	.	.	.	.	.	.	.	.	.	.	.	.	.	.	.	.	.	.	.	.	.	.
N36	.	.	.	.	.	.	.	.	.	.	.	.	.	.	.	.	.	.	.	.	.	.	.	.	.	.	.	.	.	.	.	.
N37	.	.	.	.	.	.	.	.	.	.	.	.	.	.	.	.	.	.	.	.	.	.	.	.	.	.	.	.	.	.	.	.
N38	.	.	.	.	.	.	.	.	.	.	.	.	.	.	.	.	.	.	.	.	.	.	.	.	.	.	.	.	.	.	.	.
N39	.	.	.	.	.	.	.	.	.	.	.	.	.	.	.	.	.	.	.	.	.	.	.	.	.	.	.	.	.	.	.	.
N40	.	.	.	.	.	.	.	.	.	.	.	.	.	.	.	.	.	.	.	.	.	.	.	.	.	.	.	.	.	.	.	.

Table SA2. (continued).

	Variable nucleotide site in alignment																														
	4	4	4	4	4	4	4	4	4	4	4	4	4	4	4	4	4	4	4	4	4	4	4	4	4	4	4	4	4	4	4	4
Haplo	4	4	4	4	4	5	5	5	5	5	5	6	6	6	6	6	6	6	6	6	6	7	7	7	7	7	7	7	7	7	7	8
-type	5	6	7	8	9	0	1	2	3	4	9	0	1	2	3	4	5	6	7	8	9	0	1	2	3	4	5	6	7	8	9	0
N1	T	G	A	G	C	A	A	T	A	T	G	G	T	T	G	T	T	A	G	T	A	T	A	T	C	A	G	T	G	G	G	T
N2	.	.	.	.	.	.	.	.	.	.	.	.	.	.	.	A	.	.	.	.	.	.	.	.	.	.	.	.	.	.	.	.
N3	.	.	.	.	.	.	.	.	.	.	.	.	.	.	.	.	.	.	.	.	.	.	.	.	.	.	.	.	.	.	.	.
N4	.	.	.	A	.	.	.	.	.	.	.	.	.	.	.	.	.	.	.	.	.	.	.	.	.	.	.	.	.	.	.	.
N5	.	.	.	.	.	.	.	.	.	.	.	.	.	.	.	.	.	.	.	.	.	.	.	.	.	.	.	.	.	.	.	.
N6	.	.	.	.	.	.	.	.	.	.	.	.	.	.	.	.	.	.	.	.	.	.	.	.	.	.	.	.	.	.	.	.
N7	.	.	.	.	.	.	.	.	.	.	.	.	.	.	.	.	.	.	.	.	.	.	.	.	.	.	.	.	.	.	.	.
N8	.	.	.	.	.	.	.	.	.	.	.	.	.	.	.	.	.	.	.	.	.	.	.	.	.	.	.	.	.	.	.	.
N9	.	.	.	.	.	.	.	.	.	.	.	.	.	.	.	.	.	.	.	.	.	.	.	.	.	.	.	.	.	.	.	.
N10	.	.	.	.	.	.	.	.	.	.	.	.	.	.	.	.	.	.	.	.	.	.	.	.	.	.	.	.	.	.	.	.
N11	.	.	.	.	.	.	.	.	.	.	.	.	.	.	.	.	.	.	.	.	.	.	.	.	.	.	.	.	.	.	.	.
N12	.	.	.	.	.	.	.	.	.	.	.	.	.	.	.	.	.	.	.	.	.	.	.	.	.	.	.	.	.	.	.	.
N13	.	.	.	.	.	.	.	.	.	.	.	.	.	.	.	.	.	.	.	.	.	.	.	.	.	.	.	.	.	.	.	.
N14	.	.	.	.	.	.	.	.	.	.	.	.	.	.	.	.	.	.	.	.	T	.	.	.	.	.	.	.	.	.	.	.
N15	.	.	.	.	.	.	.	.	.	.	.	.	.	.	.	.	.	.	.	.	.	.	.	.	.	.	.	.	.	.	.	.
N16	.	.	.	.	.	.	.	.	.	.	.	.	.	.	.	.	.	.	.	.	.	.	.	.	.	.	.	.	.	.	.	.
N17	.	.	.	.	.	.	.	.	.	.	.	.	.	.	.	.	.	.	.	.	.	.	.	.	.	.	.	.	.	.	.	.
N18	.	.	.	.	.	.	.	.	.	.	.	.	.	.	.	.	.	.	.	.	.	.	.	.	.	.	.	.	.	.	.	.
N19	.	.	.	A	.	.	.	.	.	.	.	.	.	.	.	.	.	.	.	.	.	.	.	.	.	.	.	.	.	.	.	.
N20	.	.	.	A	.	.	.	.	.	.	.	.	.	.	.	.	.	.	.	.	.	.	.	.	.	.	.	.	.	.	.	.
N21	.	.	.	A	.	.	.	.	.	.	.	.	.	.	.	.	.	.	.	.	.	.	.	.	.	.	.	.	.	.	.	.
N22	.	.	.	.	.	.	.	.	.	.	.	.	.	.	.	.	.	.	.	.	.	.	.	.	.	.	.	.	.	.	.	.
N23	.	.	.	A	.	.	.	.	.	.	.	.	.	.	.	.	.	.	.	.	.	.	.	.	.	.	.	.	.	.	.	.
N24	.	.	.	A	.	.	.	.	.	.	.	.	.	.	.	.	.	.	.	.	.	.	.	.	.	.	.	.	.	.	.	.
N25	.	.	.	A	.	.	.	.	.	.	.	.	.	.	.	.	.	.	.	.	.	.	.	.	.	.	.	.	.	.	.	.
N26	.	.	.	A	.	.	.	.	.	.	.	.	.	.	.	.	.	.	.	.	.	.	.	.	.	.	.	.	.	.	.	.
N27	.	C	.	A	.	.	.	.	.	.	.	.	.	.	.	.	.	.	.	.	.	.	.	.	.	.	.	.	.	.	.	.
N28	.	.	.	A	.	.	.	.	.	.	.	.	.	.	.	.	.	.	.	.	.	.	.	.	.	.	.	.	.	.	.	.
N29	-	-	-	-	-	-	-	-	-	-	-	-	-	-	-	-	-	-	-	-	-	-	-	-	-	-	-	-	-	-	-	-
N30	.	.	.	A	.	.	.	.	.	.	.	.	.	.	.	.	.	.	.	.	.	.	.	.	.	.	.	.	.	.	.	.
N31	.	.	.	A	.	.	.	.	.	.	.	.	.	.	.	.	.	.	.	.	.	.	.	.	.	.	.	.	.	.	.	.
N32	.	.	.	A	.	.	.	.	.	.	.	.	.	.	.	.	.	.	.	.	.	.	.	.	.	.	.	.	.	.	.	.
N33	.	.	.	A	.	.	.	.	.	.	.	.	.	.	.	.	.	.	.	.	.	.	.	.	.	.	.	.	.	.	.	.
N34	.	C	.	A	.	.	.	.	.	.	.	.	.	.	.	.	.	.	.	.	.	.	.	.	.	.	.	.	.	.	.	.
N35	.	C	.	A	.	.	.	.	.	.	.	.	.	.	.	.	.	.	.	.	.	.	.	.	.	.	.	.	.	.	.	.
N36	.	C	.	A	.	.	.	.	.	.	.	.	.	.	.	.	.	.	.	.	.	.	.	.	.	.	.	.	.	.	.	.
N37	.	.	.	A	.	.	.	.	.	.	.	.	.	.	.	.	.	.	.	.	.	.	.	.	.	.	.	.	.	.	.	.
N38	.	.	.	A	.	.	.	.	.	.	.	.	.	.	.	.	.	.	.	.	.	.	.	.	.	.	.	.	.	.	.	.
N39	.	.	.	A	.	.	.	.	.	.	.	.	.	.	.	.	.	.	.	.	.	.	.	.	.	.	.	.	.	.	.	.
N40	.	.	.	A	.	.	.	.	.	.	-	-	-	-	-	-	-	-	-	-	-	-	-	-	-	-	-	-	-	-	-	-

Table SA2. (continued).

Haplo-type	Variable nucleotide site in alignment																														
	488	489	495	496	497	498	499	500	501	502	503	504	505	506	507	508	509	510	511	512	513	514	515	516	517	518	519	520	521	526	527	528
N1	C	A	T	A	T	A	T	A	C	T	T	G	A	G	G	A	T	T	A	T	A	A	A	T	T	A	T	C	C	T	A	T
N2	.	.	.	.	.	.	.	.	.	.	.	.	.	.	.	.	.	.	.	.	.	.	.	.	.	.	.	.	.	.	.	.
N3	.	.	.	.	.	.	.	.	.	.	.	.	.	.	.	.	.	.	.	.	.	.	.	.	.	.	.	.	.	.	.	.
N4	.	.	.	.	.	.	.	.	.	.	.	A	.	.	.	.	.	.	.	.	.	.	.	C	.	.	.	.	.	.	.	.
N5	.	.	.	.	.	.	.	.	.	.	.	.	.	.	.	.	.	.	.	.	.	.	.	.	.	.	.	.	.	.	.	.
N6	.	.	.	.	.	.	.	.	.	.	.	.	.	.	.	.	.	.	.	.	.	.	.	.	.	.	.	.	.	.	.	.
N7	.	.	.	.	.	.	.	.	.	.	.	.	.	.	.	.	.	.	.	.	.	.	.	.	.	.	.	.	.	.	.	.
N8	.	.	.	.	.	.	.	.	.	.	.	.	.	.	.	.	.	.	.	.	.	.	.	.	.	.	.	.	.	.	.	.
N9	.	.	.	.	.	.	.	.	.	.	.	.	.	.	.	.	.	.	.	.	.	.	.	.	.	.	.	.	.	.	.	.
N10	.	.	.	.	.	.	.	.	.	.	.	.	.	.	.	.	.	.	.	.	.	.	.	.	.	.	.	.	.	.	.	.
N11	.	.	.	.	.	.	.	.	.	.	.	.	.	.	.	.	.	.	.	.	.	.	.	.	.	.	.	.	.	.	.	.
N12	.	.	.	.	.	.	.	.	.	.	.	.	.	.	.	.	.	.	.	.	.	.	.	.	.	.	.	.	.	.	.	.
N13	.	.	.	.	.	.	.	.	.	.	.	.	.	.	.	.	.	.	.	.	.	.	.	.	.	.	.	.	.	.	.	.
N14	.	.	.	.	.	.	.	.	.	.	.	.	.	.	.	.	.	.	.	.	.	.	.	.	.	.	.	.	.	.	.	.
N15	.	.	.	.	.	.	.	.	.	.	.	.	.	.	.	.	.	.	.	.	.	.	.	.	.	.	.	.	.	.	.	.
N16	.	.	.	.	.	.	.	.	.	.	.	.	.	.	.	.	.	.	.	.	.	.	.	.	.	.	.	.	.	.	.	.
N17	.	.	.	.	.	.	.	.	.	.	.	.	.	.	.	.	.	.	.	.	.	.	.	.	.	.	.	.	.	.	.	.
N18	.	.	.	.	.	.	.	.	.	.	.	.	.	.	.	.	.	.	.	.	.	.	.	.	.	.	.	.	.	.	.	.
N19	.	.	.	.	.	.	.	.	.	.	.	A	.	.	.	.	.	.	.	.	.	.	.	C	.	.	.	.	.	.	.	.
N20	.	.	.	.	.	.	.	.	-	-	-	-	-	-	-	-	-	-	-	-	-	-	-	-	-	-	-	-	-	.	.	.
N21	.	.	.	.	.	.	.	.	.	.	.	A	.	.	.	.	.	.	.	.	.	.	.	C	.	.	.	.	.	.	.	.
N22	.	.	.	.	.	.	.	.	.	.	.	.	.	.	.	.	.	.	.	.	.	.	.	.	.	.	.	.	.	.	.	.
N23	.	.	.	.	.	.	.	.	.	.	.	A	.	.	.	.	.	.	.	.	.	.	.	C	.	.	.	.	.	.	.	.
N24	.	.	.	.	.	.	.	.	.	.	.	-	-	-	-	-	-	-	-	-	-	-	-	-	-	-	-	-	-	-	-	-
N25	.	.	.	.	.	.	.	.	G	.	.	-	-	-	-	-	-	-	-	-	-	-	-	-	-	-	-	-	-	-	-	-
N26	.	.	.	.	.	.	.	.	.	.	.	A	.	.	.	.	.	.	.	.	.	.	.	C	.	.	.	.	.	.	.	.
N27	.	.	.	.	.	.	.	.	.	.	.	A	.	.	.	.	.	.	.	.	.	.	.	C	.	.	.	.	.	.	.	.
N28	.	.	.	.	.	.	.	.	.	.	.	A	.	.	.	.	.	.	.	.	.	.	.	C	.	.	.	.	.	.	.	.
N29	-	-	-	-	-	-	-	-	-	-	-	-	-	-	-	-	-	-	-	-	-	-	-	-	-	-	-	-	-	-	-	-
N30	.	.	.	.	.	.	.	.	.	.	.	A	.	.	.	.	.	.	.	.	.	.	.	C	.	.	.	.	.	.	.	.
N31	.	.	.	.	.	.	.	.	.	.	.	A	.	.	.	.	.	.	.	.	.	.	.	C	.	.	.	.	.	.	.	.
N32	.	.	.	.	.	.	.	.	.	.	.	-	-	-	-	-	-	-	-	-	-	-	-	-	-	-	-	-	-	-	-	-
N33	.	.	.	.	.	.	.	.	.	.	.	A	.	.	.	.	.	.	.	.	.	.	.	C	.	.	.	.	.	.	.	.
N34	.	.	.	.	.	.	.	.	.	.	.	A	.	.	.	.	.	.	.	.	.	.	.	C	.	.	.	.	.	.	.	.
N35	.	.	.	.	.	.	.	.	.	.	.	A	.	.	.	.	.	.	.	.	.	.	.	C	.	.	.	.	.	.	.	.
N36	.	.	.	.	.	.	.	.	.	.	.	A	.	.	.	.	.	.	.	.	.	.	.	C	.	.	.	.	.	.	.	.
N37	.	.	.	.	G	.	.	.	.	.	.	A	.	.	.	.	.	.	.	.	.	.	.	C	.	.	.	.	.	.	.	.
N38	.	.	.	.	.	.	.	.	.	.	.	A	.	.	.	.	.	.	.	.	.	.	.	C	.	.	.	.	.	.	.	.
N39	.	.	.	.	.	.	.	.	.	.	.	A	.	.	.	.	.	.	.	.	.	.	.	C	.	.	.	.	.	.	.	.
N40	-	-	-	-	-	-	-	-	-	-	-	-	-	-	-	-	-	-	-	-	-	-	-	-	-	-	-	-	-	-	-	-

Table SA2. (continued).

Haplo-type	Variable nucleotide site in alignment																														
	529	530	531	532	533	534	535	536	537	538	539	540	541	542	543	544	550	583	586	597	598	599	600	601	602	603	604	605	606	607	608	609
N1	T	T	G	T	T	A	G	T	G	G	A	A	A	C	A	T	-	C	C	T	T	A	G	G	G	G	T	A	T	T	T	A
N2	.	.	.	.	.	.	.	.	.	.	.	.	.	.	.	.	-	.	.	.	.	.	.	.	.	.	.	.	.	.	.	.
N3	.	.	.	.	.	.	.	.	.	.	.	.	.	.	.	.	-	.	.	.	.	.	.	.	.	.	.	.	.	.	.	.
N4	.	.	.	.	.	.	.	.	.	.	.	.	.	.	.	.	-	.	G	.	.	.	.	.	.	.	.	.	.	.	.	.
N5	.	.	.	.	.	.	.	.	.	.	.	.	.	.	.	.	-	.	.	.	.	.	.	.	.	.	.	.	.	.	.	.
N6	.	.	.	.	.	.	.	.	.	.	.	.	.	.	.	.	-	.	.	.	.	.	.	.	.	.	.	.	.	.	.	.
N7	.	.	.	.	.	.	.	.	.	.	.	.	.	.	.	.	-	.	.	.	.	.	.	.	.	.	.	.	.	.	.	.
N8	.	.	.	.	.	.	.	.	.	.	.	.	.	.	.	.	-	.	.	.	.	.	.	.	.	.	.	.	.	.	.	.
N9	.	.	.	.	.	.	.	.	.	.	.	.	.	.	.	.	-	.	.	.	.	.	.	.	.	.	.	.	.	.	.	.
N10	.	.	.	.	.	.	.	.	.	.	.	.	.	.	.	.	-	.	.	.	.	.	.	.	.	.	.	.	.	.	.	.
N11	.	.	.	.	.	.	.	.	.	.	.	.	.	.	.	.	-	.	.	.	.	.	.	.	.	.	.	.	.	.	.	.
N12	.	.	.	.	.	.	.	.	.	.	.	.	.	.	.	.	-	.	.	.	.	.	.	.	.	.	.	.	.	.	.	.
N13	.	.	.	.	.	.	.	.	.	.	.	.	.	.	.	.	-	.	.	.	.	.	.	.	.	.	.	.	.	.	.	.
N14	.	.	.	.	.	.	.	.	.	.	.	.	.	.	.	.	-	.	.	.	.	.	.	.	.	.	.	.	.	.	.	.
N15	.	.	.	.	.	.	.	.	.	.	.	.	.	.	.	.	-	.	.	.	.	.	.	.	.	.	.	.	.	.	.	.
N16	.	.	.	.	.	.	.	.	.	.	.	.	.	.	.	.	-	.	.	.	.	.	.	.	.	.	.	.	.	.	.	.
N17	.	.	.	.	.	.	.	.	.	.	.	.	.	.	.	.	-	.	.	.	.	.	.	.	.	.	.	.	.	.	.	.
N18	.	.	.	.	.	.	.	.	.	.	.	.	.	.	.	.	-	.	.	.	.	.	.	.	.	.	.	.	.	.	.	.
N19	.	.	.	.	.	.	.	.	.	.	.	.	.	.	.	.	-	.	G	.	.	.	.	.	.	.	.	.	.	.	.	.
N20	.	.	.	.	.	.	.	.	.	.	.	.	C	.	.	.	-	.	.	.	.	.	.	.	.	.	.	.	.	.	.	.
N21	.	.	.	.	.	.	.	.	.	.	.	.	.	.	.	.	-	T	.	.	.	.	.	.	.	.	.	.	.	.	.	.
N22	.	.	.	.	.	.	.	.	.	.	.	.	.	.	.	.	-	.	.	.	.	.	.	.	.	.	.	.	.	.	.	.
N23	.	.	.	.	.	.	.	.	.	.	.	.	.	.	.	.	-	.	G	-	-	-	-	-	-	-	-	-	-	-	-	-
N24	-	-	.	.	.	.	.	.	.	.	.	.	C	.	.	.	-	.	.	.	.	.	.	.	.	.	.	.	.	.	.	.
N25	-	-	.	.	.	.	.	.	.	.	.	.	C	.	.	.	-	.	.	.	.	.	.	.	.	.	.	.	.	.	.	.
N26	.	.	.	.	.	.	.	.	.	.	.	.	.	.	.	.	-	T	.	.	.	.	.	.	.	.	.	.	.	.	A	.
N27	.	.	.	.	.	.	.	.	.	.	.	.	.	.	.	.	-	T	.	.	.	.	.	.	.	.	.	.	.	.	.	.
N28	.	.	.	.	.	.	.	.	.	.	.	.	.	.	.	.	-	T	.	.	.	.	.	.	.	.	.	.	.	.	A	.
N29	-	-	-	-	-	.	.	.	.	.	.	.	C	.	.	.	-	.	.	.	.	.	.	.	.	.	.	.	.	.	.	.
N30	.	.	.	.	.	.	.	.	.	.	.	.	.	.	.	.	-	T	.	.	.	.	.	.	.	.	.	.	.	.	A	.
N31	.	.	.	.	.	.	.	.	.	.	.	.	.	.	.	.	-	T	.	.	.	.	.	.	.	.	.	.	.	.	.	.
N32	-	-	.	.	.	.	.	.	.	.	.	.	.	.	.	.	-	T	.	.	.	.	.	.	.	.	.	.	.	.	.	.
N33	.	.	.	.	.	.	.	.	.	.	.	.	.	.	.	.	-	T	.	.	.	.	.	.	.	.	.	.	.	.	.	.
N34	.	.	.	.	.	.	.	.	.	.	.	.	.	.	.	.	-	T	.	.	.	.	.	.	.	.	.	.	.	.	.	.
N35	.	.	.	.	.	.	.	.	.	.	.	.	.	.	.	.	-	T	.	.	.	.	.	.	.	.	.	.	.	.	A	.
N36	.	.	.	.	.	.	.	.	.	.	.	.	.	.	.	.	-	T	.	.	.	.	.	.	.	.	.	.	.	.	.	.
N37	.	.	.	.	.	.	.	.	.	.	.	.	.	.	.	.	-	.	.	.	.	.	.	.	.	.	.	.	.	.	.	.
N38	.	.	.	.	.	.	.	.	.	.	.	.	.	.	.	.	T	T	.	.	.	.	.	.	.	.	.	.	.	.	A	.
N39	.	.	.	.	.	.	.	.	.	.	.	.	.	.	.	.	-	T	.	.	.	.	.	.	.	.	.	.	.	.	A	.
N40	-	-	-	-	-	-	-	-	-	-	-	-	-	-	-	-	-	T	.	.	.	.	.	.	.	.	.	.	.	.	A	.

Table SA2. (continued).

Haplo-type	Variable nucleotide site in alignment																													
	6	6	6	6	6	6	6	6	6	6	6	6	6	6	6	6	6	6	6	6	6	6	6	7	7	7	7	7	7	7	7
	1	1	1	1	1	1	1	1	1	1	2	2	2	2	2	2	3	5	5	6	8	8	9	1	1	1	2	2	3	4	5
	0	1	2	3	4	5	6	7	8	9	1	3	4	5	6	8	9	4	7	0	6	8	6	7	8	9	0	4	1	6	4
N1	T	T	A	G	T	T	A	G	T	T	T	A	C	T	C	A	T	C	-	G	C	A	-	-	-	-	C	-	T	A	A
N2	.	.	.	.	.	.	.	.	.	.	.	.	.	.	.	.	.	.	-	.	.	.	-	-	-	-	.	-	.	.	.
N3	.	.	.	.	.	.	.	.	.	.	.	.	.	.	.	.	.	.	-	.	.	.	-	-	-	-	T	-	.	.	.
N4	.	.	.	.	.	.	.	.	.	.	.	C	.	.	.	.	.	.	-	.	.	.	-	-	-	-	.	-	.	.	.
N5	.	.	.	.	.	.	.	.	.	.	.	.	.	.	.	.	.	.	-	.	.	.	-	-	-	-	.	-	.	.	.
N6	.	.	.	.	.	.	.	.	.	.	.	.	.	.	.	.	.	.	-	.	.	.	-	-	-	-	.	-	.	.	.
N7	.	.	.	.	.	.	.	.	.	.	.	.	.	.	.	.	.	.	-	.	.	.	-	-	-	-	.	-	.	.	.
N8	.	.	.	.	.	.	.	.	.	.	.	.	.	.	.	.	.	C	.	-	.	.	.	-	-	-	-	T	-	.	-	-
N9	.	.	.	.	.	.	.	.	.	.	.	.	.	.	.	.	.	.	-	.	.	.	-	-	-	-	.	-	.	.	.
N10	.	.	.	.	.	.	.	.	.	.	.	.	.	.	.	.	.	.	-	.	.	.	-	-	-	-	.	-	.	.	.
N11	.	.	.	.	.	.	.	.	.	.	.	.	.	.	.	.	.	C	.	-	.	.	.	-	-	-	-	T	-	.	.	.
N12	.	.	.	.	.	.	.	.	.	.	.	.	.	.	.	.	.	.	.	-	.	.	T	-	-	-	-	.	-	.	.	.
N13	.	.	.	.	.	.	.	.	.	.	.	.	.	.	.	.	.	.	.	-	.	.	.	-	-	-	-	.	-	.	.	.
N14	.	.	.	.	.	.	.	.	.	.	.	.	.	.	.	.	.	.	.	-	.	.	.	-	-	-	-	T	-	.	.	.
N15	.	.	.	.	.	.	.	.	.	.	.	.	.	.	.	.	.	.	.	-	.	.	.	-	-	-	-	T	-	.	.	.
N16	.	.	.	.	.	.	.	.	.	.	.	.	.	.	.	.	.	.	.	-	.	.	.	-	-	-	-	.	-	.	.	.
N17	.	.	.	.	.	.	.	.	.	.	.	.	.	.	.	.	.	.	.	-	.	.	.	-	-	-	-	.	-	.	.	.
N18	.	.	.	.	.	.	.	.	.	.	.	.	.	.	.	.	.	.	.	-	.	.	.	-	-	-	-	.	-	.	.	.
N19	.	.	.	.	.	.	.	.	.	.	.	C	.	.	.	.	.	.	.	-	.	.	.	-	-	-	-	.	-	.	.	.
N20	.	.	.	.	.	.	.	.	.	.	.	.	.	.	.	.	G	.	A	-	.	.	.	-	-	-	-	.	-	.	.	.
N21	.	.	.	.	.	.	.	.	.	.	.	C	.	.	.	.	G	.	.	-	.	.	.	C	G	T	A	.	-	.	.	.
N22	.	.	.	.	.	.	.	.	.	.	.	.	.	.	.	.	.	.	.	-	.	.	.	-	-	-	-	T	-	.	.	.
N23	-	-	-	-	-	-	-	-	-	A	C	A	C	G	C	A	.	.	.	-	.	.	.	-	-	-	-	.	-	.	.	.
N24	A	.	.	.	.	.	.	.	.	.	.	.	.	.	.	.	G	.	.	-	.	.	.	-	-	-	-	.	-	.	.	.
N25	.	.	.	.	.	.	.	.	.	.	.	.	.	.	.	.	G	.	-	A	.	.	.	-	-	-	-	.	-	.	.	.
N26	.	.	.	.	.	.	.	.	.	.	.	C	.	.	.	.	.	.	.	-	.	.	.	-	-	-	-	.	-	.	.	.
N27	.	.	.	.	.	.	.	.	.	.	.	.	.	.	.	.	G	.	.	-	.	.	.	C	G	T	A	.	-	.	.	.
N28	.	.	.	.	.	.	.	.	.	.	.	C	.	.	.	.	.	.	.	-	.	.	.	C	G	T	A	.	-	.	.	.
N29	.	.	.	.	.	.	.	.	.	.	.	.	.	.	.	.	G	.	.	-	.	.	.	-	-	-	-	.	A	.	.	.
N30	.	.	.	.	.	.	.	.	.	.	.	C	.	.	.	.	.	.	.	-	.	.	.	C	G	T	A	.	-	.	.	.
N31	.	.	.	.	.	.	.	.	.	.	.	.	.	.	.	.	G	.	.	-	.	T	.	C	G	T	A	.	-	.	.	.
N32	.	.	.	.	.	.	.	.	.	.	.	.	.	.	.	.	G	.	.	-	.	.	.	-	-	-	-	.	-	.	.	.
N33	.	.	.	.	.	.	.	.	.	.	.	C	.	.	.	.	G	.	.	-	.	.	.	C	G	T	A	.	-	.	.	.
N34	.	.	.	.	.	.	.	.	.	.	.	.	.	.	.	.	G	.	.	-	.	.	.	C	G	T	A	.	-	.	.	.
N35	.	.	.	.	.	.	.	.	.	.	.	C	.	.	.	.	.	.	.	-	.	.	.	C	G	T	A	.	-	.	.	.
N36	.	.	.	.	.	.	.	.	.	.	.	.	.	.	.	.	G	.	.	-	.	.	.	C	G	T	A	.	-	.	.	.
N37	.	.	.	.	.	.	.	.	.	.	.	C	.	.	.	.	.	.	.	-	.	.	.	C	G	T	A	.	-	.	.	.
N38	.	.	.	.	.	.	.	.	.	.	.	C	.	.	.	.	.	.	.	-	.	.	.	C	G	T	A	.	-	C	.	.
N39	.	.	.	.	.	.	.	.	.	.	.	C	.	.	.	.	.	.	.	-	.	.	.	-	-	-	-	.	-	.	.	.
N40	.	.	.	.	.	.	.	.	.	.	.	C	.	.	.	.	.	.	.	-	T	.	.	C	G	T	A	.	-	.	.	.

Table SA2. (continued).

Haplo-type	Variable nucleotide site in alignment																														
	756	757	758	759	760	761	771	781	783	805	813	816	821	822	823	824	845	846	847	848	849	850	851	852	853	854	855	856	857	858	859	860
N1	A	A	A	-	-	-	C	A	T	G	G	G	-	-	-	-	-	-	-	-	-	-	-	-	-	-	-	-	-	-	-	-
N2	.	.	.	-	-	-	.	.	.	.	.	.	-	-	-	-	-	-	-	-	-	-	-	-	-	-	-	-	-	-	-	-
N3	.	.	-	-	-	-	.	.	.	.	.	A	-	-	-	-	-	-	-	-	-	-	-	-	-	-	-	-	-	-	-	-
N4	.	.	-	-	-	-	T	.	.	.	.	.	-	-	-	-	-	-	-	-	-	-	-	-	-	-	-	-	-	-	-	-
N5	.	.	.	-	-	-	.	.	.	.	.	.	-	-	-	-	-	-	-	-	-	-	-	-	-	-	-	-	-	-	-	-
N6	.	.	-	-	-	-	.	.	.	.	.	.	-	-	-	-	-	-	-	-	-	-	-	-	-	-	-	-	-	-	-	-
N7	.	.	.	-	-	-	.	.	.	.	.	A	-	-	-	-	-	-	-	-	-	-	-	-	-	-	-	-	-	-	-	-
N8	-	-	-	-	-	-	.	.	.	.	.	.	-	-	-	-	-	-	-	-	-	-	-	-	-	-	-	-	-	-	-	-
N9	.	.	.	-	-	-	.	.	.	.	.	.	-	-	-	-	-	-	-	-	-	-	-	-	-	-	-	-	-	-	-	-
N10	.	.	.	A	-	-	.	.	.	.	.	.	-	-	-	-	-	-	-	-	-	-	-	-	-	-	-	-	-	-	-	-
N11	.	.	.	-	-	-	.	.	.	.	.	.	-	-	-	-	-	-	-	-	-	-	-	-	-	-	-	-	-	-	-	-
N12	.	.	.	-	-	-	.	.	.	.	.	.	-	-	-	-	-	-	-	-	-	-	-	-	-	-	-	-	-	-	-	-
N13	.	.	.	-	-	-	.	.	.	.	.	.	-	-	-	-	-	-	-	-	-	-	-	-	-	-	-	-	-	-	-	-
N14	.	.	-	-	-	-	.	.	.	.	.	A	-	-	-	-	-	-	-	-	-	-	-	-	-	-	-	-	-	-	-	-
N15	.	.	-	-	-	-	.	.	.	.	.	A	-	-	-	-	-	-	-	-	-	-	-	-	-	-	-	-	-	-	-	-
N16	.	.	.	-	-	-	.	.	.	.	A	.	-	-	-	-	-	-	-	-	-	-	-	-	-	-	-	-	-	-	-	-
N17	.	.	.	-	-	-	.	.	.	.	.	.	-	-	-	-	-	-	-	-	-	-	-	-	-	-	-	-	-	-	-	-
N18	.	.	.	A	-	-	.	.	.	.	A	.	-	-	-	-	-	-	-	-	-	-	-	-	-	-	-	-	-	-	-	-
N19	.	.	-	-	-	-	T	.	.	.	.	.	-	-	-	-	-	-	-	-	-	-	-	-	-	-	-	-	-	-	-	-
N20	.	.	.	-	-	-	T	.	C	.	.	.	-	-	-	-	-	-	-	-	-	-	-	-	-	-	-	-	-	-	-	-
N21	.	.	.	A	-	-	T	.	.	.	.	.	A	G	C	T	-	-	-	-	-	-	-	-	-	-	-	-	-	-	-	-
N22	.	.	.	-	-	-	.	.	.	.	.	A	-	-	-	-	-	-	-	-	-	-	-	-	-	-	-	-	-	-	-	-
N23	.	.	.	A	-	-	T	.	C	.	.	.	-	-	-	-	-	-	-	-	-	-	-	-	-	-	-	-	-	-	-	-
N24	.	.	-	-	-	-	T	.	.	.	.	.	-	-	-	-	-	-	-	-	-	-	-	-	-	-	-	-	-	-	-	-
N25	.	.	.	A	-	-	T	.	C	.	.	.	-	-	-	-	-	-	-	-	-	-	-	-	-	-	-	-	-	-	-	-
N26	.	.	-	-	-	-	T	.	.	.	.	.	-	-	-	-	-	-	-	-	-	-	-	-	-	-	-	-	-	-	-	-
N27	.	.	.	A	A	A	T	.	.	.	.	.	-	-	-	-	-	-	-	-	-	-	-	-	-	-	-	-	-	-	-	-
N28	.	-	-	-	-	-	T	.	.	.	.	.	A	G	C	T	-	-	-	-	-	-	-	-	-	-	-	-	-	-	-	-
N29	.	.	.	-	-	-	T	.	.	.	.	.	-	-	-	-	-	-	-	-	-	-	-	-	-	-	-	-	-	-	-	-
N30	.	-	-	-	-	-	T	.	.	.	.	.	A	G	C	T	-	-	-	-	-	-	-	-	-	-	-	-	-	-	-	-
N31	.	.	-	-	-	-	T	.	.	.	.	.	A	G	C	T	-	-	-	-	-	-	-	-	-	-	-	-	-	-	-	-
N32	.	.	-	-	-	-	T	.	.	.	.	.	-	-	-	-	-	-	-	-	-	-	-	-	-	-	-	-	-	-	-	-
N33	.	.	.	A	A	A	T	.	.	.	.	.	-	-	-	-	-	-	-	-	-	-	-	-	-	-	-	-	-	-	-	-
N34	.	.	.	A	A	A	T	.	.	.	.	.	-	-	-	-	-	-	-	-	-	-	-	-	-	-	-	-	-	-	-	-
N35	.	-	-	-	-	-	T	.	.	.	.	.	A	G	C	T	-	-	-	-	-	-	-	-	-	-	-	-	-	-	-	-
N36	.	.	.	A	A	A	T	.	.	.	.	.	-	-	-	-	-	-	-	-	-	-	-	-	-	-	-	-	-	-	-	-
N37	.	.	.	A	A	-	T	.	.	.	.	.	-	-	-	-	-	-	-	-	-	-	-	-	-	-	-	-	-	-	-	-
N38	.	.	-	-	-	-	T	.	.	.	.	.	-	-	-	-	-	-	-	-	-	-	-	-	-	-	-	-	-	-	-	-
N39	.	-	-	-	-	-	T	G	.	.	.	.	-	-	-	-	-	-	-	-	-	-	-	-	-	-	-	-	-	-	-	-
N40	.	-	-	-	-	-	T	.	.	T	.	.	-	-	-	-	A	C	T	A	T	A	A	T	A	G	T	G	T	A	T	C

Table SA2. (continued).

Haplo-type	Variable nucleotide site in alignment																												
	861	862	863	864	865	866	867	868	869	870	871	872	873	874	875	876	877	878	879	880	881	882	883	884	885	886	887	888	889	894
N1	-	-	-	-	-	-	-	-	-	-	-	-	-	-	-	-	-	-	-	-	-	-	-	-	-	-	-	-	-	G
N2	-	-	-	-	-	-	-	-	-	-	-	-	-	-	-	-	-	-	-	-	-	-	-	-	-	-	-	-	-	.
N3	-	-	-	-	-	-	-	-	-	-	-	-	-	-	-	-	-	-	-	-	-	-	-	-	-	-	-	-	-	.
N4	-	-	-	-	-	-	-	-	-	-	-	-	-	-	-	-	-	-	-	-	-	-	-	-	-	-	-	-	-	.
N5	-	-	-	-	-	-	-	-	-	-	-	-	-	-	-	-	-	-	-	-	-	-	-	-	-	-	-	-	-	.
N6	-	-	-	-	-	-	-	-	-	-	-	-	-	-	-	-	-	-	-	-	-	-	-	-	-	-	-	-	-	.
N7	-	-	-	-	-	-	-	-	-	-	-	-	-	-	-	-	-	-	-	-	-	-	-	-	-	-	-	-	-	.
N8	-	-	-	-	-	-	-	-	-	-	-	-	-	-	-	-	-	-	-	-	-	-	-	-	-	-	-	-	-	.
N9	-	-	-	-	-	-	-	-	-	-	-	-	-	-	-	-	-	-	-	-	-	-	-	-	-	-	-	-	-	.
N10	-	-	-	-	-	-	-	-	-	-	-	-	-	-	-	-	-	-	-	-	-	-	-	-	-	-	-	-	-	.
N11	-	-	-	-	-	-	-	-	-	-	-	-	-	-	-	-	-	-	-	-	-	-	-	-	-	-	-	-	-	.
N12	-	-	-	-	-	-	-	-	-	-	-	-	-	-	-	-	-	-	-	-	-	-	-	-	-	-	-	-	-	.
N13	-	-	-	-	-	-	-	-	-	-	-	-	-	-	-	-	-	-	-	-	-	-	-	-	-	-	-	-	-	.
N14	-	-	-	-	-	-	-	-	-	-	-	-	-	-	-	-	-	-	-	-	-	-	-	-	-	-	-	-	-	.
N15	-	-	-	-	-	-	-	-	-	-	-	-	-	-	-	-	-	-	-	-	-	-	-	-	-	-	-	-	-	.
N16	-	-	-	-	-	-	-	-	-	-	-	-	-	-	-	-	-	-	-	-	-	-	-	-	-	-	-	-	-	.
N17	-	-	-	-	-	-	-	-	-	-	-	-	-	-	-	-	-	-	-	-	-	-	-	-	-	-	-	-	-	.
N18	-	-	-	-	-	-	-	-	-	-	-	-	-	-	-	-	-	-	-	-	-	-	-	-	-	-	-	-	-	.
N19	-	-	-	-	-	-	-	-	-	-	-	-	-	-	-	-	-	-	-	-	-	-	-	-	-	-	-	-	-	.
N20	-	-	-	-	-	-	-	-	-	-	-	-	-	-	-	-	-	-	-	-	-	-	-	-	-	-	-	-	-	.
N21	-	-	-	-	-	-	-	-	-	-	-	-	-	-	-	-	-	-	-	-	-	-	-	-	-	-	-	-	-	.
N22	-	-	-	-	-	-	-	-	-	-	-	-	-	-	-	-	-	-	-	-	-	-	-	-	-	-	-	-	-	.
N23	-	-	-	-	-	-	-	-	-	-	-	-	-	-	-	-	-	-	-	-	-	-	-	-	-	-	-	-	-	.
N24	-	-	-	-	-	-	-	-	-	-	-	-	-	-	-	-	-	-	-	-	-	-	-	-	-	-	-	-	-	.
N25	-	-	-	-	-	-	-	-	-	-	-	-	-	-	-	-	-	-	-	-	-	-	-	-	-	-	-	-	-	.
N26	-	-	-	-	-	-	-	-	-	-	-	-	-	-	-	-	-	-	-	-	-	-	-	-	-	-	-	-	-	.
N27	-	-	-	-	-	-	-	-	-	-	-	-	-	-	-	-	-	-	-	-	-	-	-	-	-	-	-	-	-	A
N28	-	-	-	-	-	-	-	-	-	-	-	-	-	-	-	-	-	-	-	-	-	-	-	-	-	-	-	-	-	.
N29	-	-	-	-	-	-	-	-	-	-	-	-	-	-	-	-	-	-	-	-	-	-	-	-	-	-	-	-	-	.
N30	-	-	-	-	-	-	-	-	-	-	-	-	-	-	-	-	-	-	-	-	-	-	-	-	-	-	-	-	-	.
N31	-	-	-	-	-	-	-	-	-	-	-	-	-	-	-	-	-	-	-	-	-	-	-	-	-	-	-	-	-	.
N32	-	-	-	-	-	-	-	-	-	-	-	-	-	-	-	-	-	-	-	-	-	-	-	-	-	T	A	A	T	.
N33	-	-	-	-	-	-	-	-	-	-	-	-	-	-	-	-	-	-	-	-	-	-	-	-	-	-	-	-	-	.
N34	-	-	-	-	-	-	-	-	-	-	-	-	-	-	-	-	-	-	-	-	-	-	-	-	-	-	-	-	-	.
N35	-	-	-	-	-	-	-	-	-	-	-	-	-	-	-	-	-	-	-	-	-	-	-	-	-	-	-	-	-	.
N36	-	-	-	-	-	-	-	-	-	-	-	-	-	-	-	-	-	-	-	-	-	-	-	-	-	-	-	-	-	.
N37	-	-	-	-	-	-	-	-	-	-	-	-	-	-	-	-	-	-	-	-	-	-	-	-	-	-	-	-	-	.
N38	-	-	-	-	-	-	-	-	-	-	-	-	-	-	-	-	-	-	-	-	-	-	-	-	-	-	-	-	-	.
N39	-	-	-	-	-	-	-	-	-	-	-	-	-	-	-	-	-	-	-	-	-	-	-	-	-	-	-	-	-	.
N40	C	G	T	A	G	T	A	C	T	A	T	A	A	G	T	A	T	A	A	T	A	A	T	G	T	T	A	A	T	.

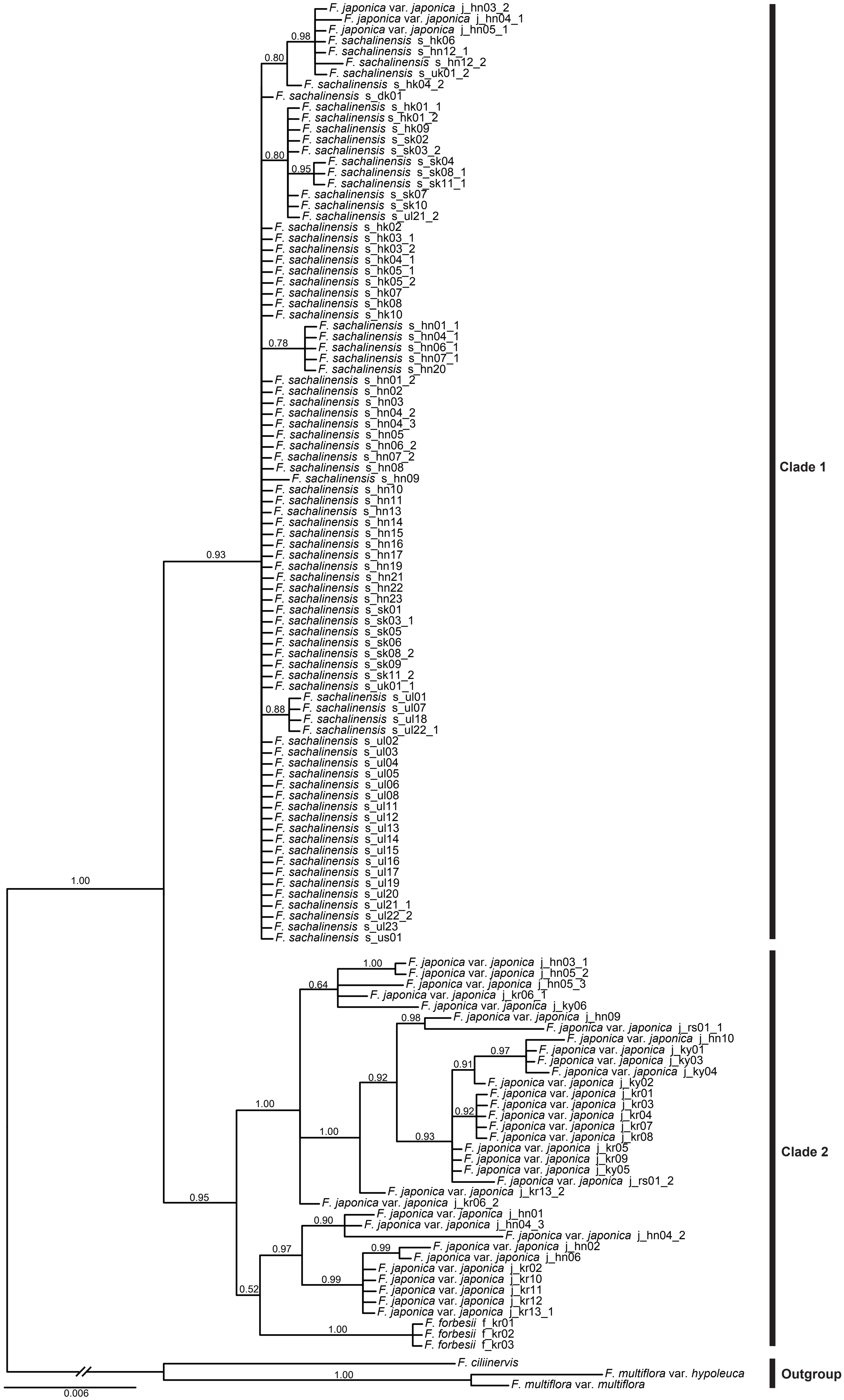

Figure SA1. Bayesian majority-rule consensus tree for individuals of F. sachalinensis and closely related taxa based on the second intron of the nDNA *LEAFY* region. Numbers above branches are Bayesian posterior probabilities. Accession numbers correspond to those in Figure 1 and Supplementary Table 1. Clade 1 comprises all accessions of *F. sachalinensis*. Clade 2 is sister to Clade 1 and consists of *F. japonica* var. *japonica* and *F. forbesii* accessions.
